# Deep sequencing reveals transcriptome re-programming of *Polygonum multiflorum* thunb. roots to the elicitation with methyl jasmonate

**DOI:** 10.1007/s00438-015-1112-9

**Published:** 2015-09-05

**Authors:** Hongchang Liu, Wei Wu, Kai Hou, Junwen Chen, Zhi Zhao

**Affiliations:** College of Agronomy, Sichuan Agricultural University, Chengdu, 611130 Sichuan China; Guizhou Key (Engineering) Laboratory for Propagation and Cultivation of Medicinal Plants, Guiyang, 550025 Guizhou China

**Keywords:** *Polygonum multiflorum* thunb., Transcriptional profile, Methyl jasmonate, Real-time fluorescence quantitative PCR

## Abstract

The phytohormone methyl jasmonate (MeJA) has been successfully used as an effective elicitor to enhance production of stilbenoid which is induced in plants as a secondary metabolite possibly in defense against herbivores and pathogens. However, the mechanism of MeJA-mediated stilbenoid biosynthesis remains unclear. Genomic information for *Polygonum multiflorum* Thunb. (*P*. *multiflorum*) is currently unavailable. To obtain insight into the global regulation mechanism of MeJA in the steady state of stilbene glucoside production (26 h after MeJA elicitation), especially on stilbene glucoside biosynthesis, we sequenced the transcriptomes of MeJA-treated and untreated *P. multiflorum* roots and obtained more than 51 million clean reads, from which 79,565 unigenes were obtained by de novo assembly. 56,972 unigenes were annotated against databases including Nr, Nt, Swiss-Prot, KEGG and COG. 18,677 genes expressed differentially between untreated and treated roots. Expression level analysis indicated that a large number of genes were associated with plant–pathogen interaction, plant hormone signal transduction, stilbenoid backbone biosynthesis, and phenylpropanoid biosynthesis. 15 known genes involved in the biosynthesis of stilbenoid backbone were found with 7 genes showing increased transcript abundance following elicitation of MeJA. The significantly up (down)-regulated changes of 70 genes in stilbenoid biosynthesis were validated by qRT-PCR assays and PCR product sequencing. According to the expression changes and the previously proposed enzyme functions, multiple candidates for the unknown steps in stilbene glucoside biosynthesis were identified. We also found some genes putatively involved in the transcription factors. This comprehensive description of gene expression information could greatly facilitate our understanding of the molecular mechanisms of MeJA-mediated stilbenoid biosynthesis in *P*. *multiflorum* roots. Our results shed new light on the global regulation mechanism by which MeJA regulates the physiology of *P. multiflorum* roots and is helpful to understand how MeJA elicits other plant species besides *P. multiflorum*.

**Electronic supplementary material:**

The online version of this article (doi:10.1007/s00438-015-1112-9) contains supplementary material, which is available to authorized users.

## Introduction

The traditional Chinese herb, *P*. *multiflorum*, has been used in the preparation of herbal medicines in oriental countries such as China, Japan and Korea for thousands of years because of its pharmacological functions (Jung et al. 2011; Luo et al. 2011). *P*. *multiflorum* can produce a number of chemicals with pharmaceutical properties (Han et al. 2009). Among them, stilbene glucoside was reported to be a very promising antioxidation, purgation, tonic and antiaging agent and now is commercially used as a hair-darkening agent and as a potent kidney tonic (Wu et al. 2012). Stilbene glucoside was mainly extracted from the root of *P*. *multiflorum* but yields are low; approximately 1.5 % of the dry root weight (Jung et al. 2011). With growing demand for the stilbene glucoside for medical and commercial use, harvesting from root cannot meet the demand and also raises serious ecological concerns.

Great efforts have been taken to increase the production of stilbene glucoside using biotransformation (Yan et al. 2007) and micropropagation (Lin et al. 2003) which currently suffers from low yields. Some compounds are known to stimulate the production of stilbenoid when added to the medium. Among them, MeJA has been found to significantly elicit the production of stilbenoid in the cultured cells of grapevine (Jeandet et al. 2002; Vannozzi et al. 2012; Vezzulli et al. 2007). However, the detailed biological mechanism of MeJA stimulation of stilbenoid production and concomitant transcriptome changes associated with response to MeJA remain poorly understood. A detailed knowledge of the biosynthesis of stilbene glucoside and its regulation by MeJA is required before directed bioengineering.

Next-generation sequencing technologies have been proved to be rapid and cost-effective means to analyze the genome and transcriptome in non-model species. Improvement in de novo assembly of high-throughput sequencing data and relative accurate estimation of gene expression levels makes this approach also powerful in quantifying gene expression (Sun et al. 2013).

To get a comprehensive understanding of the global regulation mechanism of MeJA on stilbene glucoside biosynthesis in steady state of stilbene glucoside production, we sequenced the transcriptomes of *P*. *multiflorum* roots at 26 h without or with the elicitation of MeJA when the stilbene glucoside accumulation is observed. Comparing the gene expression profiles of these two samples revealed that 7 out of 15 known genes in stilbenoid backbone biosynthesis had higher transcript levels by addition of MeJA. In addition to the increased transcript abundance of homologous recombination and mRNA surveillance pathway, we found decreased transcript levels of genes involved in spliceosome, RNA transport, pentose and glucuronate interconversions, etc., which provides insights into the mechanism by which MeJA inhibits cell growth while significantly inducing the production of stilbene glucoside.

## Materials and methods

### Plant material and root-irrigating treatment by MeJA solution on plantlets

Plants of cultivated *P. multiflorum* were propagated from vine cuttings in the greenhouse. They were grown in controlled conditions at 25/20 °C day/night, with 75 % relative humidity and a 16-h photoperiod (350 μmol/m^2^/s). Watering once every 3 days with Hoagland's nutrient solution (Mahmoudi et al. 2012). Forty-five-day-old plants with five to seven leaves were used for MeJA solution root-irrigation experiments.

The final concentration of MeJA solution with wetting agent Triton X-100 at 0.1 % (v/v) was 0.25 mM. Five hundred milliliters of this solution were added to a 10-l flowerpot containing 4 kg vermiculite and expanded perlite (m/m = 1:1) and two *P*. *multiflorum* plantlets. Control plantlets were irrigated with the Triton solution at 0.1 %. Mixing root samples with 30 plants in triplicate were collected at 26 h after treatment, immediately frozen in liquid nitrogen and stored at −80 °C until RNA extraction. All chemicals were purchased from Sigma Corporation (http://www.sigmaaldrich.com/china-mainland.html) unless otherwise stated.

### Total RNA isolation, cDNA library construction and sequencing

Total RNA was extracted following the protocol of the E.Z.N.A.™ Plant RNA Kit (http://omegabiotek.com/index.php). The quality and quantity of total RNA were analyzed using a Thermo Scientific Multiskan GO (Thermo Scientific Multiskan GO, www.thermoscientific.com), gel electrophoresis, and Agilent G2939A (Agilent RNA 6000 Nano Kit). Equal quantities of high-quality RNA from each sample were pooled for cDNA synthesis.

The mRNA-seq library was constructed using an mRNA-seq Sample Preparation Kit (Illumina Inc., Sandiego, CA, USA) following the manufacturer’s instruction. The poly-(A) mRNA was isolated from the total RNA samples with poly-T oligo-attached magnetic beads. An RNA Fragmentation Kit (Ambion, Austin, TX, USA) was used to cleave the mRNA into short fragments, which were then used as templates to reverse-transcribe first strand using random hexamer primers and reverse transcriptase (http://www.takara-bio.com) (Takara). Second-strand cDNA was synthesized in a reaction containing buffer, dNTPs, RNase H and DNA polymerase I (Takara). A paired-end library was synthesized using the Genomic Sample Preparation Kit (Illumina) according to the manufacturer’s instructions. Short fragments were purified with the MinElute PCR Purification Kit (QIAGEN, Dusseldorf, Germany) and eluted in 10 µL of EB buffer (QIAGEN). The short fragments were connected with sequencing adaptors, and the desired fragments (200 ± 25 bp) were separated by agarose gel electrophoresis and purified with a Gel Extraction Kit (Axygen Biosciences, Central Avenue Union City, CA, USA). Finally, the paired-end sequencing library was constructed by PCR amplification (15 cycles) and sequenced using the Illumina Solexa Genome Analyzer IIx sequencing platform. Data analysis and base calling were performed using Illumina instrument software (supplementary data Fig. S1).

### Sequence data analysis, assembly, and annotation

All raw reads were cleaned by removing adapter sequences, low-quality sequences with ambiguous bases “N”, and reads with more than 10 % Q <20 bases. The qualified reads were extended into contigs with Trinity software through the overlap between the sequences. Then the contigs were connected into transcript sequences, according to paired-end information of the sequences, which recovers full-length transcripts across a broad range of expression levels, with sensitivity similar to methods that rely on genome alignments (Li et al. 2010; Grabherr et al. 2011). The overlap settings used for this assembly were 31 bp and 80 % similarity, group pairs distance was set to 500 (maximum length expected between fragment pair), with all the other parameters set to their default values. We selected the longest transcriptions from the potential assembled component alternative splicing transcripts as unigene sequences of our samples. We quantified transcript levels in reads per kilobase of exon mode per million mapped reads (RPKM) (Mortazavi et al. 2008). The RPKM measure of read density reflects the molar concentration of a transcript in the starting sample by normalizing for RNA length and for the total read number in the measurement. Genes with high expression levels were screened and listed (supplementary data Fig. S2).

The optimal assembly results were chosen according to the assembly evaluation. Then, clustering analysis was performed to achieve a unigene database which comprised the potential alternative splicing transcripts.

The assembled sequences were compared against the NCBI Nr, Nt, and Swiss-Prot databases using BLASTn (version 2.2.14) with an *E* value of 10^−5^. Gene names were assigned to each assembled sequence based on the highest scoring BLAST hit. Searches were limited to the first 10 significant hits for each query to increase computational speed. Open reading frames (ORF) were predicted using the ‘‘getorf’’ program of EMBOSS software package (Rice et al. 2000), with the longest ORF extracted for each unigene. To annotate the assembled sequences with gene ontology (GO) terms describing biological processes, molecular functions, and cellular components, the Swiss-Prot BLAST results were imported into Blast2GO to obtain the GO annotations of the unigenes (Conesa et al. 2005, 2008). The obtained annotation was enriched and refined using ANNEX (Myhre et al. 2006; Ye et al. 2006). The unigene sequences were also aligned to the Clusters of Orthologous Group (COG) database to predict and classify functions (Tatusov et al. 2000). Kyoto Encyclopedia of Genes and Genomes (KEGG) pathways were assigned to the assembled sequences using the online KEGG Automatic Annotation Server (http://www.genome.jp/kegg/kaas/). The bi-directional best hit method was used to obtain KEGG Orthology assignment (Moriya et al. 2007) (supplementary data Fig. S3).

If results of different databases conflict with each other, a priority order of Nr, Nt, Swiss-Prot, KEGG and COG should be followed when deciding sequence direction of unigenes. When a unigene happens to be unaligned to none of the above databases, a software named ESTScan (Iseli et al. 1999) will be introduced to decide its sequence direction. For unigenes with sequence directions, we provide their sequences from 5′ end to 3′ end; for those without any direction, we provide their sequences from assembly software.

### Unigene expression difference analysis

This analysis aims to predict genes with different expression levels, and then carry out GO functional analysis and KEGG pathway analysis on them. Expression Annotation: The calculation of unigene expression uses FPKM method (Mortazavi et al. 2008). Identification of differentially expressed genes (DEGs): Referring to “The significance of digital gene expression profiles” (Audic and Claverie 1997; Benjamini et al. 2001) which has been cited hundreds of times, we have developed a rigorous algorithm to identify differentially expressed genes between two samples.

### Real-time PCR and PCR products sequencing validation

To validate RNA-seq gene expression results, quantitative Real-Time PCR (qRT-PCR) was run using SYBR^®^*Premix Ex Taq*™ II (Perfect Real Time) (http://www.takara-bio.com) and real-time PCR thermal cycler (qTower 2.2, analytikjena, Germany, www.analytik-jena.com) using the same cDNA samples used in the RNA-seq experiment. A first-strand cDNA fragment was synthesized from total RNAs using the Takara PrimerScript™ RT Reagent Kit with gDNA eraser (Perfect Real Time) (http://www.takara-bio.com). Gene-specific primers were designed for target transcript sequences and *UBQ14*, *UBQ4*-*1* and *SAMS* DNA sequence as internal controls (supplementary data Table S1, S2, S3). The comparative threshold cycle method was used to calculate the relative gene expression (Livak and Schmittgen 2001). Each real-time PCR was carried out three times. PCR products of 13 target transcript sequences were sequencing for comparing sequence similarity with RNA-seq data.

## Results

### Illumina sequencing and de novo assembly of sequence reads in *P. multiflorum*

Illumina high-throughput second-generation sequencing was carried out to obtain the transcriptomes of *P. multiflorum* roots under non-elicited and elicited conditions. The sequencing produced 58,233,066 and 61,114,668 raw reads from the non-elicited and elicited sample transcriptomes, respectively. After removing the adaptor sequences, empty reads and low-quality sequences (reads with more than 10 % Q20 bases), we obtained respectively high-quality reads from the two transcriptomes shown in Table 1. From the non-elicited sample, a total of 135,230 contigs with length not less than 200 nt were generated; the N50 size and the mean size are 651 and 356 nt, respectively. Table 2 and supplementary data Fig. S4 show the length distribution of contigs ranging from 200 nt to more than 3000 nt. From the elicited sample, 117,736 contigs have the N50 size of 610 nt and the mean size of 350 nt. Although most contigs were between 200 and 400 bp, 17.08 % nucleotides of control (23,097 contigs) and 16.88 % nucleotides of treatment (19,869 contigs) were greater than 500 bp in length (supplementary data Fig. S5).

**Table 1:** The statistics of RNA-seq data

Samples	Total raw reads	Total clean reads	Total clean nucleotides (nt)	Q20 %	*N* %	GC %
Control	58,233,066	51,569,864	4641,287,760	97.46 %	0.00 %	48.97 %
Treatment	61,114,668	53,924,012	4853,161,080	97.33 %	0.00 %	49.86 %

**Table 2:** Statistical comparison of contigs and unigenes between non-elicited and elicited *P. multiflorum* roots

Gene type	Sample	Total number	Total length (nt)	Mean length (nt)	N50	Total consensus sequences	Distinct clusters	Distinct singletons
Contig	Control	135,230	48,184,343	356	651	–	–	–
	Treatment	117,736	41,211,915	350	610	–	–	–
Unigene	Control	89,440	57,785,503	646	1024	89,440	31,545	57,895
	Treatment	78,409	47,285,726	603	929	78,409	25,690	52,719
	All	79,565	62,762,185	789	1187	79,565	33,718	45,847

The contigs further assembled with paired-end joining and gap filling to produce 89,440 unigenes with an N50 of 1024 bp (18,020 of which larger than 1000 bp) for control; 78,409 unigenes with an N50 of 929 bp (13,944 of which larger than 1000 bp) for treatment and total 79,565 unigenes with an N50 of 1187 bp (21,318 of which larger than 1000 bp) for two samples (Table 2; supplementary data Fig. S6). These results indicate that the assembly and contig joining succeeded in processing a large amount of short reads from *P*. *multiflorum* root samples with relatively little redundancy.

Among the 79,565 assembled unigenes, 48,626 unigenes were ≥500 bp and 24,190 were ≥1000 bp, with a mean unigenes length of 789 bp and N50 of 1187 bp (supplementary data Fig. S6). We analyzed the ratio of the gap’s length to the length of assembled unigenes. The results revealed that the majority of the unigenes, which accounted for 90.0 % of total unigenes, showed gap lengths that were less than 2.5 % of the total length, suggesting that our sequence data was highly suitable for further analysis.

### Functional annotation of predicted proteins

Annotation analysis provides information of gene expression and functional annotation of all unigenes in each sample. Functional annotation consists of protein functional annotation, pathway annotation, COG functional annotation and GO functional annotation. Unigenes were annotated with the databases of Nr, Nt, Swiss-Prot, KEGG, COG and GO. Then counted the number of unigenes annotated with each database. The result is summarized in Table 3. To gain a preliminary insight into the functions of the unigenes obtained, functional annotation of the *P*. *multiflorum* transcriptome sequences was first performed against GenBank Nr using *E* value 10^−5^ as a cutoff value. Due to the lack of relevant species genome information in NCBI data base and the relatively short length of distinct gene sequences, only 55,031 out of 79,565 unigenes (69.16 %) were detected to have homologs. We found 25 and 29 unigenes with the relatively high abundance of more than 1000 transcripts per million (relative abundance) in the non-elicited and elicited samples, respectively (supplementary data Table S4), among which hypothetical protein MTR (hypothetical protein MTR_5g051130 [Medicago truncatula]) and VITISV (hypothetical protein VITISV_043424 [Vitis vinifera]), major latex protein homolog and lectin were highly expressed in both datasets. Compared with the non-elicited sample, the elicited sample contains unigenes encoding endo-1,3-1,4-beta-d-glucanase, LEA (Desiccation protectant protein Lea14 homolog [Glycine max]), harpin-induced protein, mitochondrial protein, type III polyketide synthase 3, nucleic acid-binding protein, tumor-related protein, polygalacturonase-inhibiting protein, etc. These genes expressed abundantly after elicited by MeJA. Interestingly, we note multiple unigenes with unknown annotation, which would be intriguing to investigate what functions these unigenes perform, given the high levels that these genes possess.

**Table 3:** Statistics of annotation results

Database	Nr	Nt	Swiss-Prot	KEGG	COG	GO	All
Unigene number	55,031	39,984	35,996	32,790	20,701	39,043	56,972

**Fig. 1:**
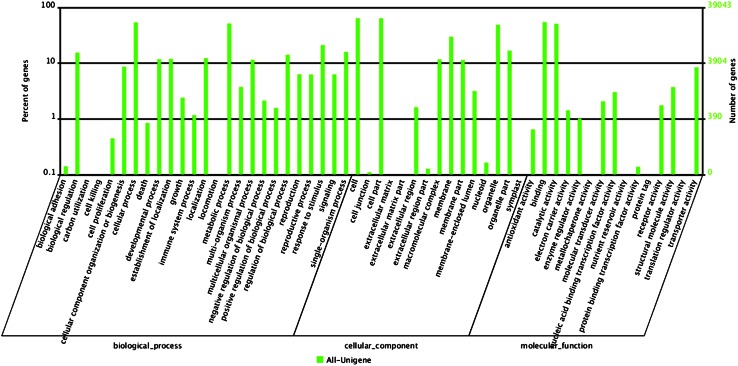
Functional annotation of assembled unigenes based on gene ontology (GO) categorization

After obtaining putative gene functions assigned by homology searches, we predicted the biological processes, cellular components and molecular functions that the proteins belong to, by association with GO information using the obtained annotation from GenBank Nr database. The results showed that most proteins encoded by these unigenes take part in the biological processes including cellular process, metabolic process, response to stimulus and biological regulation. As for the cellular component domain, a majority of products of unigenes involved in cell, cell part, organelle and membrane. For the molecular function domains, genes involved in binding and catalytic activity are dominant (Fig. 1).

**Fig. 2:**
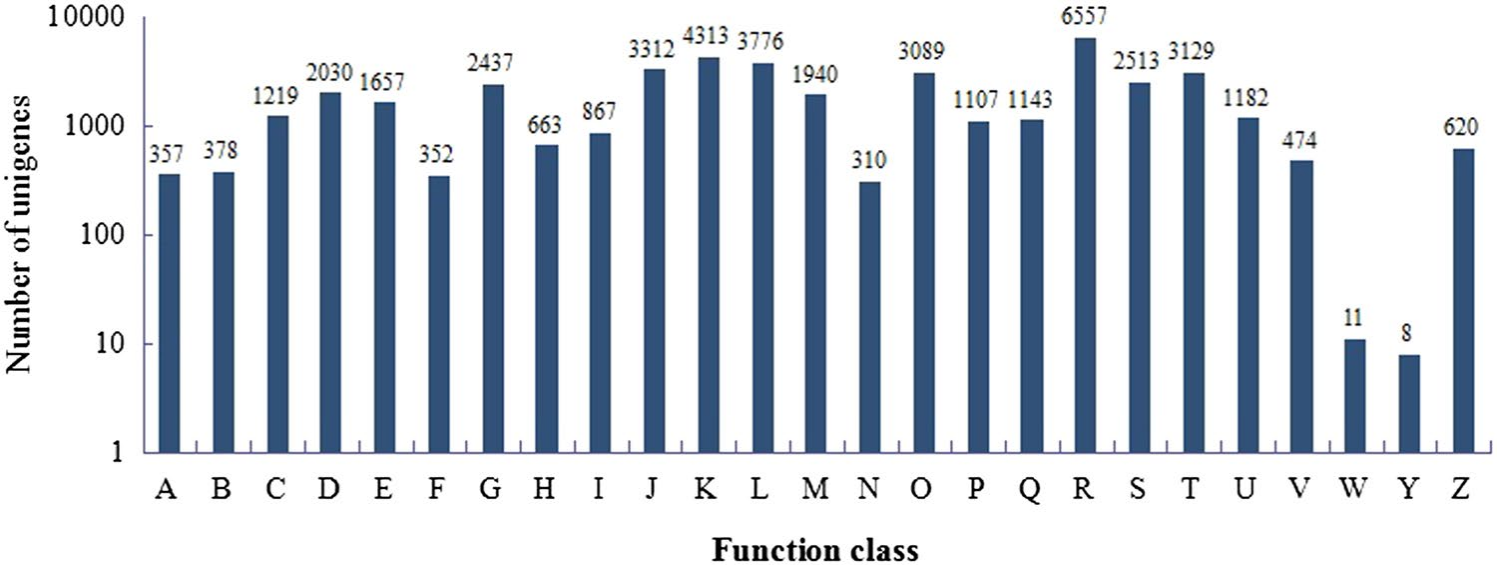
Clusters of orthologous groups (COG) classification of unigenes in non-elicited and elicited samples. *A* RNA processing and modification, *B* chromatin structure and dynamics, *C* energy production and conversion, *D* cell cycle control, cell division, chromosome partitioning, *E* amino acid transport and metabolism, *F* nucleotide transport and metabolism, *G* carbohydrate transport and metabolism, *H* coenzyme transport and metabolism, *I* lipid transport and metabolism, *J* translation, ribosomal structure and biogenesis, *K* transcription, *L* replication, recombination and repair, *M* cell wall/membrane/envelope biogenesis, *N* cell motility, *O* posttranslational modification, protein turnover, chaperones, *P* inorganic ion transport and metabolism, *Q* secondary metabolism biosynthesis, transport and catabolism, *R* general function prediction only, *S* function unknown, *T* signal transduction mechanisms, *U* intracellular trafficking, secretion, and vesicular transport, *V* defense mechanisms, *W* extracellular structures, *Y* nuclear structure, *Z* cytoskeleton

To facilitate the functional classification of the unigenes, we employed their homologs’ functional classification information in COG database, which emphasizes a classification of genes in an evolutionary view using complete genome sequences. All of the 79,565 unigenes were aligned to the COGs database to predict and classify possible functions. According to COG database, 20,701 unigenes were classified into 25 different functional classes, which were represented by A to Z. Compared with the non-elicited samples, the elicited samples showed more unigenes in general function prediction only (R), transcription (K) and replication, recombination and repair (L), but less expressed genes in extracellular structure (W) and nuclear structure (Y) (Fig. 2).

To understand the metabolism pathways in non-elicited and elicited *P*. *multiflorum* root samples, we performed BLASTX search against KEGG database to map the unigenes to canonical pathways. A total of 32,790 unigenes in the two datasets refer to 125 KEGG pathways. Compared with the non-elicited samples, the elicited samples have slightly more unigenes in the pathways including metabolic pathway (24.61 %), biosynthesis of secondary metabolites (10.85 %), plant–pathogen interaction (6.07 %) and plant hormone signal transduction (supplementary data Table S5). The annotations of these unigenes showed a significant transcriptional complexity and provided valuable gene expression information in the transcriptome of *P*. *multiflorum* roots.

### DEG analysis elicited by MeJA

A total of 18,677 genes were shown to be differentially expressed in response to MeJA elicitation. Compared with the control, the expression levels of 4535 DEGs were up-regulated and 14,142 DEGs down-regulated in MeJA-treated *P*. *multiflorum* roots. Examining the ten most up-regulated and the ten most down-regulated genes, five of the up-regulated genes have defined functions, including “actin and related proteins” [log2 (FC) = 15.4125], “Ca^2+^-binding protein (EF-Hand superfamily)” [log2 (FC) = 14.5471] and “F0F1-type ATP synthase, subunit a” [log2 (FC) = 13.9881]; and six down-regulated genes have defined functions, such as “Glutaredoxin and related proteins” [log2 (FC) = −12.7662], “Secreted trypsin-like serine protease” [log2 (FC) = −13.4580] and “DNA-directed RNA polymerase, beta’ subunit/160 kD subunit” [log2 (FC) = −12.9418].

**Fig. 3:**
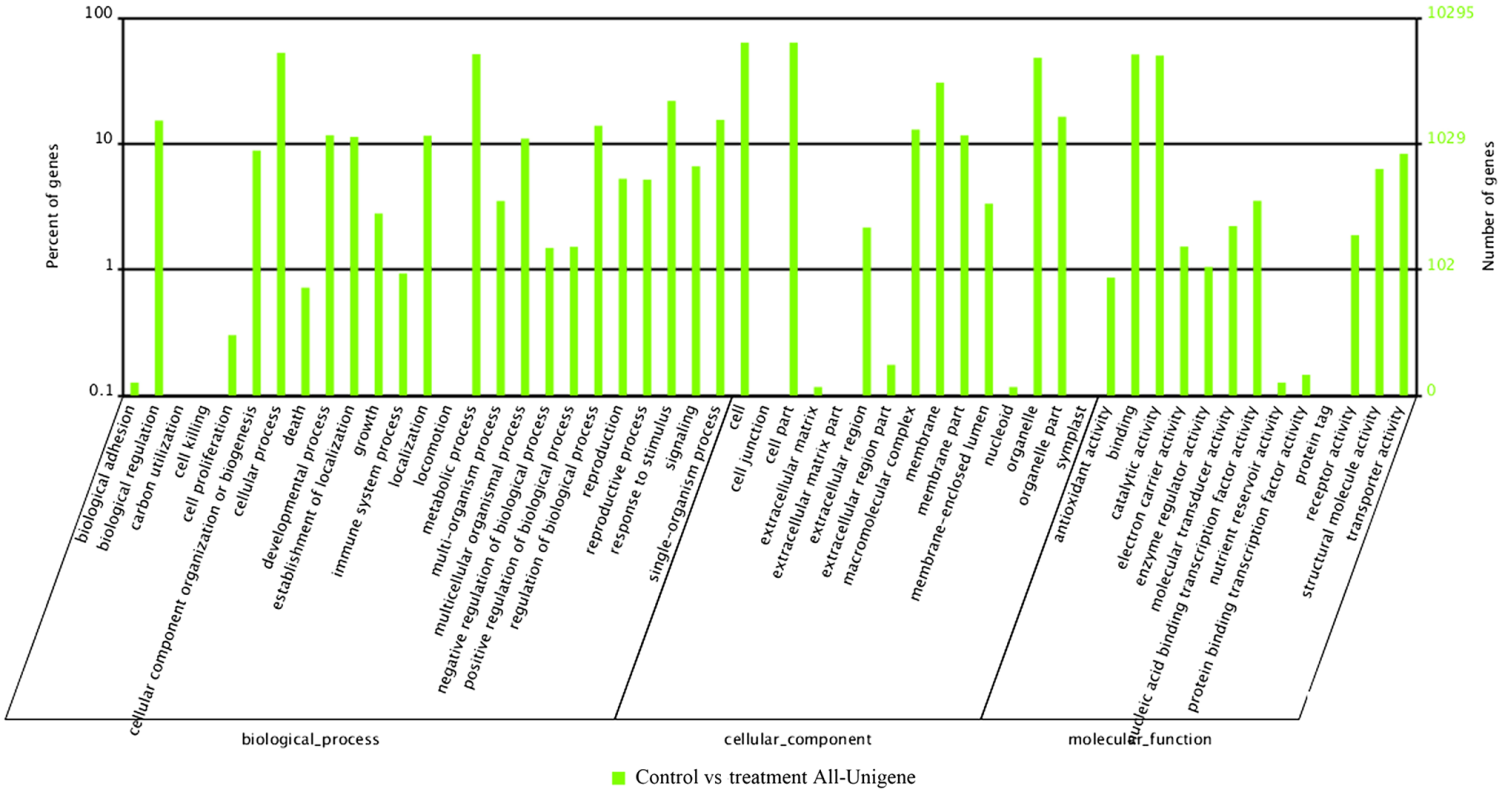
Histogram presentation of clusters of orthologous groups (COG) classification of unigenes in control vs treatment

GO classification analysis of 18,677 DEGs showed that a large number of DEGs were dominant in seven terms, e.g., “cell part” and “cell” (Fig. 3). All DEGs were then mapped in the KEGG database to search for genes involved in “metabolic pathways” or “Biosynthesis of secondary metabolites”. Control and treatment samples were analyzed by KEGG, we found that the “plant–pathogen interaction”, “plant hormone signal transduction” and “endocytosis” had the most significant changes. 8936 DEGs were annotated by KEGG, and this annotation revealed significant enrichment for genes found in metabolic pathways (2441 DEGs, 27.32 %), biosynthesis of secondary metabolites (1079 DEGs, 12.07 %), and Ribosome (644 DEGs, 7.21 %). Only 5 genes were annotated in lipoic acid metabolism pathway with 2 DEGs, whose quantity was the least in the 125 metabolic pathways of *P.* *multiflorum* root after induced by MeJA, followed by C_5_-branched dibasic acid metabolism (total 14, 7 DEGs), sulfur relay system (total 19, 4 DEGs), anthocyanin biosynthesis (total 17, 7 DEGs), glycosphingolipid biosynthesis-globo series (total 24, 4 DEGs), photosynthesis-antenna proteins (total 24, 5 DEGs) and indole alkaloid biosynthesis (total 24, 8 DEGs) (supplementary data Table S5). These results indicate that these metabolic pathways might be insensitive to MeJA. This annotation of genes differentially induced by MeJA will provide a valuable resource for investigating specific processes, functions and pathways responding to MeJA in *P*. *multiflorum* roots.

### Comparison of the expression levels of genes between non-elicited and elicited *P. multiflorum* roots

To investigate the response of elicitation with MeJA in *P*. *multiflorum* roots, the expression levels of each unigene in the two samples were estimated by using RSEM software, which can quantify transcript abundance from RNA-seq data without a reference genome (Li and Dewey 2011), and then performed statistical analysis implemented in EdgeR (Robinson et al. 2010) using the mapped read numbers for each unigene calculated by RSEM. About 31.11 % unigenes (8105) including 2293 up-regulated and 5812 down-regulated showed at least twofold changes (adjusted *p* value ≤0.05). We found 2249 unigenes with no less than fourfold changes including 753 up-regulated and 1496 down-regulated (adjusted *p* value ≤0.05), which are from 37 pathways. Unigenes with the greatest down-regulated change (12.9418 fold) involved in more than one pathway, such as RNA polymerase, metabolic pathway, purine metabolism and pyrimidine metabolism; however, unigenes with the greatest up-regulated change (15.4125 fold) only involved in phagosome.

We investigated the expression changes of genes in the view of pathways mentioned above by calculating the genes with fourfold changes. Among the 37 pathways, six showed increased transcript abundance, 15 of them displayed decreased transcript abundance, and other pathways had unigenes with both increased and decreased in transcript abundance. Pathways that showed increased transcript abundance with at least two unigenes included homologous recombination and mRNA surveillance pathway. Interestingly, ten pathways, namely ribosome, metabolic pathway, ether lipid metabolism, plant–pathogen interaction, phagosome, plant hormone signal transduction, spliceosome, RNA transport, flavonoid biosynthesis, pentose and glucuronate interconversions, had at least 10 unigenes with distinct changes and all but one had increased transcript abundance. Pathways which possessed decreased transcript abundance included for example circadian rhythm-plant, phenylpropanoid biosynthesis, carotenoid biosynthesis, alpha-linolenic acid metabolism, etc. In addition, all the unigenes in the pathways of regulation of autophagy, ubiquitin-mediated proteolysis, glucosinolate biosynthesis, nitrogen metabolism, sphingolipid metabolism had lower transcript abundance (supplementary data Table S5).

### MeJA-responsive transcription factors in *P. multiflorum* roots

Transcription factors (TFs) regulate the spatio-temporal expression of responsive genes to abiotic and biotic stresses in the defense mechanisms of plants (Yanagisawa 1998; Riechmann et al. 2000). Our sequence data showed that 1207 unigenes were annotated to encode putative TFs, including 113 up-regulated and 327 down-regulated genes. These 440 unigenes differentially expressed in response to MeJA elicitation were largely represented by the TF families regulating stress responses and secondary metabolism in plants, e.g., ERF superfamily (93 members), WRKY superfamily (75 members), bHLH (66 members), AP2 superfamily (56 members), MYB superfamily (46 members). The ten most differentially up-regulated unigenes encoding TFs were EFR109, MYB48, WRKY 41, bHLH118, bHLH35, AP2/ERF and B3 domain-containing RAV1, GATA8, WRKY 40, EFR095 and MYB39, whose “OS (original species)” are all *Arabidopsis thaliana*; and the ten most significantly down-regulated unigenes encoding TFs were WRKY 23, PosF21, AIL6, GATA21, B3 domain-containing VRN1, GATA12, bHLH128, GATA9 and BEE1, whose “OS” are all *Arabidopsis thaliana* except for MYB1R1 whose “OS” is *Solanum tuberosum*. Abundant unigenes encoding putative TFs responding to MeJA elicitation showed that transcription regulation may relate to MeJA-mediated response network in *P*. *multiflorum* roots.

### The MeJA-responsive activity in plant–pathogen interaction pathway

**Fig. 4:**
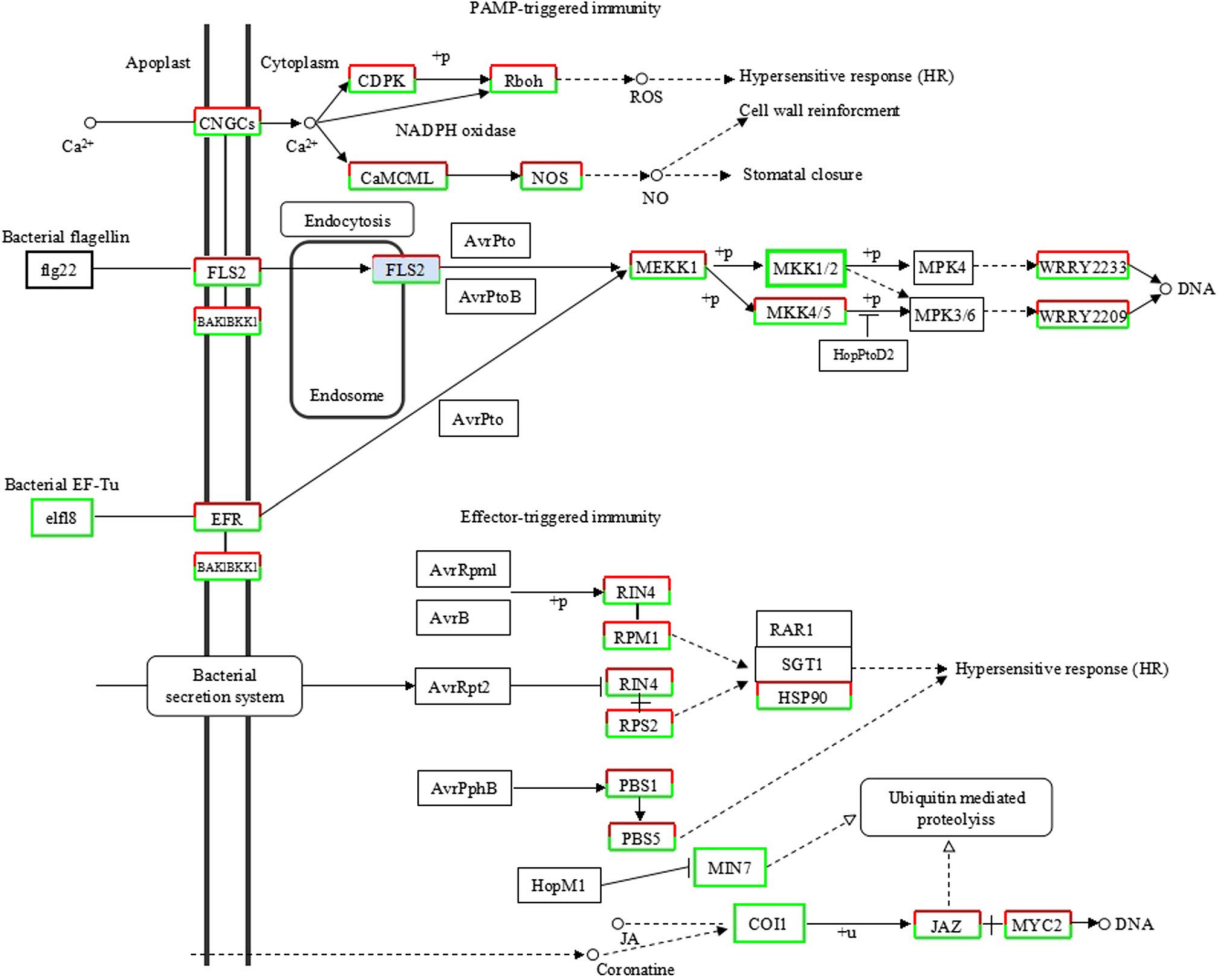
Plant–pathogen interaction elicited by MeJA in *P*. *multiflorum* roots

MeJA has been applied to induce defensive responses equivalent to insect or pathogen attacks. Exogenous MeJA has been successfully used to enhance plant resistance against several insects and pathogens (Gaige et al. 2010; Ku et al. 2014; Moreira et al. 2009). Our RNA-seq data showed that 1990 unigenes, including 627 DEGs with 232 up-regulated and 395 down-regulated, were annotated as having roles in the plant–pathogen interaction (supplementary data Table S5). Genes coding for disease-resistance protein RPM1, disease-resistance protein RPS2, disease-resistance protein RPS5, and pathogenesis-related protein 1 were heavily represented in the “plant–pathogen interaction” pathway (Fig. 4). The effect of MeJA on the induction of these biotic/abiotic stress associated genes suggests that they can be exploited to improve plant resistance to pathogens and pests.

### MeJA affects plant hormone signal transduction

MeJA can induce jasmonate (JA) biosynthesis and steer the JA signaling pathway to activate several defense mechanisms and hormone biosynthesis in plant cells (Santino et al. 2013; Wasternack and Hause 2013). Our RNA-seq data showed that 1746 unigenes, including 621 DEGs, were annotated as having roles in “plant hormone signal transduction” pathway, and what is more, the unigene quantity in the “plant hormone signal transduction” is the fourth in all 125 metabolic pathways, second only to metabolic pathways (8071 unigenes, 2441 DEGs), biosynthesis of secondary metabolites (3557 unigenes, 1079 DEGs) and plant–pathogen interaction (1990 unigenes, 627 DEGs) (supplementary data Table S5). So many unigenes in the “plant hormone signal transduction” pathway expressed differentially under MeJA elicitation showed that these unigenes may have certain relationship with MeJA. What is more, the results of expressing differentially of these unigenes are definitely worth exploring.

### MeJA affects phenylpropanoid biosynthesis and genes in the main chain of stilbenoid backbone biosynthesis

**Fig. 5:**
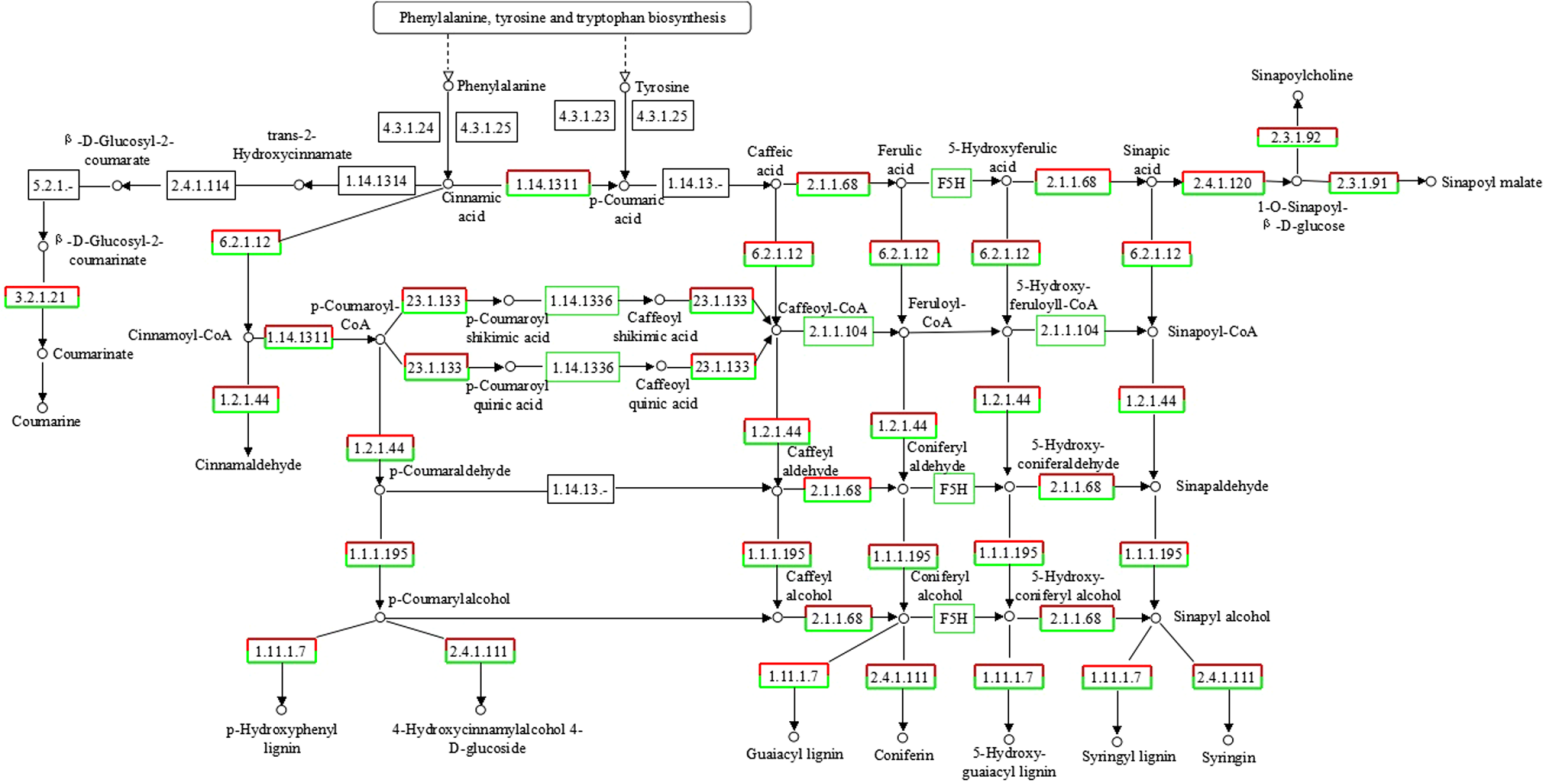
“Phenylpropanoid biosynthesis” induced by MeJA in *P*. *multiflorum* roots

**Fig. 6:**
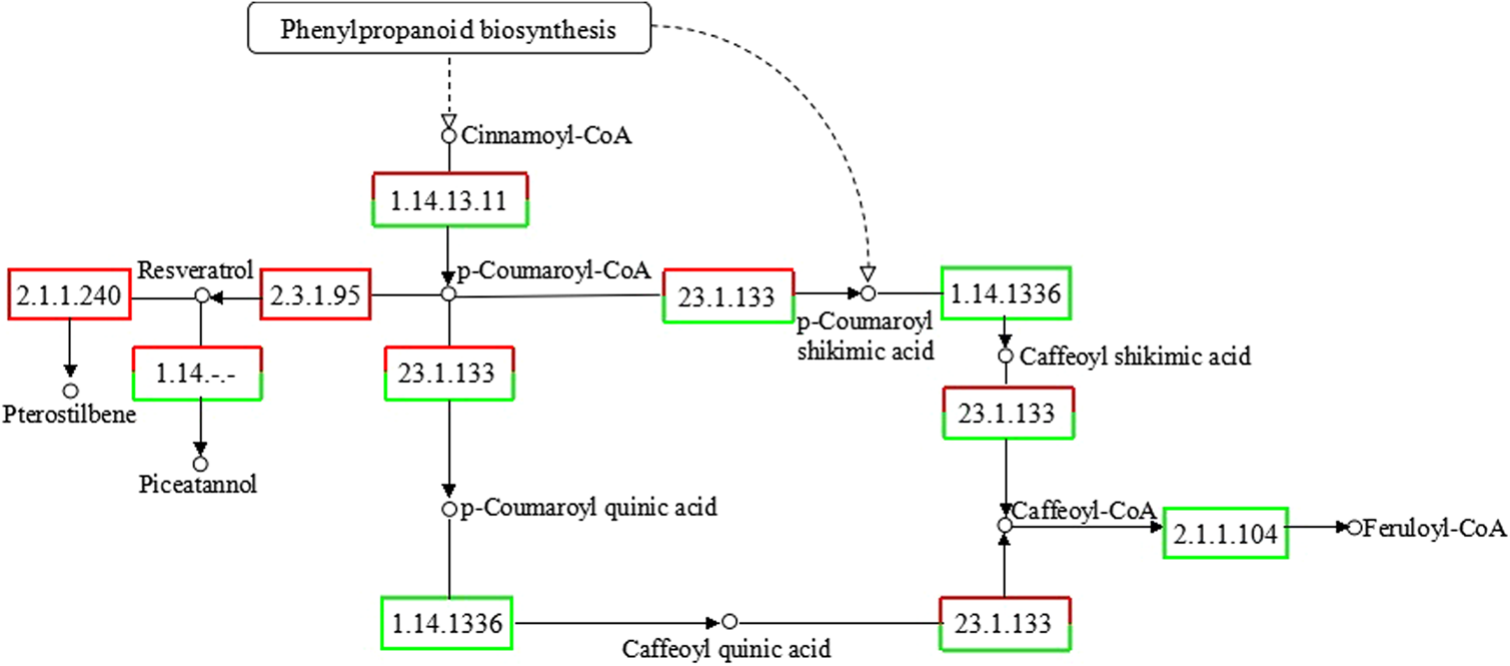
“Stilbenoid, diarylheptanoid and gingerol biosynthesis” induced by MeJA in *P*. *multiflorum* roots

The annotation of *P*. *multiflorum* root RNA-seq data showed that 622 unigenes, including 216 DEGs, were involved in the biosynthesis of phenylpropanoids (supplementary data Table S5). These compounds are used in plant defense to create physical and chemical barriers against infection, and as molecules involved in the local and systemic signaling of defense gene induction (Dixon et al. 2002). Unigenes related to key enzymes in the phenylpropanoid metabolism pathway (Weisshaar and Jenkins 1998) were differential expressed in non-elicited and elicited *P*. *multiflorum* roots, such as trans-cinnamate 4-monooxygenase (C_4_H), 4-coumarate-CoA ligase (4CL), cinnamoyl-CoA reductase (CCR), cinnamyl-alcohol dehydrogenase, peroxidase, chalcone synthase (CHS), and polyketide reductase (PKR) (Figs. 5, 6). These results suggest that the effect of MeJA on defense responses, hormone biosynthesis and phenylpropanoid biosynthesis identified in other plant species (Jeandet et al. 2002; Vezzulli et al. 2007) is also found in *P*. *multiflorum* roots.

Plant stilbenoids are a small group of phenylpropanoids, which have been detected in at least 72 unrelated plant species and accumulate in response to biotic and abiotic stresses such as infection, wounding, UV-C exposure and treatment with chemicals. Stilbenoids are formed via the phenylalanine/polymalonate-route, the last step of which is catalyzed by the enzyme stilbene synthase (STS), a type III polyketide synthase (PKS) (Jeandet et al. 2002; Vannozzi et al. 2012). Stilbenoids are the most abundant and structurally diverse group of plant secondary metabolites and derived from the universal precursor p-coumarate (Hanhineva et al. 2009). Because the stilbenoid backbone biosynthesis pathway is incomplete in the KEGG database, to find the genes responsible for the biosynthesis of stilbenoid backbone, we manually identified all genes involved in this pathway by reciprocal BLAST search against the transcriptome using previous reported enzymes as queries (Fig. 6). Fifteen unigenes which corresponding to phenylalanine ammonia-lyase (PAL), C_4_H, 4CL, STS were obtained by manual BLAST search. The four key enzymes, especially C_4_H, 4CL and STS, could regulate the flux of stilbenoid backbone biosynthesis (Bavaresco et al. 2012). The largest increase in transcript abundance observed in this pathway was STS (3.4324- and 3.7158-fold), C_4_H (2.8402-fold). Two unique sequences of 4CL show similar transcript abundance changes, 1.5875 and 1.4674 times, respectively. Unigenes of PAL showed no differential expression between the elicited and non-elicited samples.

### Validation of DEGs with qRT-PCR analysis and PCR product sequencing

To validate the RNA-seq gene expression results, we randomly selected 70 representative genes encoding 7 groups of enzyme types and designed specific primers for qRT-PCR. Consistent with RNA-seq analysis, the relative expression pattern of 70 genes was identified as being significantly up-regulated or down-regulated in the MeJA-elicited samples as compared to the non-elicited samples (supplementary data Table S6). Furthermore, PCR products of 13 randomly selected representative genes were sequenced, and the sequences of which were found to be consistent with the RNA-Seq data (supplementary data Table S7). These results further supported that the RNA-seq data are reliable.

## Discussion

MeJA regulates a diverse set of physiological and developmental processes, and addition of MeJA can significantly induce the production of secondary metabolites such as terpenoids, phenylpropanoids, and alkaloids (Pauwels et al. 2008). Our experimental data showed that 0.20 mM MeJA solution could significantly increase stilbene glucoside production in *P*. *multiflorum* (supplementary data Fig.S7). Furthermore, some mechanisms of the MeJA-elicited biosynthesis of secondary metabolites have been elucidated in some plant species including *Catharanthus roseus* and tobacco (Verma et al. 2014).

It was found that *P*. *multiflorum* roots at 26 h after MeJA elicitation were characterized by mRNA levels and genes involved in stilbenoid synthesis were found, which was consistent with the results reported in *Vitis vinifera* cell, *Vitis vinifera* cv. Barber cell and cultivated grapevine under MeJA treatment conditions (Belhadj et al. 2006; Krisa et al. 1999; Tassoni et al. 2005; Vezzulli et al. 2007). According to RNA-seq results of MeJA-elicited and non-elicited roots of *P*. *Multiflorum*, more than 51 million sequence reads were generated and each of the two samples was represented by at least 51 million sequence reads in which the tag density was sufficient for qualitative analysis of gene expression (Mortazavi et al. 2008). We identified and annotated these sequences by using a series of bioinformatics tools to produce 79,565 unigenes including 18,677 differentially expressed in response to MeJA. Meanwhile, as far as the number of contig and unigene is concerned, some genes in the *P*. *multiflorum* root were suppressed after treatment by 0.25 mM MeJA solution, which indicated that this concentration may be harmful to cell growth of *P*. *multiflorum* and wherein the mechanism is worthy of further study. The known genes involved in the stilbenoid backbone pathway could be identified, which suggested that the stilbenoid backbone pathway existed in the *P*. *multiflorum* roots. Many studies had shown that application of exogenous MeJA induces the stilbenoid backbone pathway in several plants (Bavaresco et al. 2012; Jeandet et al. 2002; Krisa et al. 1999; Tassoni et al. 2005; Vannozzi et al. 2012; Vezzulli et al. 2007), including *Vitis vinifera,Vitis vinifera* cv. Barber., *V. vinifera* cv. Pinot noir, and *V. vinifera* cv Shiraz etc. In this study with *P*. *multiflorum* roots, our RNA-seq and qRT-PCR results showed that the expression of presumptive stilbenoid backbone pathway genes (C_4_H, 4CL, STS) was consistent with the mechanism seen in other plants, suggesting exogenous application of MeJA could mediate stilbenoid biosynthesis and stilbenoid backbone pathway, thereby regulating a series of downstream genes in *P*. *multiflorum* roots.

In the present study, the known genes found in pathways that regulate stilbenoid synthesis, a large number of genes with known or predicted functions involved in several metabolic pathways, plant hormone signal transduction and phenylpropanoid biosynthesis, as well as many genes encoding TFs were all shown to be induced in response to MeJA. In plant cells, one major regulatory mechanism of secondary metabolite production is via the control of the expression of TFs that in turn regulate biosynthesis genes (Broun 2004; Yanagisawa 1998), e.g., the ORCA3 TF regulates several JA-responsive genes in MeJA-inducible indole alkaloid biosynthesis in *Catharanthus roseus* (Van der fits and Memelink 2000). Similarly, stress responsive TFs have been suggested to be involved in stilbenoid biosynthesis. Our sequence results also showed that many genes encoding TFs were differentially expressed in response to MeJA elicitation. These MeJA-responsive TFs may directly or indirectly regulate the production or activity of stilbenoid biosynthetic enzymes; thus characterization of the DEGs which encode TFs might shed light on the regulation of stilbenoid biosynthesis in *P*. *multiflorum.*

Although structural elucidation of stilbene glucoside, which is one of the most important stilbenoids in *P. multiflorum*, has been extensively studied, its biosynthesis still needs to be further elucidated (Lv et al. 2006, 2008, 2010), whose skeleton is based on trans-resveratrol structure (3,5,4′-trihydroxy stilbene) (Jeandet et al. 2002). In terms of chemical structure, hydroxylation and glycosylation may be the final two steps for stilbene glucoside based on trans-resveratrol. Our RNA-seq data provided available candidate genes of the final two steps (supplementary data Table S8), which will be validated in our next study. The molecular cloning and characterization of these candidate genes will further elucidate the stilbene glucoside biosynthesis pathway in *P*. *multiflorum*.

In conclusion, using Illumina sequencing technology, we investigated the poly (A) + transcriptome of the MeJA-elicited *P*. *multiflorum* roots versus non-elicited roots and produced 79,565 assembled unigenes with 56,972 unigenes that could be annotated compared to other known genes from other plant species. Analysis of the annotated unigenes from *P*. *multiflorum* roots showed a significant transcriptional complexity and provided more information about MeJA response. Metabolic pathways involved in biosynthesis of TFs, plant–pathogen interaction, plant hormone signal transduction and stilbenoid backbone were bioinformatically reconstructed in *P*. *multiflorum*. Additionally, the nucleotide sequences obtained through transcriptome sequencing serve as a substantial contribution to existing sequence resources of *P*. *multiflorum*. Particularly, the transcriptome data provided candidates of presumptive biosynthetic enzymes for the remaining steps in stilbene glucoside biosynthesis pathways. Further analysis of the *P*. *multiflorum* genes annotated to TFs will help us understand regulation patterns upon MeJA elicitation and the molecular mechanisms of MeJA-mediated stilbene glucoside biosynthesis in *P*. *multiflorum* roots. In summary, this transcriptome data will serve as an important public information platform to accelerate research of MeJA-responsive networks and the regulatory mechanisms of stilbene glucoside biosynthesis.

## Electronic supplementary material

Supplementary material 1 (JPEG 1162 kb) Fig. S1 Pipeline of experiments

Supplementary material 2 (JPEG 1233 kb) Fig. S2 Assembly process

Supplementary material 3 (JPEG 1428 kb) Fig. S3 Pipeline of bioinformatics analysis

Supplementary material 4 (JPEG 469 kb) Fig. S4 Length distribution of contigs from non-elicited and elicited samples

Supplementary material 5 (JPEG 631 kb) Fig. S5 Length distribution of unigenes from non-elicited and elicited samples

Supplementary material 6 (JPEG 506 kb) Fig. S6 Length distribution of all assembled unigenes from non-elicited and elicited samples

Supplementary material 7 (JPEG 346 kb) Fig. S7 Effects of MeJA on stilbene glucoside content in *P*. *multiflorum*.

Supplementary material 8 (DOC 28 kb)

Supplementary material 9 (DOC 158 kb)

Supplementary material 10 (DOC 248 kb)

Supplementary material 11 (DOC 179 kb)

Supplementary material 12 (DOC 255 kb)

Supplementary material 13 (DOC 411 kb)

Supplementary material 14 (DOC 109 kb)

Supplementary material 15 (DOC 209 kb)
