# Supplementary figures and images for: Deep sequencing reveals transcriptome re-programming of Polygonum multiflorum thunb. roots to the elicitation with methyl jasmonate
Source: Mol Genet Genomics. 2015 Sep 5;291:337–48. doi: 10.1007/s00438-015-1112-9 (PMC4729805; doi:10.1007/s00438-015-1112-9)

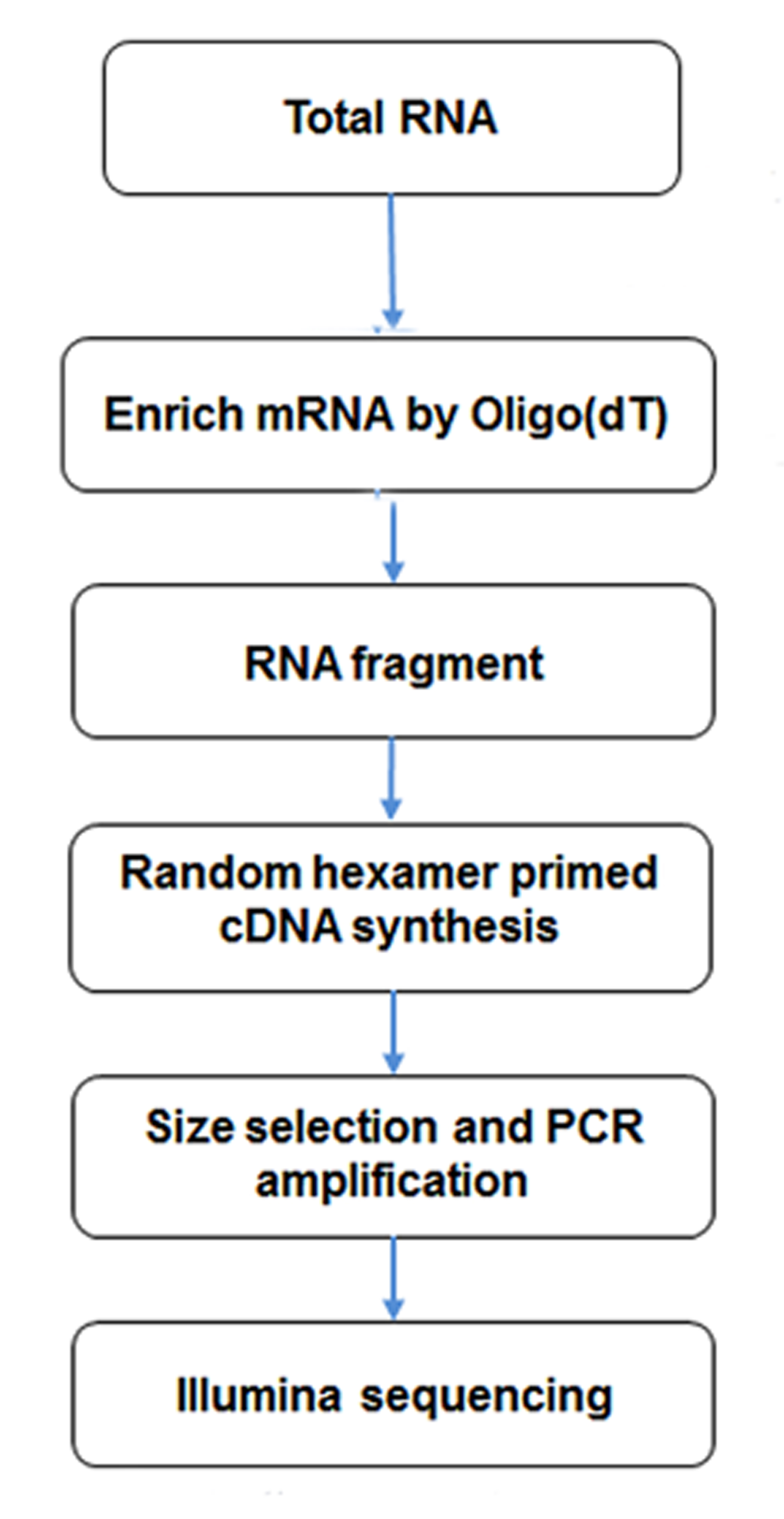

Supplement: Supplementary file 1 — Supplementary material 1 (JPEG 1162 kb) Fig. S1 Pipeline of experiments [file 438_2015_1112_MOESM1_ESM.jpg]

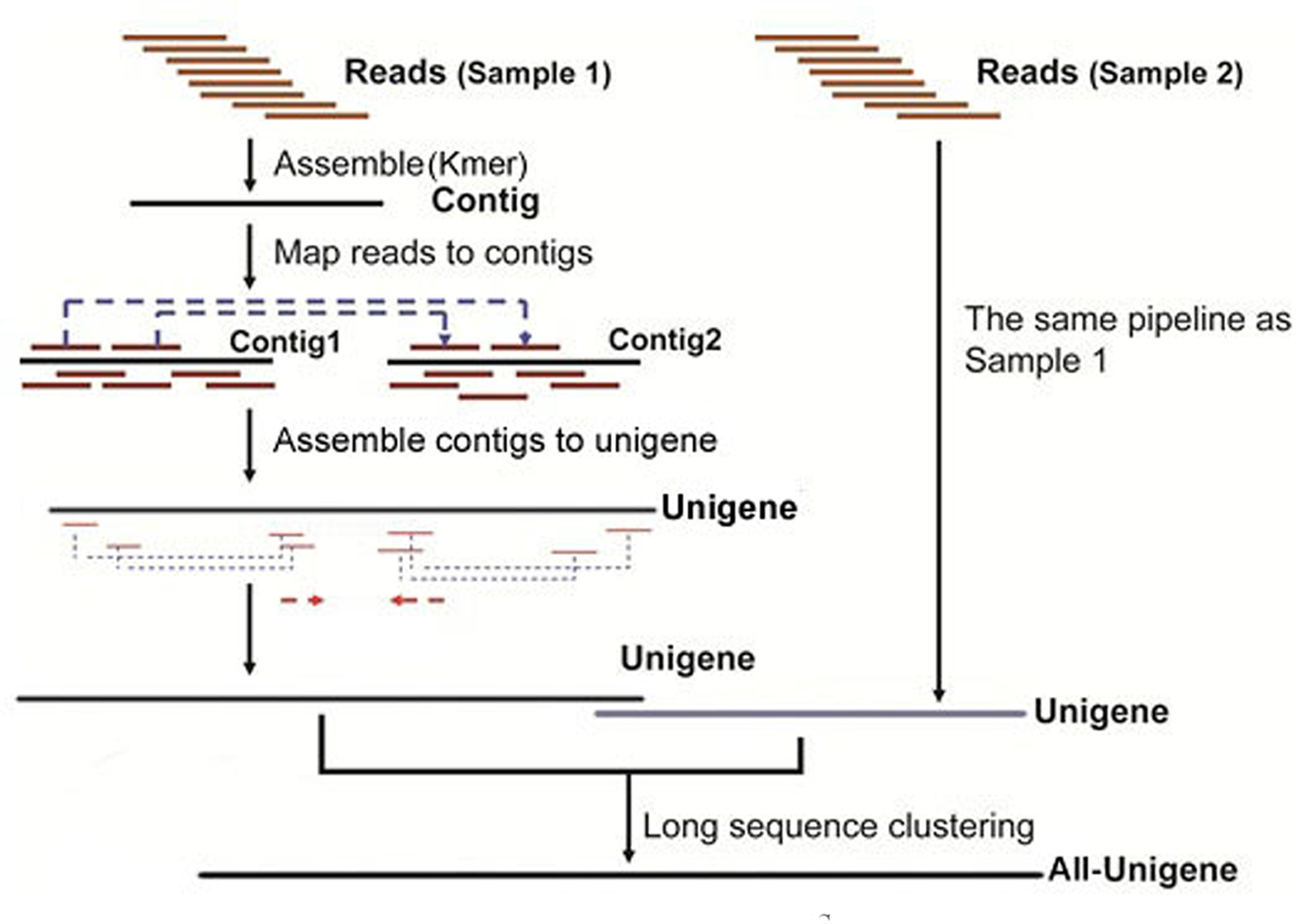

Supplement: Supplementary file 2 — Supplementary material 2 (JPEG 1233 kb) Fig. S2 Assembly process [file 438_2015_1112_MOESM2_ESM.jpg]

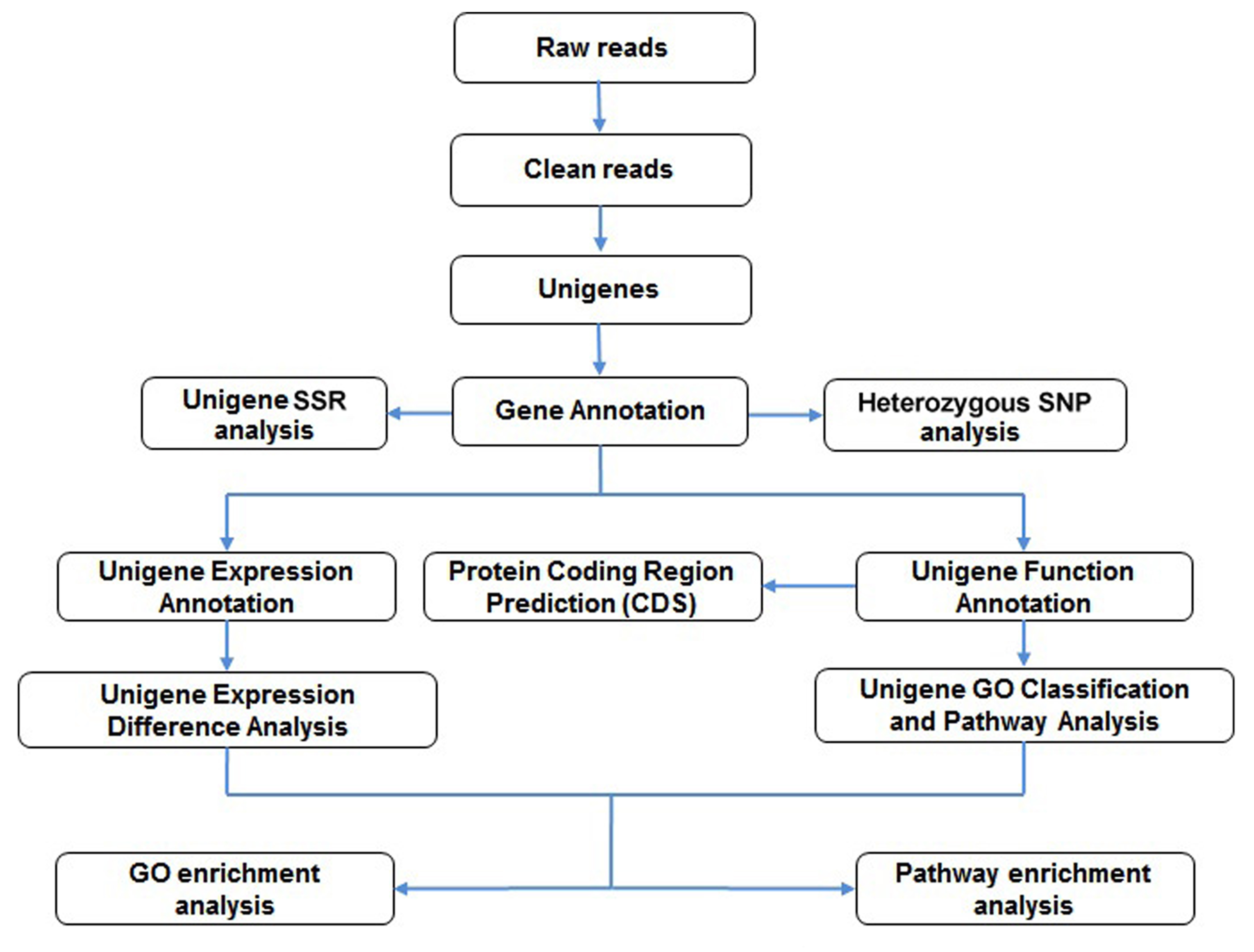

Supplement: Supplementary file 3 — Supplementary material 3 (JPEG 1428 kb) Fig. S3 Pipeline of bioinformatics analysis [file 438_2015_1112_MOESM3_ESM.jpg]

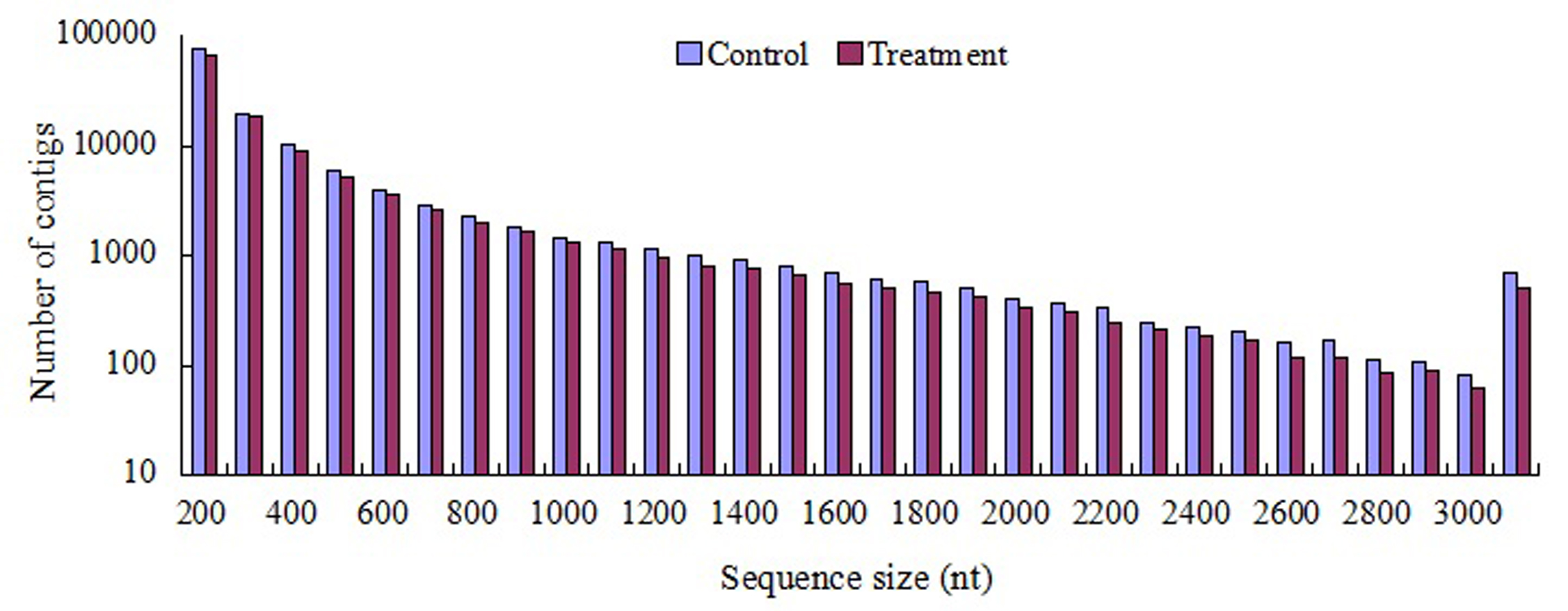

Supplement: Supplementary file 4 — Supplementary material 4 (JPEG 469 kb) Fig. S4 Length distribution of contigs from non-elicited and elicited samples [file 438_2015_1112_MOESM4_ESM.jpg]

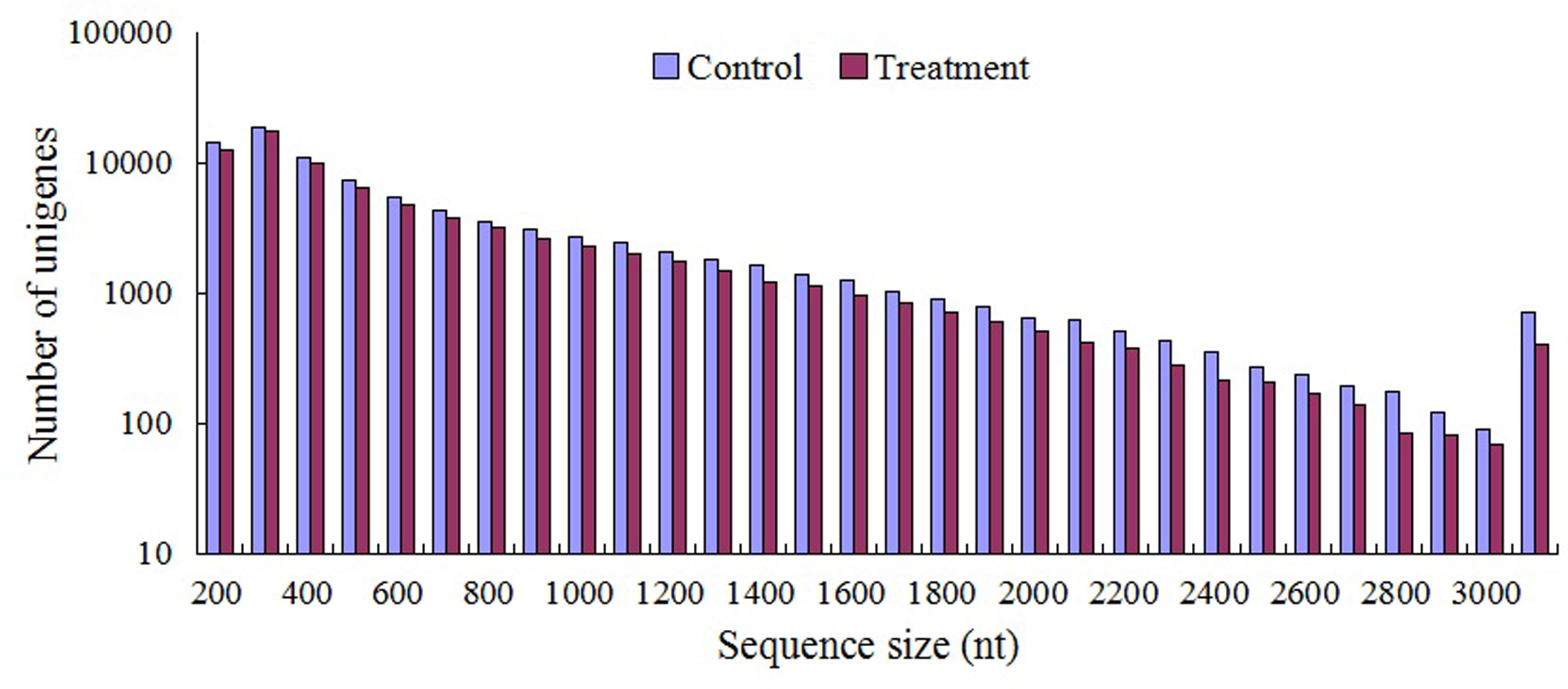

Supplement: Supplementary file 5 — Supplementary material 5 (JPEG 631 kb) Fig. S5 Length distribution of unigenes from non-elicited and elicited samples [file 438_2015_1112_MOESM5_ESM.jpg]

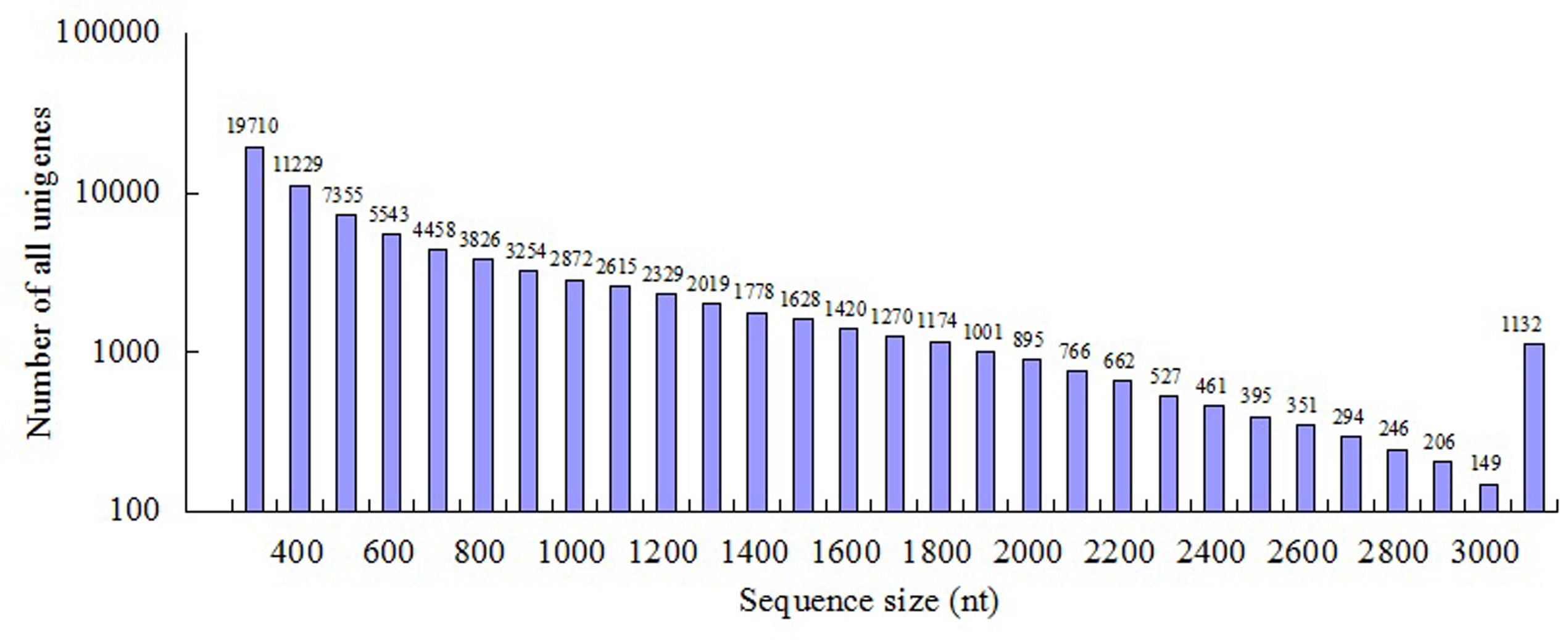

Supplement: Supplementary file 6 — Supplementary material 6 (JPEG 506 kb) Fig. S6 Length distribution of all assembled unigenes from non-elicited and elicited samples [file 438_2015_1112_MOESM6_ESM.jpg]

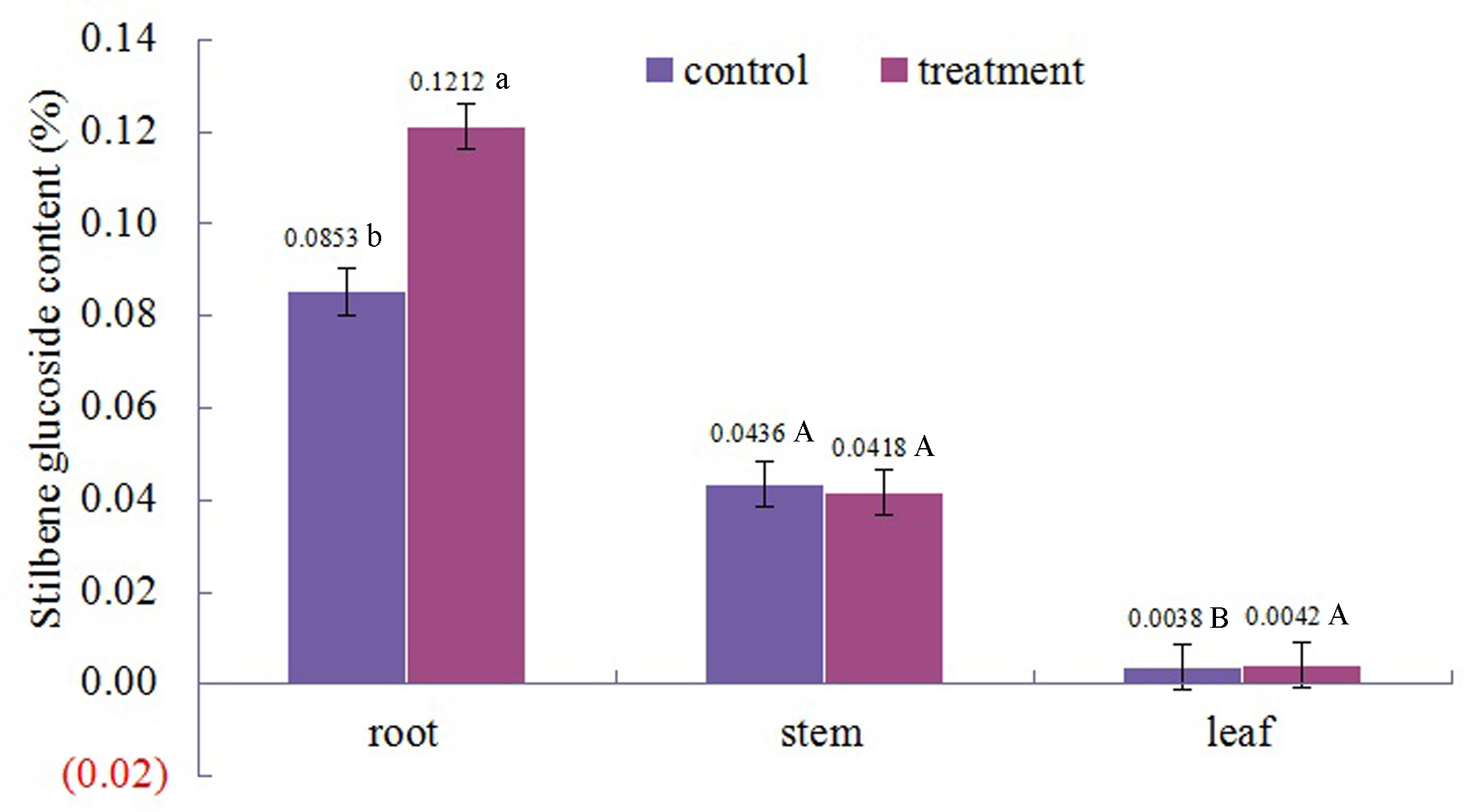

Supplement: Supplementary file 7 — Supplementary material 7 (JPEG 346 kb) Fig. S7 Effects of MeJA on stilbene glucoside content in P. multiflorum. [file 438_2015_1112_MOESM7_ESM.jpg]
