# Supplementary material for: Deep sequencing reveals transcriptome re-programming of Polygonum multiflorum thunb. roots to the elicitation with methyl jasmonate
Source: Mol Genet Genomics. 2015 Sep 5;291:337–48. doi: 10.1007/s00438-015-1112-9 (PMC4729805; doi:10.1007/s00438-015-1112-9)
Supplement: Supplementary file 8 — Supplementary material 8 (DOC 28 kb) [file 438_2015_1112_MOESM8_ESM.doc]

**Table S1** Description of the three reference genes (RG) and their amplified sequences. Raw sequences of reference genes and their PCR-amplified sequences were identical.

| **RG** | **Gene description** | **Homological species** | **Nr-ID** | **Original sequence CDS** | **Primer sequences (forward/reverse)** | **Amplicon sequences (bp)** | **Amplicon length (bp)** | **Identity(%)** |
| --- | --- | --- | --- | --- | --- | --- | --- | --- |
| *UBQ14* | Polyubiquitin 14 | Arabidopsis thaliana | gi|356548684|ref|XP_003542730.1| | ATGCAGATTTTTGTGAAGACCTTGACTGGAAAGACCATCACTTTGGAGGTTGAGAGTTCTGACACGATTGATAATGTTAAAGCCAAGATTCAGGACAAGGAGGGGATTCCCCCAGATCAGCAGCGTTTGATATTTGCTGGGAAGCAGTTGGAGGATGGCCGTACTCTTGCAGATTACAACATTCAAAAGGAGTCTACCCTTCACCTTGTGCTTCGTCTCCGTGGTGGG | AAGCCAAGATTCAGGACAAG/GGAGACGAAGCACAAGGT | AAGCCAAGATTCAGGACAAGGAGGGGATTCCCCCAGATCAGCAGCGTTTGATATTTGCTGGGAAGCAGTTGGAGGATGGCCGTACTCTTGCAGATTACAACATTCAAAAGGAGTCTACCCTTCACCTTGTGCTTCGTCTCC | 141 | 100.00 |
| *UBQ4-1* | Polyubiquitin 4 | Arabidopsis thaliana | gi|357148706|ref|XP_003574865.1| | GGCCGAACCCTAGCGGATTACAACATCCAAAAGGAGTCCACCCTCCATCTGGTGCTTCGTCTCAGAGGTGGTATGCAGATCTTCGTCAAAACCCTAACAGGCAAAACCATCACCCTTGAAGTCGAGAGCTCCGACACCATTGACAACGTTAAAGCTAAGATTCAAGACAAGGAGGGAATTCCCCCGGACCAGCAGAGGCTGATCTTCGCCGGCAAGCAATTGGAAGATGGAAGGACCCTTGCCGATTACAACATCCAGAAAGAATCAACTCTTCACTTGGTCCTCCGTCTTCGTGGAGGTATGCAGATTTTTGTCAAGACCCTCACCGGAAAAACCATTACTTTGGAAGTGGAGAGCTCAGACACCATTGACAATGTGAAGGCCAAGATCCAGGATAAGGAAGGGATTCCACCAGACCAACAGAGGCTGATTTTCGCCGGGAAGCAGCTTGAAGATGGAAGGACGTTGGCAGACTACAACATTCAAAAGGAGTCGACTCTCCACTTGGTCCTTCGTCTCCGTGGAGGT | CCGTCTTCGTGGAGGTAT/CTGTTGGTCTGGTGGAATC | CCGTCTTCGTGGAGGTATGCAGATTTTTGTCAAGACCTCACCGGAAAAACCATTACTTTGGAAGTGAGAGCTCAGACACCATTGACAATGTGAAGGCCAAGATCAGGATAAGGAAGGGATTCCACCAGACCAACAG | 139 | 100.00 |
| *SAMS* | S-adenosylmethionine synthase | Phaseolus lunatus | gi|75304713|sp|Q8W3Y4.1|METK_PHALU | ATGGACAACACCTTCTTGTTCACCTCAGAGTCAGTGAATGAGGGCCACCCCGACAAGCTCTGCGATCAGGTCTCTGATGCTGTGCTCGACGCTTGCCTGGAGCAGGATCCCGAGAGCAAAGTCGCTTGTGAGACTTGTGCCAAGACCAACCTAGTAATGGTGTTTGGTGAAATCACCACCAAGGCCAAAGTAAACTACGAAAAGATCGTTCGTGACACCTGCAGAAATATTGGCTTCGTCTCTCATGATGTTGGTCTTGATGCTGACAATTGCAAGGTCTTGGTCCACATTGATCAGCAAAGCCCTGATATTGCTCGGGGTGTCCATGGTCACAACACCAAACGCCCCGAGGAGATT | GCCAAGACCAACCTAGTAAT/AGCATCAAGACCAACATCAT | GCCAAGACCAACCTAGTAATGGTGTTTGGTGAAATCACCACCAAGGCCAAAGTAAACTACGAAAAGATCGTTCGTGACACCTGCAGAAATATTGGCTTCGTCTCTCATGATGTTGGTCTTGATGCT | 126 | 100.00 |
