# Supplementary material for: Deep sequencing reveals transcriptome re-programming of Polygonum multiflorum thunb. roots to the elicitation with methyl jasmonate
Source: Mol Genet Genomics. 2015 Sep 5;291:337–48. doi: 10.1007/s00438-015-1112-9 (PMC4729805; doi:10.1007/s00438-015-1112-9)
Supplement: Supplementary file 9 — Supplementary material 9 (DOC 158 kb) [file 438_2015_1112_MOESM9_ESM.doc]

**Table S2** Primers and annealing temperatures of genes of interest (GOIs). An annealing temperature of 52°C was selected as suitable for amplifying all GOIs based on their quantitations.

| **No.** | **GOI** | **Forward primer** | **Reverse primer** | **Ta(**℃) |
| --- | --- | --- | --- | --- |
| **1** | CL554.Contig1_All | ACCGTCTAGTCCGTGATG | CGATTCTCCATACCACAAGG | 52 |
| **2** | CL194.Contig1_All | CTTGGCGGAACAGATACTT | TCTTCTATCAGGCGGCTAT | 52 |
| **3** | CL194.Contig2_All | TCAGAGGAGCAGAGAAGAG | GAGAGCAGAACATCAATCATC | 52 |
| **4** | CL2377.Contig1_All | CCAAGACAATAGCGAGGAT | ATGAAGGAGTTCTGCCATC | 52 |
| **5** | CL2671.Contig3_All | GGTTGAGAAGAATAAGGATGG | TGGAGAGTGTGACATTAGAC | 52 |
| **6** | CL4001.Contig3_All | CTCATCCTCATCTTCCTCAC | GAAGTCGTTCTCTAGCATTG | 52 |
| **7** | CL4287.Contig2_All | CCTTGTTCTCCTCAATGTATG | AATGCTGTCCTCTGTAATCT | 52 |
| **8** | CL4799.Contig3_All | GGAGATTAGGAAGGCACAA | TTGGTGATGCGGAAGTAAT | 52 |
| **9** | CL5341.Contig4_All | GAGAGCGGTGGAATCATG | CTAAGGCGATGCTGATCC | 52 |
| **10** | CL5399.Contig4_All | AAGTTCAGTAGGAGCCAATG | ACTACAGCCTCAACAATTCC | 52 |
| **11** | CL5403.Contig3_All | TTGTTCTCGGAGTGTACTACT | GGATGCGGTGGATGATGT | 52 |
| **12** | CL556.Contig4_All | ACACGAACTCCAACATAGAC | CCTCCAAGAACACGACTG | 52 |
| **13** | CL8155.Contig1_All | ACCTTGTTCGTTACTCCATT | CGGCAGACTTGACAGAAT | 52 |
| **14** | CL8199.Contig1_All | TAGGCTACGGAACCAACT | TAGTGGCAGGAGGAGTAG | 52 |
| **15** | CL11757.Contig1_All | CAACAACCTCATCATCATCATC | GCACAGGACGGAGTAGTT | 52 |
| **16** | CL2466.Contig2_All | GCTCACAGGATCATCTACT | TTCTCAACACTCACTCAACT | 52 |
| **17** | CL2476.Contig1_All | GCCTTCCGTCTTCAGATT | GCTTATCACCTCGCAGTT | 52 |
| **18** | CL2476.Contig2_All | CATCTGCCAACCGTCTTC | CGTGATATGCTCCAAGTAGT | 52 |
| **19** | CL4508.Contig1_All | TTCCTCCTCCTCACAGTT | GTCGTTGCTGGAAGTAGT | 52 |
| **20** | CL5630.Contig4_All | CAGTGGCATAGGCAACTT | AGATGTGGTAGGAGGAGAG | 52 |
| **21** | CL7754.Contig1_All | AAGTATTGGTGCCGAAGG | ATCTGCTGAGGTGGTAGT | 52 |
| **22** | Unigene1793_All | GTTGGCGACGGAGATAAG | TCCTTCACCTTCTTCTTCAC | 52 |
| **23** | Unigene2635_All | GCCGTTGAGTGTAAGTGT | TCTCCTGGTGTCATCTCC | 52 |
| **24** | Unigene3242_All | ATGACACTCTTCCTGATGG | TCACTAAGCCACCGTTCT | 52 |
| **25** | Unigene4450_All | GGTGACAAGTATGAGAGTATGA | GCCTCCTCAACCACAGTT | 52 |
| **26** | Unigene6757_All | TACATTCACCGAGTTGGAG | CTTACGACCGAGGTTGAG | 52 |
| **27** | Unigene7583_All | CACTCTTACATTGCGTCTCT | GACTCCGTGGTGTTGATAG | 52 |
| **28** | Unigene8987_All | TGGAAGCGGTTAGAGATG | TGCCAATCCTACTCAACTC | 52 |
| **29** | Unigene10228_All | GTCTGAACAAGTGGAGGAG | CCTACTAGCCAGCCTAAGA | 52 |
| **30** | Unigene12763_All | GCCAGCATCACAACAATG | CCATTCTTACAGCACTCCT | 52 |
| **31** | Unigene12765_All | CATCACAGGAGTTCTTGGAT | CCGCATCACGCCTTATAC | 52 |
| **32** | Unigene14565_All | AGTGGTGGACAACAAGTTC | GGACAGGAGGAAGCAAGT | 52 |
| **33** | Unigene14931_All | CCTCGTCATCATCGTTCC | AAGTTGAGGCGGTGTATG | 52 |
| **34** | Unigene15235_All | GTCCAGTCCTTCTCTTCAAT | TGGCATTATCAACCTCTTCC | 52 |
| **35** | Unigene15442_All | CCGTCTCTTCCACTCTTC | ACGAGGAGGAGATTATGATG | 52 |
| **36** | Unigene17080_All | TCACCACCGCTAATCCTT | ATCATCTCCAACGCTAAGTC | 52 |
| **37** | Unigene17201_All | ATGCGAAGTGTTCCGATT | CATCATTATCCGTGACACAT | 52 |
| **38** | Unigene18031_All | GCTTAACGAACGGATACCT | CGTCGGTGGTTCTAATGAT | 52 |
| **39** | Unigene18687_All | CCTCATCCTCAACAACCTAGCC | CCATCGGCGTGCAGAACAT | 52 |
| **40** | Unigene19181_All | ACTCGCATTCAATCCAACT | CATTAGTGGTGTGGTAGTGT | 52 |
| **41** | Unigene19630_All | CTGACACGAACTCCAACAT | CACTACTATCTCTACACGAACT | 52 |
| **42** | Unigene19863_All | ATGAGGAAGAAGTCGCTTG | CTTCGTAGCACCACAGTT | 52 |
| **43** | Unigene20977_All | CAACATCAACCATCGTCAT | AGAATTGCTGTAACCTTGTC | 52 |
| **44** | Unigene24860_All | GGTGAAGGACTCGCTATG | GCACATTCCTGTAGATCAAC | 52 |
| **45** | Unigene25824_All | GAGACTTCCTGCCATTGAT | CTTGACCTGAATCCGTTAATC | 52 |
| **46** | Unigene25987_All | CCCATCTCTGCGTTACATTCTC | GACATCCTTGCCTCTGTCTATC | 52 |
| **47** | Unigene39885_All | ACCGCCTCAACCTCCTATCC | TCCTCCGATTCCACGCTCTC | 52 |
| **48** | Unigene40223_All | GCCAGCATCACAACAATG | CCATTCTTACAGCACTCCT | 52 |
| **49** | Unigene43261_All | TAGGAGGAGGAACGGTCT | AGGCTAAGAACCAACCAAG | 52 |
| **50** | Unigene43262_All | CGTGGTTGTTACATCAGTTG | GAATGCTCGTGGTGGTTC | 52 |
| **51** | CL2465.Contig3_All | GGACCACTGGCGTAAGAT | CTGACATCCTCCACAACAC | 52 |
| **52** | CL422.Contig1_All | GTCACTACAACTCGGCACCAT | TCAGCATCCTCCACCTCTCG | 52 |
| **53** | CL1442.Contig1_All | ACCTTCGTTGCCTTCGCTTG | CAGGAGAGGATGGAGGAGATGT | 52 |
| **54** | CL1442.Contig3_All | TGGCTTGGTCTGGCTCTACTAC | ATGTGGGCGAAGGCGAAGT | 52 |
| **55** | CL1491.Contig3_All | ACTGGCGAACCCTAAGAAG | CCTAAGCGTTTCGGTGAGAT | 52 |
| **56** | CL1491.Contig4_All | CGTCCAGTGAAAGAGTCAGC | GCCGTGATCTCCCTACAAC | 52 |
| **57** | CL1912.Contig1_All | GTGAGGTTGGTGGTGTTG | CGGCTCTTAGGCATAGTAAT | 52 |
| **58** | CL2342.Contig9_All | CGAATCCGTAGTTCCTCAT | CGAGTGGTGGTTATGGTT | 52 |
| **59** | CL2377.Contig3_All | CCGTGACAAGAGCGAGGATT | GGATGGTTGGTTGACGAAGAAG | 52 |
| **60** | CL4044.Contig3_All | ATCTCACCTACCTCCATTCT | GAAGGAGTGTAAGATGATGATG | 52 |
| **61** | CL5221.Contig2_All | CCCTCCCTTCTTCGCCTTCT | CGGAGACGATGACGGTGATG | 52 |
| **62** | CL7434.Contig1_All | TTCTATGGCAGCGGCTCATTG | CGGTTGGAGGTTGTGGAAGAG | 52 |
| **63** | CL10212.Contig1_All | TGTCCAGTGTGCCCTTTCAAG | AACGACTCCAATGCCGCTAAG | 52 |
| **64** | CL2342.Contig16_All | TTCTGAACGCTGGAATCTT | TTGGAGGAGAGTGTTGATG | 52 |
| **65** | Unigene6737_All | CCGTTCTTGAGAGGTTATCT | TATTGCTTGATGGCTTGGT | 52 |
| **66** | Unigene7711_All | GCCGCTTCCACCTCTTCTCT | TGAGGCAGTAGGAACCGCTAG | 52 |
| **67** | Unigene19492_All | CGGTGTTGGAAGGAGGAGTT | GGCAGCGTCATCTCAAAGTTCT | 52 |
| **68** | Unigene20299_All | TTCAGAGGCACAGACACAGTTG | AACAATGGCGGTGAGGTATGG | 52 |
| **69** | Unigene23516_All | TCGGTGGAGGAGAAGAGCAT | CGGAGTTGAGAAGAGACGGTTC | 52 |
| **70** | Unigene26381_All | CTCAATCATCACCTACATTCC | CGAAGTTGTTGGCTTGAAT | 52 |
