# Supplementary material for: Deep sequencing reveals transcriptome re-programming of Polygonum multiflorum thunb. roots to the elicitation with methyl jasmonate
Source: Mol Genet Genomics. 2015 Sep 5;291:337–48. doi: 10.1007/s00438-015-1112-9 (PMC4729805; doi:10.1007/s00438-015-1112-9)
Supplement: Supplementary file 10 — Supplementary material 10 (DOC 248 kb) [file 438_2015_1112_MOESM10_ESM.doc]

**Table S3** Determination of up- and down-regulation of genes of interest (GOIs) based on transcriptome sequencing. Unigene expression was calculated using the fragments per kb per million mapped fragments (FPKM) method according to the formula *FPKM* = 10^6^ *C* / [(*NL*)/ 10^3^], where *FPKM* is the expression of unigene A, *C* is the number of fragments uniquely aligned to unigene A, *N* is the total number of fragments uniquely aligned to all unigenes, and *L* is the number of bases in the coding sequence of unigene A.

| **No.** | **GOI** | **Gene length** | **Control_FPKM** | **Treatment_FPKM** | **log2(treatment_FPKM/control_FPKM)** | **up/down** | **pvalue** | **FDR** |
| --- | --- | --- | --- | --- | --- | --- | --- | --- |
| **1** | CL554.Contig1_All | 1834 | 32.7005 | 66.3137 | 1.02 | Up | 8.81E-117 | 4.19E-115 |
| **2** | CL194.Contig1_All | 1784 | 3.7921 | 24.0905 | 2.6674 | Up | 3.29E-159 | 2.05E-157 |
| **3** | CL194.Contig2_All | 1773 | 5.4776 | 28.6292 | 2.3859 | Up | 1.97E-165 | 1.28E-163 |
| **4** | CL2377.Contig1_All | 1587 | 1.4122 | 6.4807 | 2.1982 | Up | 1.06E-31 | 1.59E-30 |
| **5** | CL2671.Contig3_All | 1839 | 93.9756 | 219.5588 | 1.2242 | Up | 0 | 0 |
| **6** | CL4001.Contig3_All | 1793 | 5.1388 | 161.7018 | 4.9758 | Up | 0 | 0 |
| **7** | CL4287.Contig2_All | 1915 | 62.5264 | 251.6665 | 2.009 | Up | 0 | 0 |
| **8** | CL4799.Contig3_All | 390 | 3.4054 | 17.5475 | 2.3654 | Up | 1.84E-23 | 2.16E-22 |
| **9** | CL5341.Contig4_All | 770 | 0.3773 | 1.7775 | 2.2361 | Up | 0.0000211 | 0.0000727 |
| **10** | CL5399.Contig4_All | 860 | 2.2682 | 4.911 | 1.1145 | Up | 0.00000464 | 0.0000176 |
| **11** | CL5403.Contig3_All | 689 | 1.9276 | 5.4487 | 1.4991 | Up | 5.71E-08 | 0.000000272 |
| **12** | CL556.Contig4_All | 1971 | 8.5281 | 54.7007 | 2.6813 | Up | 0 | 0 |
| **13** | CL8155.Contig1_All | 1505 | 15.3053 | 43.9649 | 1.5223 | Up | 9.41E-119 | 4.55E-117 |
| **14** | CL8199.Contig1_All | 1590 | 40.7989 | 193.8817 | 2.2486 | Up | 0 | 0 |
| **15** | CL11757.Contig1_All | 1879 | 175.3358 | 448.5006 | 1.355 | Up | 0 | 0 |
| **16** | CL2466.Contig2_All | 983 | 5.3199 | 17.902 | 1.7506 | Up | 9.31E-40 | 1.7E-38 |
| **17** | CL2476.Contig1_All | 2113 | 46.8266 | 94.9424 | 1.0197 | Up | 2.91E-191 | 2.16E-189 |
| **18** | CL2476.Contig2_All | 425 | 2.832 | 9.6615 | 1.7704 | Up | 1.58E-10 | 9.49E-10 |
| **19** | CL4508.Contig1_All | 1554 | 7.2645 | 29.4175 | 2.0177 | Up | 2.2E-121 | 1.08E-119 |
| **20** | CL5630.Contig4_All | 1760 | 36.9996 | 190.2859 | 2.3626 | Up | 0 | 0 |
| **21** | CL7754.Contig1_All | 1813 | 238.7203 | 764.992 | 1.6801 | Up | 0 | 0 |
| **22** | Unigene1793_All | 630 | 56.3264 | 267.6583 | 2.2485 | Up | 0 | 0 |
| **23** | Unigene2635_All | 2733 | 7.6082 | 23.5236 | 1.6285 | Up | 4.02E-127 | 2.06E-125 |
| **24** | Unigene3242_All | 1652 | 4.0197 | 8.0721 | 1.0059 | Up | 5.91E-14 | 4.49E-13 |
| **25** | Unigene4450_All | 1436 | 28.7288 | 66.1751 | 1.2038 | Up | 3.75E-119 | 1.83E-117 |
| **26** | Unigene6757_All | 969 | 19.1885 | 192.8257 | 3.329 | Up | 0 | 0 |
| **27** | Unigene7583_All | 1125 | 2.7669 | 6.2917 | 1.1852 | Up | 4.37E-10 | 2.53E-09 |
| **28** | Unigene8987_All | 201 | 2.8908 | 12.0625 | 2.061 | Up | 5.97E-08 | 0.000000283 |
| **29** | Unigene10228_All | 1684 | 1.8238 | 5.9216 | 1.699 | Up | 2.54E-22 | 2.85E-21 |
| **30** | Unigene12763_All | 910 | 1.8243 | 23.6784 | 3.6982 | Up | 1.06E-109 | 4.72E-108 |
| **31** | Unigene12765_All | 910 | 1.4139 | 39.9654 | 4.821 | Up | 3.32E-220 | 2.85E-218 |
| **32** | Unigene14565_All | 1619 | 10.0234 | 20.4346 | 1.0276 | Up | 1.94E-33 | 3.04E-32 |
| **33** | Unigene14931_All | 710 | 2.1044 | 4.902 | 1.22 | Up | 0.00000806 | 0.0000296 |
| **34** | Unigene15235_All | 1246 | 4.7966 | 11.8008 | 1.2988 | Up | 3.78E-22 | 4.23E-21 |
| **35** | Unigene15442_All | 1741 | 2.6223 | 10.6918 | 2.0276 | Up | 9.04E-51 | 2.04E-49 |
| **36** | Unigene17080_All | 2182 | 34.58 | 103.697 | 1.5844 | Up | 0 | 0 |
| **37** | Unigene17201_All | 360 | 53.3783 | 576.2692 | 3.4324 | Up | 0 | 0 |
| **38** | Unigene18031_All | 1475 | 6.6124 | 13.8925 | 1.0711 | Up | 1.06E-22 | 1.21E-21 |
| **39** | Unigene18687_All | 393 | 1.4785 | 4.8758 | 1.7215 | Up | 0.0000205 | 0.0000706 |
| **40** | Unigene19181_All | 320 | 2.594 | 15.398 | 2.5695 | Up | 7.15E-19 | 6.96E-18 |
| **41** | Unigene19630_All | 1792 | 6.5544 | 41.6155 | 2.6666 | Up | 8.89E-275 | 9.41E-273 |
| **42** | Unigene19863_All | 363 | 2.058 | 27.0402 | 3.7158 | Up | 5.44E-51 | 1.23E-49 |
| **43** | Unigene20977_All | 420 | 1.1858 | 15.5493 | 3.7129 | Up | 2.24E-34 | 3.6E-33 |
| **44** | Unigene24860_All | 478 | 1.997 | 10.3083 | 2.3679 | Up | 2.62E-17 | 2.38E-16 |
| **45** | Unigene25824_All | 1764 | 3.0351 | 9.1779 | 1.5964 | Up | 2.08E-32 | 3.18E-31 |
| **46** | Unigene25987_All | 534 | 0.3109 | 2.0505 | 2.7215 | Up | 0.0000229 | 0.0000783 |
| **47** | Unigene39885_All | 1724 | 3.563 | 10.0714 | 1.4991 | Up | 9.59E-32 | 1.44E-30 |
| **48** | Unigene40223_All | 882 | 1.8823 | 6.828 | 1.859 | Up | 1.02E-15 | 8.48E-15 |
| **49** | Unigene43261_All | 697 | 0.8932 | 8.0232 | 3.1671 | Up | 5.4E-26 | 6.88E-25 |
| **50** | Unigene43262_All | 420 | 1.087 | 15.363 | 3.821 | Up | 1.02E-34 | 1.65E-33 |
| **51** | CL2465.Contig3_All | 1135 | 193.1108 | 50.9927 | -1.9211 | Down | 0 | 0 |
| **52** | CL422.Contig1_All | 1898 | 8.7905 | 2.1634 | -2.0226 | Down | 4.05E-46 | 8.4E-45 |
| **53** | CL1442.Contig1_All | 1793 | 190.5279 | 87.5467 | -1.1219 | Down | 0 | 0 |
| **54** | CL1442.Contig3_All | 1019 | 17.8804 | 5.91 | -1.5971 | Down | 1.14E-36 | 1.93E-35 |
| **55** | CL1491.Contig3_All | 1833 | 16.3026 | 3.0081 | -2.4382 | Down | 4E-102 | 1.68E-100 |
| **56** | CL1491.Contig4_All | 1785 | 14.1601 | 5.4113 | -1.3878 | Down | 4.63E-41 | 8.69E-40 |
| **57** | CL1912.Contig1_All | 1748 | 23.7435 | 3.9151 | -2.6004 | Down | 6.54E-152 | 3.89E-150 |
| **58** | CL2342.Contig9_All | 2414 | 26.8897 | 24.8988 | -0.111 | Down | 0.032197 | 0.054483 |
| **59** | CL2377.Contig3_All | 1024 | 7.255 | 0.4965 | -3.8691 | Down | 5.97E-41 | 1.12E-39 |
| **60** | CL4044.Contig3_All | 1001 | 4.1462 | 1.4845 | -1.4818 | Down | 1.23E-08 | 6.29E-08 |
| **61** | CL5221.Contig2_All | 2061 | 6.0413 | 2.3528 | -1.3605 | Down | 1.85E-20 | 1.93E-19 |
| **62** | CL7434.Contig1_All | 1908 | 14.8352 | 6.7021 | -1.1463 | Down | 2.25E-34 | 3.61E-33 |
| **63** | CL10212.Contig1_All | 1997 | 14.2156 | 5.0522 | -1.4925 | Down | 5.05E-51 | 1.14E-49 |
| **64** | CL2342.Contig16_All | 2712 | 72.1416 | 62.4801 | -0.2074 | Down | 8.148E-12 | 5.4021E-11 |
| **65** | Unigene6737_All | 830 | 97.4585 | 47.7752 | -1.0285 | Down | 6.62E-80 | 2.23E-78 |
| **66** | Unigene7711_All | 1945 | 342.5277 | 158.2935 | -1.1136 | Down | 0 | 0 |
| **67** | Unigene19492_All | 526 | 103.6013 | 46.1688 | -1.1661 | Down | 2.56E-66 | 7.34E-65 |
| **68** | Unigene20299_All | 849 | 2.2487 | 0.4146 | -2.4393 | Down | 0.00000008 | 0.000000374 |
| **69** | Unigene23516_All | 1965 | 8.6809 | 3.8011 | -1.1914 | Down | 1.3E-22 | 1.48E-21 |
| **70** | Unigene26381_All | 780 | 9.897 | 4.9133 | -1.0103 | Down | 8.77E-09 | 4.54E-08 |
