# Supplementary material for: Deep sequencing reveals transcriptome re-programming of Polygonum multiflorum thunb. roots to the elicitation with methyl jasmonate
Source: Mol Genet Genomics. 2015 Sep 5;291:337–48. doi: 10.1007/s00438-015-1112-9 (PMC4729805; doi:10.1007/s00438-015-1112-9)
Supplement: Supplementary file 11 — Supplementary material 11 (DOC 179 kb) [file 438_2015_1112_MOESM11_ESM.doc]

**Table S4** statistics of unigenes with the relatively high abundance of more than 1000 transcripts per million (relative abundance) in the non-elicited and elicited samples

| No. | geneID | geneLength | control_FPKM | treatment_FPKM | Nr-Score | Nr-Evalue | Nr-annotation |
| --- | --- | --- | --- | --- | --- | --- | --- |
| 1 | CL55.Contig8_All | 5285 | 21094.7485 | 27510.5817 | 66.6 | 4.00E-09 | hypothetical protein MTR_5g051130 [Medicago truncatula] >gi|355515729|  gb|AES97352.1| hypothetical protein MTR_5g051130 [Medicago truncatula] |
| 2 | CL2591.Contig4_All | 1673 | 20558.7145 | 24468.5249 | 721.5 | 0 | hypothetical protein MTR_5g050970 [Medicago truncatula] >gi|355515717|  gb|AES97340.1| hypothetical protein MTR_5g050970 [Medicago truncatula] |
| 3 | CL384.Contig4_All | 372 | 2384.5626 | 2223.2556 | 70.5 | 7.00E-12 | unnamed protein product [Vitis vinifera] |
| 4 | CL6145.Contig1_All | 1268 | 2361.616 | 4938.171 | 342.8 | 6.00E-93 | hypothetical protein VITISV_043424 [Vitis vinifera] |
| 5 | CL6145.Contig2_All | 1135 | 2215.8555 | 4628.2114 | 342.8 | 5.00E-93 | hypothetical protein VITISV_043424 [Vitis vinifera] |
| 6 | CL4602.Contig1_All | 389 | 1940.8552 | 3216.9394 | 55.8 | 2.00E-07 | major latex protein homolog [Mesembryanthemum crystallinum] |
| 7 | Unigene1663_All | 1002 | 1932.653 | 1886.4118 | 108.6 | 1.00E-22 | hypothetical protein [Trifolium pratense] |
| 8 | CL384.Contig2_All | 789 | 1500.0225 | 1262.4422 | 295.4 | 5.00E-79 | PREDICTED: polyubiquitin 10-like [Glycine max] |
| 9 | Unigene24500_All | 1017 | 1448.9545 | 1142.9157 | 220.7 | 2.00E-56 | lectin [Limonium bicolor] |
| 10 | CL2382.Contig1_All | 423 | 1172.0117 | 1967.5917 | 141.4 | 3.00E-33 | major latex protein homolog [Mesembryanthemum crystallinum] |
| 11 | Unigene12580_All | 387 | 3790.2367 | 607.4049 | 76.3 | 1.00E-13 | extensin [Solanum tuberosum] |
| 12 | Unigene25200_All | 440 | 2000.8527 | 373.106 | 59.7 | 1.00E-08 | extensin precursor [Nicotiana plumbaginifolia] |
| 13 | Unigene14611_All | 1378 | 1842.3038 | 387.3416 | 225.7 | 1.00E-57 | PREDICTED: dehydration-responsive protein RD22-like isoform 2 [Vitis vinifera] |
| 14 | Unigene5766_All | 1146 | 1425.0669 | 627.8786 | 222.2 | 1.00E-56 | Phosphoprotein ECPP44, putative [Ricinus communis] >gi|223551337|gb|EEF  52823.1| Phosphoprotein ECPP44, putative [Ricinus communis] |
| 15 | CL9234.Contig1_All | 441 | 1365.0086 | 79.7193 | 53.1 | 1.00E-06 | PREDICTED: proline-rich 33 kDa extensin-related protein-like [Vitis vinifera] |
| 16 | CL7637.Contig2_All | 200 | 1226.4325 | 211.563 | 56.2 | 1.00E-07 | hypothetical protein MTR_056s0017 [Medicago truncatula] >gi|355502699|  gb|AES83902.1| hypothetical protein MTR_056s0017 [Medicago truncatula] |
| 17 | CL384.Contig3_All | 315 | 1140.4936 | 898.9396 | 150.6 | 5.00E-36 | PREDICTED: polyubiquitin-like [Brachypodium distachyon] |
| 18 | CL6722.Contig1_All | 1065 | 1135.6019 | 786.1208 | 291.2 | 1.00E-77 | pathogenesis-related protein 10a [Rheum australe] |
| 19 | Unigene2370_All | 565 | 1124.2711 | 885.8702 | 287.3 | 7.00E-77 | isocitrate lyase, putative [Ricinus communis] >gi|223530782|gb|EEF32648.1| isocitrate lyase, putative [Ricinus communis] |
| 20 | Unigene24547_All | 732 | 1063.1055 | 238.2149 | 80.5 | 2.00E-14 | unnamed protein product [Vitis vinifera] |
| 21 | CL10288.Contig1_All | 566 | 1053.1365 | 549.5556 | 139 | 3.00E-32 | metallothionein [Vitis vinifera] >gi|161778790|gb|ABX79346.1| metallothionein [Vitis vinifera] |
| 22 | Unigene7884_All | 1861 | 1036.8996 | 626.5773 | 860.5 | 0 | hexose transporter [Vitis vinifera] |
| 23 | Unigene18207_All | 748 | 1030.7662 | 888.8755 | 343.2 | 2.00E-93 | PREDICTED: polyubiquitin-like [Brachypodium distachyon] |
| 24 | CL9234.Contig2_All | 640 | 1030.5872 | 143.8365 | 70.5 | 2.00E-11 | PREDICTED: uncharacterized protein LOC100233119 [Vitis vinifera] >gi  |35187383|gb|AAQ84302.1| putative ripening-induced protein 1 precursor [Vitis vinifera] |
| 25 | CL4785.Contig1_All | 1728 | 1026.9268 | 937.9989 | 267.7 | 3.00E-70 | basic 7S globulin 2 precursor small subunit, putative [Ricinus communis] >gi  |223543800|gb|EEF45328.1| basic 7S globulin 2 precursor small subunit, putative [Ricinus communis] |
| 26 | CL1228.Contig3_All | 468 | 860.225 | 4062.0021 | 87.4 | 7.00E-17 | PREDICTED: uncharacterized protein LOC100832808 [Brachypodium distachyon] |
| 27 | CL6999.Contig2_All | 1218 | 877.744 | 3109.0812 | 300.1 | 4.00E-80 | endo-1,3-1,4-beta-d-glucanase, putative [Ricinus communis] >gi|223543880|  gb|EEF45406.1| endo-1,3-1,4-beta-d-glucanase, putative [Ricinus communis] |
| 28 | CL7627.Contig1_All | 513 | 455.0029 | 2916.5546 | -- | -- | -- |
| 29 | CL1228.Contig5_All | 1861 | 512.6959 | 2746.1139 | 205.3 | 2.00E-51 | conserved hypothetical protein [Ricinus communis] >gi|223541415|gb|EEF  42966.1| conserved hypothetical protein [Ricinus communis] |
| 30 | CL6999.Contig1_All | 1111 | 752.2208 | 2537.6961 | 300.1 | 3.00E-80 | endo-1,3-1,4-beta-d-glucanase, putative [Ricinus communis] >gi|223543880  |gb|EEF45406.1| endo-1,3-1,4-beta-d-glucanase, putative [Ricinus communis] |
| 31 | CL11564.Contig1_All | 1516 | 752.7325 | 2479.8983 | 422.9 | 6.00E-117 | hypothetical protein VITISV_042886 [Vitis vinifera] |
| 32 | CL1228.Contig9_All | 523 | 371.6282 | 2310.2402 | 100.9 | 8.00E-21 | unnamed protein product [Vitis vinifera] |
| 33 | Unigene1714_All | 1115 | 723.2056 | 1649.1475 | 252.3 | 9.00E-66 | LEA [Knorringia sibirica] |
| 34 | Unigene24734_All | 1082 | 452.7809 | 1533.1545 | 434.9 | 9.00E-121 | harpin-induced protein [Rheum australe] |
| 35 | CL1228.Contig6_All | 1952 | 297.8609 | 1481.4573 | 204.9 | 3.00E-51 | conserved hypothetical protein [Ricinus communis] >gi|223541415|gb|EEF  42966.1| conserved hypothetical protein [Ricinus communis] |
| 36 | Unigene40468_All | 3540 | 770.2091 | 1405.2834 | 35.8 | 1.00E-16 | Mitochondrial protein, putative [Medicago truncatula] >gi|355477403|gb|AES  58606.1| Mitochondrial protein, putative [Medicago truncatula] |
| 37 | Unigene22181_All | 1167 | 318.8347 | 1293.0421 | 201.4 | 2.00E-50 | PREDICTED: allene oxide cyclase 4, chloroplastic [Vitis vinifera] >gi|297737  859|emb|CBI27060.3| unnamed protein product [Vitis vinifera] |
| 38 | Unigene15398_All | 3726 | 716.5114 | 1288.5117 | 32.7 | 8.00E-72 | Mitochondrial protein, putative [Medicago truncatula] >gi|355477403|gb|AES  58606.1| Mitochondrial protein, putative [Medicago truncatula] |
| 39 | Unigene25434_All | 3606 | 702.1205 | 1279.7483 | 32.7 | 8.00E-72 | Mitochondrial protein, putative [Medicago truncatula] >gi|355477403|gb|AES  58606.1| Mitochondrial protein, putative [Medicago truncatula] |
| 40 | CL8953.Contig1_All | 1536 | 138.8318 | 1128.0378 | 779.2 | 0 | type III polyketide synthase 3 [Polygonum cuspidatum] |
| 41 | Unigene1670_All | 912 | 279.1029 | 1114.3901 | 168.7 | 1.00E-40 | nucleic acid binding protein, putative [Ricinus communis] >gi|223532145|gb|  EEF33952.1| nucleic acid binding protein, putative [Ricinus communis] |
| 42 | Unigene39916_All | 923 | 203.9209 | 1056.3259 | 101.7 | 1.00E-20 | hypothetical protein VITISV_020655 [Vitis vinifera] |
| 43 | CL9422.Contig2_All | 908 | 156.6442 | 1052.0268 | 192.6 | 6.00E-48 | tumor-related protein [Vitis cinerea var. helleri x Vitis riparia] |
| 44 | Unigene17168_All | 1282 | 512.2559 | 1034.8732 | 296.6 | 5.00E-79 | polygalacturonase-inhibiting protein [Knorringia sibirica] |
