# Supplementary material for: Deep sequencing reveals transcriptome re-programming of Polygonum multiflorum thunb. roots to the elicitation with methyl jasmonate
Source: Mol Genet Genomics. 2015 Sep 5;291:337–48. doi: 10.1007/s00438-015-1112-9 (PMC4729805; doi:10.1007/s00438-015-1112-9)
Supplement: Supplementary file 12 — Supplementary material 12 (DOC 255 kb) [file 438_2015_1112_MOESM12_ESM.doc]

**Table S5** Significantly modulated pathways in *Polygonum multiflorum* roots subjected to MeJA treatment. According to the results presented in this table, only a small fraction of genes (although still a large number) showed altered expression in the presence of MeJA.

| **No.** | **pathway** | **Up-regulated genes** | **Down-regulated genes** | **DEGs genes with pathway annotation (8936)** | **All genes with pathway annotation (32790)** |
| --- | --- | --- | --- | --- | --- |
| 1 | Glycolysis/gluconeogenesis | 25 | 74 | 99 (1.11%) | 460 (1.4%) |
| 2 | Citrate cycle (TCA cycle) | 7 | 20 | 27 (0.3%) | 151 (0.46%) |
| 3 | Pentose phosphate pathway | 19 | 34 | 53 (0.59%) | 171 (0.52%) |
| 4 | Pentose and Glucuronate interconversions | 58 | 145 | 203 (2.27%) | 457 (1.39%) |
| 5 | Fructose and mannose metabolism | 7 | 45 | 52 (0.58%) | 217 (0.66%) |
| 6 | Galactose metabolism | 9 | 62 | 71 (0.79%) | 284 (0.87%) |
| 7 | Ascorbate and aldarate metabolism | 25 | 59 | 84 (0.94%) | 189 (0.58%) |
| 8 | Fatty acid biosynthesis | 7 | 13 | 20 (0.22%) | 77 (0.23%) |
| 9 | Fatty acid elongation | 8 | 9 | 17 (0.19%) | 59 (0.18%) |
| 10 | Fatty acid metabolism | 8 | 40 | 48 (0.54%) | 181 (0.55%) |
| 11 | Synthesis and degradation of ketone bodies | / | 4 | 4 (0.04%) | 29 (0.09%) |
| 12 | Cutin, Suberine and Wax biosynthesis | 19 | 18 | 37 (0.41%) | 115 (0.35%) |
| 13 | Steroid biosynthesis | 1 | 30 | 31 (0.35%) | 111 (0.34%) |
| 14 | Ubiquinone and other terpenoid-quinone biosynthesis | 6 | 18 | 24 (0.27%) | 93 (0.28%) |
| 15 | Oxidative phosphorylation | 84 | 34 | 118 (1.32%) | 609 (1.86%) |
| 16 | Photosynthesis | 4 | 12 | 16 (0.18%) | 114 (0.35%) |
| 17 | Photosynthesis-antenna proteins | 4 | 1 | 5 (0.06%) | 24 (0.07%) |
| 18 | Purine metabolism | 59 | 202 | 261 (2.92%) | 919 (2.8%) |
| 19 | Pyrimidine metabolism | 52 | 205 | 257 (2.88%) | 895 (2.73%) |
| 20 | Alanine, Aspartate and glutamate metabolism | 3 | 34 | 37 (0.41%) | 208 (0.63%) |
| 21 | Glycine, serine and threonine metabolism | 10 | 44 | 54 (0.6%) | 167 (0.51%) |
| 22 | Cysteine and methionine metabolism | 28 | 64 | 92 (1.03%) | 290 (0.88%) |
| 23 | Valine, leucine and isoleucine degradation | 5 | 50 | 55 (0.62%) | 174 (0.53%) |
| 24 | Valine, leucine and isoleucine biosynthesis | 3 | 21 | 24 (0.27%) | 76 (0.23%) |
| 25 | Lysine biosynthesis | 4 | 7 | 11 (0.12%) | 40 (0.12%) |
| 26 | Lysine degradation | 4 | 22 | 26 (0.29%) | 86 (0.26%) |
| 27 | Arginine and proline metabolism | 9 | 51 | 60 (0.67%) | 268 (0.82%) |
| 28 | Histidine metabolism | 1 | 21 | 22 (0.25%) | 71 (0.22%) |
| 29 | Tyrosine metabolism | 19 | 26 | 45 (0.5%) | 167 (0.51%) |
| 30 | Phenylalanine metabolism | 27 | 87 | 114 (1.28%) | 251 (0.77%) |
| 31 | Tryptophan metabolism | 17 | 35 | 52 (0.58%) | 117 (0.36%) |
| 32 | Phenylalanine, tyrosine and tryptophan biosynthesis | 6 | 21 | 27 (0.3%) | 134 (0.41%) |
| 33 | Benzoxazinoid biosynthesis | 4 | 10 | 14 (0.16%) | 46 (0.14%) |
| 34 | β-alanine metabolism | 7 | 30 | 37 (0.41%) | 205 (0.63%) |
| 35 | Taurine and hypotaurine metabolism | 2 | 8 | 10 (0.11%) | 29 (0.09%) |
| 36 | Selenocompound metabolism | 6 | 19 | 25 (0.28%) | 72 (0.22%) |
| 37 | Cyanoamino acid metabolism | 12 | 35 | 47 (0.53%) | 137 (0.42%) |
| 38 | Glutathione metabolism | 34 | 33 | 65 (0.73%) | 268 (0.82%) |
| 39 | Starch and sucrose metabolism | 75 | 243 | 318 (3.56%) | 897 (2.74%) |
| 40 | N-glycan biosynthesis | 3 | 19 | 22 (0.25%) | 137 (0.42%) |
| 41 | Other glycan degradation | / | 67 | 67 (0.75%) | 214 (0.65%) |
| 42 | Other types of o-glycan biosynthesis | 3 | 3 | 6 (0.07%) | 37 (0.11%) |
| 43 | Amino sugar and nucleotide sugar metabolism | 31 | 89 | 120 (1.34%) | 450 (1.37%) |
| 44 | Glycosaminoglycan degradation | / | 28 | 28 (0.31%) | 132 (0.4%) |
| 45 | Glycerolipid metabolism | 21 | 24 | 45 (0.5%) | 146 (0.45%) |
| 46 | Inositol phosphate metabolism | 20 | 80 | 92 (1.03%) | 299 (0.91%) |
| 47 | Glycosylphosphatidylinositol-anchor biosynthesis | 10 | 45 | 55 (0.62%) | 141 (0.43%) |
| 48 | Glycerophospholipid metabolism | 169 | 334 | 503 (5.63%) | 1345 (4.1%) |
| 49 | Ether lipid metabolism | 138 | 311 | 449 (5.02%) | 1165 (3.55%) |
| 50 | Arachidonic acid metabolism | 2 | 3 | 5 (0.06%) | 30 (0.09%) |
| 51 | Linoleic acid metabolism | 12 | 11 | 23 (0.26%) | 55 (0.17%) |
| 52 | α-Linolenic acid metabolism | 36 | 36 | 72 (0.81%) | 168 (0.51%) |
| 53 | Sphingolipid metabolism | 1 | 47 | 48 (0.54%) | 141 (0.43%) |
| 54 | Glycosphingolipid biosynthesis-globoseries | / | 4 | 4 (0.04%) | 24 (0.07%) |
| 55 | Glycosphingolipid biosynthesis-ganglio series | / | 26 | 26 (0.29%) | 79 (0.24%) |
| 56 | Pyruvate metabolism | 16 | 59 | 75 (0.84%) | 321 (0.98%) |
| 57 | Glyoxylate and dicarboxylate metabolism | 4 | 46 | 50 (0.56%) | 206 (0.63%) |
| 58 | Propanoate metabolism | 11 | 24 | 35 (0.39%) | 146 (0.45%) |
| 59 | Butanoate metabolism | 2 | 18 | 20 (0.22%) | 92 (0.28%) |
| 60 | C_5_-branched dibasic acid metabolism | 3 | 4 | 7 (0.08%) | 14 (0.04%) |
| 61 | One carbon pool by folate | 2 | 10 | 12 (0.13%) | 53 (0.16%) |
| 62 | Carbon fixation in photosynthetic organisms | 13 | 38 | 51 (0.57%) | 284 (0.87%) |
| 63 | Thiamine metabolism | 7 | / | 7 (0.08%) | 27 (0.08%) |
| 64 | Riboflavin metabolism | 3 | 13 | 16 (0.18%) | 55 (0.17%) |
| 65 | Vitamine B_6_ metabolism | 11 | 4 | 15 (0.17%) | 41 (0.13%) |
| 66 | Nicotinate and nicotinamide metabolism | 3 | 3 | 6 (0.07%) | 63 (0.19%) |
| 67 | Pantothenate and CoA biosynthesis | 2 | 23 | 25 (0.28%) | 99 (0.3%) |
| 68 | Lipoic acid metabolism | / | 2 | 2 (0.02%) | 5 (0.02%) |
| 69 | Folate biosynthesis | / | 7 | 7 (0.08%) | 26 (0.08%) |
| 70 | Porphyrin and chlorophyll metabolism | 15 | 60 | 75 (0.84%) | 179 (0.55%) |
| 71 | Terpenoid backbone biosynthesis | 16 | 57 | 73 (0.82%) | 245 (0.75%) |
| 72 | Indole alkaloid biosynthesis | 4 | 4 | 8 (0.09%) | 24 (0.07%) |
| 73 | Monoterpenoid biosynthesis | 4 | 1 | 5 (0.06%) | 25 (0.08%) |
| 74 | Limonene and pinene degradation | 35 | 54 | 89 (1%) | 215 (0.66%) |
| 75 | Diterpenoid biosynthesis | 38 | 22 | 60 (0.67%) | 146 (0.45%) |
| 76 | Brassinosteroid biosynthesis | 4 | 19 | 23 (0.26%) | 65 (0.2%) |
| 77 | Carotenoid biosynthesis | 14 | 55 | 69 (0.77%) | 183 (0.56%) |
| 78 | Zeatin biosynthesis | 13 | 63 | 76 (0.85%) | 215 (0.66%) |
| 79 | Sesquiterpenoid and triterpenoid biosynthesis | 38 | 4 | 42 (0.47%) | 78 (0.24%) |
| 80 | Nitrogen metabolism | 6 | 31 | 37 (0.41%) | 136 (0.41%) |
| 81 | Sulfur metabolism: reduction and fixation | 13 | 21 | 34 (0.38%) | 79 (0.24%) |
| 82 | Phenylpropanoid biosynthesis | 68 | 148 | 216 (2.42%) | 622 (1.9%) |
| 83 | Flavonoid biosynthesis | 75 | 83 | 158 (1.77%) | 340 (1.04%) |
| 84 | Anthocyanin biosynthesis | 6 | 1 | 7 (0.08%) | 17 (0.05%) |
| 85 | Isoflavonoid biosynthesis | 4 | 11 | 13 (0.15%) | 29 (0.09%) |
| 86 | Flavone and flavonol biosynthesis | 20 | 41 | 61 (0.68%) | 144 (0.44%) |
| 87 | Stilbenoid, diarylheptanoid and gingerol biosynthesis | 44 | 72 | 116 (1.3%) | 282 (0.86%) |
| 88 | Isoquinoline alkaloid biosynthesis | 2 | 8 | 10 (0.11%) | 42 (0.13%) |
| 89 | Tropane, piperidine and pyridine alkaloid biosynthesis | 2 | 9 | 11 (0.12%) | 45 (0.14%) |
| 90 | Glucosinolate biosynthesis | 3 | 23 | 26 (0.29%) | 43 (0.13%) |
| 91 | Aminoacyl-tRAN biosynthesis | 9 | 17 | 26 (0.29%) | 243 (0.74%) |
| 92 | Biosynthesis of unsaturated fatty acids | 5 | 12 | 17 (0.19%) | 85 (0.26%) |
| 93 | ABC transporters | 52 | 45 | 97 (1.09%) | 387 (1.18%) |
| 94 | Ribosome biogenesis in eukaryotes | 24 | 152 | 176 (1.97%) | 750 (2.29%) |
| 95 | Ribosome | 74 | 570 | 644 (7.21%) | 1402 (4.28%) |
| 96 | RNA transport | 87 | 257 | 354 (3.96%) | 1240 (3.78%) |
| 97 | mRNA surveillance pathway | 66 | 136 | 202 (2.26%) | 800 (2.44%) |
| 98 | RNA degradation | 24 | 133 | 157 (1.76%) | 744 (2.27%) |
| 99 | RNA polymerase | 51 | 150 | 201 (2.25%) | 572 (1.74%) |
| 100 | Basal transcription factors (eukaryotes) | 3 | 21 | 24 (0.27%) | 186 (0.57%) |
| 101 | DNA replication | 1 | 48 | 49 (0.55%) | 191 (0.58%) |
| 102 | Spliceosome | 57 | 156 | 213 (2.38%) | 1085 (3.31%) |
| 103 | Proteasome | 3 | 15 | 18 (0.2%) | 143 (0.44%) |
| 104 | Protein export | / | 19 | 19 (0.21%) | 179 (0.55%) |
| 105 | Base excision repair | 1 | 27 | 28 (0.31%) | 127 (0.39%) |
| 106 | Nucleotide excision repair | 2 | 59 | 61 (0.68%) | 261 (0.8%) |
| 107 | Mismatch repair | / | 29 | 29 (0.32%) | 122 (0.37%) |
| 108 | Homologous recombination | 4 | 45 | 49 (0.55%) | 265 (0.81%) |
| 109 | Non-homologous end-joining | / | 6 | 6 (0.07%) | 45 (0.14%) |
| 110 | Phosphatidylinositol signaling system | 35 | 55 | 90 (1.01%) | 303 (0.92%) |
| 111 | Plant hormone signal transduction | 156 | 465 | 621 (6.95%) | 1746 (5.32%) |
| 112 | Ubiquitin mediated proteolysis | 18 | 97 | 115 (1.29%) | 589 (1.8%) |
| 113 | Sulfur relay system | / | 4 | 4 (0.04%) | 19 (0.06%) |
| 114 | Snare interactions in vesicular transport | 11 | 21 | 32 (0.36%) | 131 (0.4%) |
| 115 | Regulation of autophagy | 11 | 21 | 32 (0.36%) | 198 (0.6%) |
| 116 | Protein processing in endoplasmic reticulum | 30 | 168 | 198 (2.22%) | 895 (2.73%) |
| 117 | Endocytosis | 154 | 378 | 532 (5.95%) | 1517 (4.63%) |
| 118 | Phagosome | 18 | 92 | 110 (1.23%) | 438 (1.34%) |
| 119 | Peroxisome | 14 | 27 | 41 (0.46%) | 289 (0.88%) |
| 120 | Plant-pathogen interaction | 232 | 395 | 627 (7.02%) | 1990 (6.07%) |
| 121 | Natural killer cell mediated cytotoxicity | 14 | 24 | 38 (0.43%) | 121 (0.37%) |
| 122 | Circadian rhythm | / | 10 | 10 (0.11%) | 105 (0.32%) |
| 123 | Circadian rhythm-plant | 28 | 80 | 108 (1.21%) | 293 (0.89%) |
| 124 | Metabolic pathways | / | / | 2441(27.32%) | 8071(24.61%) |
| 125 | Biosynthesis of secondary metabolites | / | / | 1079(12.07%) | 3557(10.85%) |
