# Supplementary material for: Deep sequencing reveals transcriptome re-programming of Polygonum multiflorum thunb. roots to the elicitation with methyl jasmonate
Source: Mol Genet Genomics. 2015 Sep 5;291:337–48. doi: 10.1007/s00438-015-1112-9 (PMC4729805; doi:10.1007/s00438-015-1112-9)
Supplement: Supplementary file 13 — Supplementary material 13 (DOC 411 kb) [file 438_2015_1112_MOESM13_ESM.doc]

**Table S6** Validation of up- or down-regulation of 70 genes of interest (GOIs) obtained from transcriptome sequencing of MeJA root-irrigation-treated samples of *Polygonum* *multiflorum*. A, B and C correspond to reference genes (RGs) *UBQ14*, *UBQ4-1*, and *SAMS*, respectively. GOI serial numbers are the same as in Table S3. Three RGs were selected for validation of the up- or down-regulation of each GOI obtained from transcriptome sequencing. If the normalized expression value of a GOI was greater than 1, the GOI was considered to be up- regulated. Down-regulated genes were those with normalized expression values less than 1.

| GOI | Expression profile in RNA-seq data | RG | Norm. Expression | Remarks | GOI | Expression profile in RNA-seq data | RG | Norm. Expression | Remarks | GOI | Expression profile in RNA-seq data | RG | Norm. Expression | Remarks |
| --- | --- | --- | --- | --- | --- | --- | --- | --- | --- | --- | --- | --- | --- | --- |
| 1 | 1.0200/Up | A | 1.6593 | Up | 25 | 1.2038/Up | A | 13.4100 | Up | 48 | 1.8590/Up | A | 0.7917 | Down |
|  |  | B | 3.7599 | Up |  |  | B | 14.5043 | Up |  |  | B | 0.6090 | Down |
|  |  | C | 6.0906 | Up |  |  | C | 28.8255 | Up |  |  | C | 2.3327 | Up |
| 2 | 2.6674/Up | A | 1.9789 | Up | 26 | 3.3290/Up | A | 18.7135 | Up | 49 | 3.1671/Up | A | 1.8411 | Up |
|  |  | B | 1.9705 | Up |  |  | B | 20.2406 | Up |  |  | B | 1.8332 | Up |
|  |  | C | 1.2490 | Up |  |  | C | 40.2257 | Up |  |  | C | 1.1619 | Up |
| 3 | 2.3859/Up | A | 0.8731 | Down | 27 | 1.1852/Up | A | 2.1345 | Up | 50 | 3.8210/Up | A | 430.7607 | Up |
|  |  | B | 0.8307 | Down |  |  | B | 1.0367 | Up |  |  | B | 432.2252 | Up |
|  |  | C | 1.0088 | Up |  |  | C | 0.5833 | Down |  |  | C | 977.8709 | Up |
| 4 | 2.1982/Up | A | 1.0386 | Up | 28 | 2.0610/Up | A | 0.7169 | Down | 51 | -1.9211/Down | A | 0.26250 | Down |
|  |  | B | 0.9882 | Down |  |  | B | 0.7193 | Down |  |  | B | 0.3468 | Down |
|  |  | C | 1.2001 | Up |  |  | C | 1.6274 | Up |  |  | C | 0.0513 | Down |
| 5 | 1.2242/Up | A | 0.9028 | Down | 29 | 1.6990/Up | A | 1.3071 | Up | 52 | -2.0226/Down | A | 0.0513 | Down |
|  |  | B | 1.4383 | Up |  |  | B | 1.0055 | Up |  |  | B | 0.0291 | Down |
|  |  | C | 2.6741 | Up |  |  | C | 3.8514 | Up |  |  | C | 0.0678 | Down |
| 6 | 4.9758/Up | A | 0.9428 | Down | 30 | 3.6982/Up | A | 3.1225 | Up | 53 | -1.1219/Down | A | 0.3165 | Down |
|  |  | B | 0.8970 | Down |  |  | B | 14.7466 | Up |  |  | B | 0.6924 | Down |
|  |  | C | 1.0894 | Up |  |  | C | 3.3139 | Up |  |  | C | 1.1216 | Up |
| 7 | 2.009/Up | A | 5.5281 | Up | 31 | 4.8210/Up | A | 0.9823 | Down | 54 | -1.5971/Down | A | 0.1291 | Down |
|  |  | B | 5.2596 | Up |  |  | B | 4.6390 | Up |  |  | B | 0.2824 | Down |
|  |  | C | 6.3877 | Up |  |  | C | 1.0425 | Up |  |  | C | 0.4575 | Down |
| 8 | 2.3654/Up | A | 3.3854 | Up | 32 | 1.0276/Up | A | 1.2525 | Up | 55 | -2.4382/Down | A | 0.1400 | Down |
|  |  | B | 4.4728 | Up |  |  | B | 2.9467 | Up |  |  | B | 0.0794 | Down |
|  |  | C | 0.2044 | Down |  |  | C | 2.5470 | Up |  |  | C | 0.1851 | Down |
| 9 | 2.2361/Up | A | 1.9406 | Up | 33 | 1.2200/Up | A | 0.5454 | Down | 56 | -1.3878/Down | A | 0.4182 | Down |
|  |  | B | 3.9834 | Up |  |  | B | 1.2830 | Up |  |  | B | 0.2372 | Down |
|  |  | C | 2.2424 | Up |  |  | C | 1.1090 | Up |  |  | C | 0.5531 | Down |
| 10 | 1.1145/Up | A | 1.0216 | Up | 34 | 1.2988/Up | A | 0.5882 | Down | 57 | -2.6004,Down | A | 0.5788 | Down |
|  |  | B | 4.7823 | Up |  |  | B | 0.4525 | Down |  |  | B | 2.2073 | Up |
|  |  | C | 1.0747 | Up |  |  | C | 1.7331 | Up |  |  | C | 1.5850 | Up |
| 11 | 1.4991/Up | A | 1.0428 | Up | 35 | 2.0276/Up | A | 1.4319 | Up | 58 | -0.1110/Down | A | 0.0675 | Down |
|  |  | B | 1.0383 | Up |  |  | B | 1.1015 | Up |  |  | B | 0.0058 | Down |
|  |  | C | 0.8468 | Down |  |  | C | 4.2192 | Up |  |  | C | 0.0342 | Down |
| 12 | 2.6813/Up | A | 3.0219 | Up | 36 | 1.5844/Up | A | 1.3831 | Up | 59 | -3.8691/Down | A | 0.0920 | Down |
|  |  | B | 4.8143 | Up |  |  | B | 1.8311 | Up |  |  | B | 0.0232 | Down |
|  |  | C | 8.9506 | Up |  |  | C | 2.0702 | Up |  |  | C | 0.2252 | Down |
| 13 | 1.5223/Up | A | 1.5265 | Up | 37 | 3.4324/Up | A | 1.1087 | Up | 60 | -1.4818/Down | A | 0.0401 | Down |
|  |  | B | 2.0168 | Up |  |  | B | 1.0549 | Up |  |  | B | 0.0878 | Down |
|  |  | C | 0.3707 | Down |  |  | C | 1.2811 | Up |  |  | C | 0.1422 | Down |
| 14 | 2.2486/Up | A | 1.6648 | Up | 38 | 1.0711/Up | A | 0.4384 | Down | 61 | -1.3605/Down | A | 0.0469 | Down |
|  |  | B | 2.1996 | Up |  |  | B | 1.0314 | Up |  |  | B | 0.1026 | Down |
|  |  | C | 0.0746 | Down |  |  | C | 0.8914 | Down |  |  | C | 0.1662 | Down |
| 15 | 1.3550/Up | A | 11.9031 | Up | 39 | 1.7215/Up | A | 1.4056 | Up | 62 | -1.1463/Down | A | 0.0887 | Down |
|  |  | B | 15.7265 | Up |  |  | B | 1.3996 | Up |  |  | B | 0.0513 | Down |
|  |  | C | 1.3918 | Up |  |  | C | 0.8871 | Down |  |  | C | 0.1196 | Down |
| 16 | 1.7506/Up | A | 3.0541 | Up | 40 | 2.5695/Up | A | 43.1840 | Up | 63 | -1.4925/Down | A | 0.0708 | Down |
|  |  | B | 6.6822 | Up |  |  | B | 47.8814 | Up |  |  | B | 0.1549 | Down |
|  |  | C | 10.8243 | Up |  |  | C | 52.1335 | Up |  |  | C | 0.2509 | Down |
| 17 | 1.0197/Up | A | 0.4171 | Down | 41 | 2.6666/Up | A | 1.3231 | Up | 64 | -0.2074/Down | A | 0.7239 | Down |
|  |  | B | 0.6646 | Down |  |  | B | 1.3174 | Up |  |  | B | 0.9565 | Down |
|  |  | C | 1.2355 | Up |  |  | C | 0.8350 | Down |  |  | C | 2.9069 | Up |
| 18 | 1.7704/Up | A | 0.4878 | Down | 42 | 3.7158/Up | A | 1.0530 | Up | 65 | -1.0285/Down | A | 0.7579 | Down |
|  |  | B | 0.7771 | Down |  |  | B | 1.0018 | Up |  |  | B | 1.0014 | Up |
|  |  | C | 1.4448 | Up |  |  | C | 1.2167 | Up |  |  | C | 1.7350 | Up |
| 19 | 2.0177/Up | A | 1.3710 | Up | 43 | 3.7129/Up | A | 0.8989 | Down | 66 | -1.1136/Down | A | 0.2313 | Down |
|  |  | B | 2.1843 | Up |  |  | B | 0.6915 | Down |  |  | B | 0.5060 | Down |
|  |  | C | 4.0609 | Up |  |  | C | 2.6486 | Up |  |  | C | 0.8197 | Down |
| 20 | 2.3626/Up | A | 0.3645 | Down | 44 | 2.3679/Up | A | 0.6940 | Down | 67 | -1.1661/Down | A | 2.3093 | Up |
|  |  | B | 0.7976 | Down |  |  | B | 3.2774 | Up |  |  | B | 0.1973 | Down |
|  |  | C | 1.2920 | Up |  |  | C | 0.7365 | Down |  |  | C | 1.1708 | Up |
| 21 | 1.6801/Up | A | 2.8722 | Up | 45 | 1.5964/Up | A | 7.4977 | Up | 68 | -2.4393/Down | A | 0.9220 | Down |
|  |  | B | 6.5677 | Up |  |  | B | 5.7676 | Up |  |  | B | 0.5229 | Down |
|  |  | C | 10.6390 | Up |  |  | C | 22.0915 | Up |  |  | C | 1.2194 | Up |
| 22 | 2.2485/Up | A | 1.0201 | Up | 46 | 2.7215/Up | A | 0.3452 | Down | 69 | -1.1914/Down | A | 1.1705 | Up |
|  |  | B | 1.0157 | Up |  |  | B | 0.7552 | Down |  |  | B | 1.1655 | Up |
|  |  | C | 1.2533 | Up |  |  | C | 1.2234 | Up |  |  | C | 0.7388 | Down |
| 23 | 1.6285/Up | A | 782.4474 | Up | 47 | 1.4991/Up | A | 1.4990 | Up | 70 | -1.0103/Down | A | 0.8349 | Down |
|  |  | B | 846.3021 | Up |  |  | B | 1.4925 | Up |  |  | B | 1.1030 | Up |
|  |  | C | 1681.9184 | Up |  |  | C | 0.9460 | Down |  |  | C | 3.3007 |  |
| 24 | 1.0059/Up | A | 0.4132 | Down |  |  |  |  |  |  |  |  |  |  |
|  |  | B | 1.9516 | Up |  |  |  |  |  |  |  |  |  |  |
|  |  | C | 0.4386 | Down |  |  |  |  |  |  |  |  |  |  |
