# Supplementary material for: Deep sequencing reveals transcriptome re-programming of Polygonum multiflorum thunb. roots to the elicitation with methyl jasmonate
Source: Mol Genet Genomics. 2015 Sep 5;291:337–48. doi: 10.1007/s00438-015-1112-9 (PMC4729805; doi:10.1007/s00438-015-1112-9)
Supplement: Supplementary file 14 — Supplementary material 14 (DOC 109 kb) [file 438_2015_1112_MOESM14_ESM.doc]

**Table S7** Validation of raw sequences of 13 genes of interest (GOI) by sequencing of their PCR products. Shared identities between raw sequences and sequences of their corresponding PCR amplicons varied from 91.05 to 100%.

| **Number** | **Gene ID** | **Gene description** | **Homological species** | **Nr-ID** | **Primer sequences (forward/reverse)** | **Blast cds of raw sequences** | **Amplicon sequences (bp)** | **Amplicon length (bp)** | **Identity(%)** |
| --- | --- | --- | --- | --- | --- | --- | --- | --- | --- |
| **1** | Unigene12471_All | Coumaroyl-CoA ligase | Vitis vinifera | gi|371534669|gb|AEX32786.1| | TCCACATCTACTCCCTCAACTCC/  CAGGAACCTCACCAGCAACT | ACGGAAACGGTAGAAGAAGGATTCGACTCATCGGAGGCGGTGGCGCTGCCGTTCTCCTCGGGAACAACAGGGCTACCAAAGGGGGTGGTGCTGACGCACAAGAGTCTGATAACGAGCGTCGCACAACAAGTGGACGGGGAAAACCCGAATCTCTACCTGACCCCGGACGATGTCGTTTTGTGCTTGCTTCCCCTATTCCACATCTACTCCCTCAACTCCGTCCTCCTCTGCGCCATGAGGGCCGGCAGCGCCGTCCTCCTCGTCCACAAGTTCGAGATCGCCGCCCTGCTCGAGCAGCTCGAGCGCCACCGCGTCACGGTGGCCGCCGTTGTCCCGCCTCTCGTCCTGGCCCTCGCCAAGAATCCGATGGTGGAGAAGTTCGACCTCGGCGCCATCAGGGCGGTGCTCTCCGGGGCGGCGCCGCTTGGAAAGGAGCTCGAGGACGCCCTCCGGAGTCGGCTCCCGCAGGCCGTTCTCGGTCAGGGTTATGGAATGACGGAGGCAGGGCCAGTGTTAGCAATGTGCCTGGGATTTGCAAAGGAGCCCTTCCCCACCAAGCCCGGCTCTTGTGGATCCGTCGTCCGCAACGCTGAGATGAAGGTTGTCGATCCCGAAACCGGATCCTCTCTCGGCTTCAATCAACCCGGTGAGATCTGCATCCGTGGAGATCAGATCATGAAAGGGTATCTAAACGATGACGCGGCCACAGCTGCAACCATAGACGCCCAAGGCTGGCTTCATACTGGGGACATTGGCTACGTCGATGAAGACATCGAGGTTTTCATTGTCGACCGAGTAAAGGAGATCATTAAATTCAAAGGATTCCAGGTAGCGCCAGCAGAGCTAGAGGCGCTTCTAGTGAGCCATCCCTCCATAGCCGATGCAGCCGTTGTCCCGCAAAAGGATGAAGTTGCTGGTGAGGTTCCTGTGGCGTTTGTGGTTCGGTCTGAAGGGTTTGAGCTTACAGAAGATGCAGTCAAAGAATTCGTAGCAAAGCAGGTGGTTTTCTACAAGAAGCTGCACAAGGTGTACTTTGTTCACGCGATCCCGAAGTCTCCGTCTGGTAAAATCCTGAGGAAAGAGCTCCGAGCTAAGTTGGCT | TTCCACATTCTTACCCTCAACTCCGTCCTCCTCTGCGCCATGAGGGCCGGCAGCGCCGTCCTCCTCGTCCACAAGTTCGAGATCGCCGCCCTGCTCGAGCAGCTCGAGCGCCACCGCGTCACGGTGGCCGCCGTTGTCCCGCCTCTCGTCCTGGCCCTCGCCAAGAATCCGATGGTGGAGAAGTTCGACCTCGGCTCCATCAGGGCGGTGCTCTCCGGGGCGGCGCCGCTTGGAAAGGAGCTCGAGGACGCCCTCCGGAGTCGGCTCCCGCAGGCCGTTCTCGGTCAGGGTTATGGAATGACGGAGGCAGGGCCAGTGTTAGCAATGTGCCTGGGATTTGCAAAGGAGCCCTTCCCCACCAAGCCCGGCTCTTGTGGATCCGTCGTCCGCAACGCTGAGATGAAGGTTGTCGATCCCGAAACCGGATCCTCTCTCGGCTTCAATCAACCCGGTGAGATCTGCATCCGTGGAGATCAGATCATGAAAGGGTATCTAAACGATGACGCGGCCACAGCTGCAACCATAGACGCCCAAGGCTGGCTTCATACTGGGGACATTGGCTACGTCGATGAAGACATCGAGGTTTTCATTGTCGACCGAGTAAAGGAGATCATTAAATTCAAAGGATTCCAGGTAGCGCCAGCAGAGCTAGAGGCGCTTCTAGTGAGCCATCCCTCCATAGCCGATGCAGCCGTTGTCCCGCAAAAGGATGAAGTTGCTGGGAAAGGTTCCTGAA | 736 | 98.64 |
| **2** | Unigene43841_All | Cinnamate 4-hydroxylase | Fagopyrum esculentum | gi|314910736|gb|ADT63059.1| | GAGCTAGTAAACCACCCAGACA/  GGTAAGGGAGGTTGGTGAGAT | ATAGCAGAGCTAGTAAACCACCCAGACATCCAAGCCAAGCTTCGAGCCGAGCTCGATACCGTCCTGGGCAAAGGAGCCCAGATAACCGAGCCCGATCTCACCAACCTCCCTTACCTCCAA | GAGCTAGTAAACCACCCAGACATCCAAGCCAAGCTTCGAGCCGAGCTCGATACCGTCCTGGGCAAAGGAGCCCAGATAACCGAGCCCGATCTCACCAACCTCCCTTACCT | 110 | 100.00 |
| **3** | Unigene19556_All | cinnamate 4-hydroxylase | Salvia miltiorrhiza | gi|85720056|gb|ABC75596.1| | CCGCAGTTGGAACCTGAGAAG/  AGGCAGAGGCTTACCGTTAGG | GAACTCGACACCGTCCTCGGAAAGGGCGTCCAGATAACGGAGCCCGACATCCAAAGACTCCCTTACCTTTACGCCGTCATCAAGGAAGCGCTCCGTCTCCGAATGGCAATCCCTCTGCTGGTCCCACACATGAACCTCGACGACGCCAAGCTGGCGGGCTACGACATCCCGGCGGAGAGCAAGATCCTGGTCAACGCCTGGTGGCTGGCCAACAACCCCGACACCTGGAAGAATCCCGAGGAGTTCCGCCCCGAACGCTTCTTGGAAGAAGAGTCCAAGGTTGACGCCAACGGTAACGATTTCCGTTACTTGCCGTTTGGCGTCGGACGGAGGAGTTGCCCCGGTATTATTCTGGCCTTGCCTATTGCGGGAATTACCGTTGGGAGGTTGGTTCAGAATTTCGAGCTTCTTCCGCCGCCAGGGAAGGACAAGGTTGATACTTCTGAGAAAGCTGGGCAATTTAGCTTGCAGATTCTTAACCACTCCACCATTGTTCTTAAGCCAAGGAGC | TGAGCCCGAACACCAAAGACTCCCTTACCTTTACGCCGTCATCAAGGAAGCGCTCCGTCTCCGAATGGCAATCCCTCTGCTGGTCCCACACATGAACCTCGACGACGCCAAGCTGGCGGGCTACGACATCCCGGCGGAGAGCAAGATCCTGGTCAACGCCTGGTGGCTGGCCAACAACCCCGACACCTGGAAGAATCCCGAGGAGTTCCGCCCCGAACGCTTCTTGGAAGAAGAGTCCAAGGTTGACGCCAACGGTAACGATTTCCGTTACTTGCCGTTTGGCGTCGGMCGGAGGAGTTGCCCCGGTATTATTCTGGCCTTGCCTATTGCGGGAATTACCGTTGGGAGGTTGGTTCAGAATTTCGAGCTTCTTCCGCCGCCAGGGAAGGACAAGGTTGATACTTCTGAGAAAGCTGGGCAATTTAGCTTGCAGATTCTTAACCACTCCACCA | 452 | 98.89 |
| **4** | CL2476.Contig2_All | UDP-glucosyltransferase | Ricinus communis | gi|223546179|gb|EEF47681.1| | CCCAGGATCGCCTTCTACACCT/  TCGTCGAGCCAGCTTAACACCT | ATCCCCAGGATCGCCTTCTACACCTCCGGATCCTTCGTTTCCTCCGTCAGCGCCACTCTGTTTTCCGACGTTGCGAGATTCAAGGCGCTCGATTCCGTCCAATTTACCGATCTGCCGCGCTCCCCTGTTTTCAAGGAGGAGCATCTGCCAACCGTCTTCAGACTCTACAACGAATCCGATCGCAGTTCTCATGCTATCAAGGACGGATTGATGGCGAACTTGTCCAGCTGGGGCTGCGTTTACAATTCGTTTCGCGCCCTCGAAGGCGACTACTTGGAGCATATCACGAAGGTGACCGTCAACGGGAGAGTCTACGGCGTCGGTCCGGTTAGTTTGTTCAAGGAATCCGCGACTCTAAGTCGGGTCAACCCGGATCAGGATGTTAACTGCGAGGTGTTAAGCTGGCTCGACGATTGCCCG | TCCCAGGATTCGCTTCTACACCTCCGGATCCTTCGTTTCCTCCGTCAGCGCCACTCTGTTTTCCGACGTTGCGAGATTCAAGGCGCTCGATTCCGTCCAATTTACCGATCTGCCGCGCTCCCCTGTTTTCAAGGAGGAGCATCTGCCAACCGTCTTCAGACTCTACAACGAATCCGATCGCAGTTCTCATGCTATCAAGGACGGATTGATGGCGAACTTGTCCAGCTGGGGCTGCGTTTACAATTCGTTTCGCGCCCTCGAAGGCGACTACTTGGAGCATATCASGAAGGTGACCGTCAACGGGAGAGTCTACGGCGTCGGTCCGGTTAGTTTGTTCAAGGAATCCGCGACTCTAAGTCGGGTCAACCCGGATCAGGATGTTAACTGCGAGGTGTTAAGCTGGCTCGACGA | 411 | 98.78 |
| **5** | CL8199.Contig1_All | UDP-glucose glucosyltransferase | Fragaria x ananassa | gi|51705413|gb|AAU09444.1| | CTCTCCATCACCGTCCTCCT/  CGTCGAGTTCCATCCACAGT | ATTTCAAAGACGGCAAATCTAGTACTGATCCCAGCTCCGGGAATGGGCCACCTTGCCCCCGCCATAGAGCTGGCCAAGCACCTGGTGGCCCGGGATCCGCGCCTCTCCATCACCGTCCTCCTCATCAAGGGTTTCGTCCCCGAGGAAACGCTCGAATCTTATATCCTCTCTCTGAAATCCAACTCCTCTCTTCTCAACCAAGGCATCGGATTCATTGATATTCCCCAAATCGACCTCAACTCCGCCAGACCGAGCTTCTTGACTCACATGAACGCCTACCAACCCAAAGTCAAGGAAGCCATCGAGGGTCTTCGATCCGCGGGTCCCCTGCCCGTTGCGGGGCTCCTCGTGGACATGTTCTGCACTTCCACGATCGATGTGGCAACAGAGCTTGGGCTACCGTCTTACGTCTTCTTCACCTCCGGCGCCGGCCTTCTCCGCATGTTTTTCCACCTCCATGGATTAGGGGTCGATGTGGCGCAGGAATACGATGTCGTTCGGAGCCCGGATACGTTGTTGGAGATTCCAGGGTTTCGAAATCCGGTGCCGGTGAAAGTTTTGCCGGGGAGATTCCTGTCTAAAGACGGTCAGTCATCCACGTTCTTGCGCCTCGCTGACAAGTTCCGCCAGGCCAAGGGTATTTTGGTAAATACATGCATGGAGATTGATCGGGACTTAATCCAGTCCATGTCGCAGCAAGACATCGAAATCCCACCGATCTACCCGGTGGGGCCCATCCTCAACCTGCCGACTGAAGATGATCATGGTCATGATGATGACGAGTCCTCCGGGAAAGATCCAATCACGAGGTGGCTGGACGACCAGCCGCCTCGATCCGTGGTGTTCCTCTGCTTCGGGAGCAGGGGAACCTTCAACGACACGCAGATCAAAGAGATCGCCGTCGGGCTCGAGCGAAGTGGCCAACGCTTTCTGTGGTCCCTCCGGCAACGACCCGGTGAGACCGGGGTTCCATTGGAATTAGATGATCCTAGTAAGGTGTTGCCGGAGGGGTTTTTTGAGCGGACAGCCGAGAAAGGGAGGGTCATAGGGTGGGCCCCGCAGGCGAGGGTGTTGGCCCACAAGGCGGTTGGGGCATTCGTGTCGCACTGTGGATGGAACTCGACG | TCTCTTCTCAACCAATGGCATCRGAYWCATYGATATTCYCCAAATCGACCTCAAMTYCGCCAGAMCGAGCTTCTTGACTCACATGAACGCYTACCAACCCAAAGTCAAGGAAGMCATCGA GGRTSTYMGATCCGCKGGTCCCYTSSCCGTTGCGGGGCTCCTCGTGGACATGTTCTGCACTTCCACGATCGATGTGGCAACAGAGCTTGGGMYASMGTCTKACGTCTTSTTCACCTCCGGCGCCGGCYTTSTCCGCKTGTTTTTCCACCTCCATGGATTAGGGGTCGATGTGGCGCAGGAATASGATGTCGTTCGGAGCCCGGATACGTTGYTGGAGATTCCAGGGTTTCGAAATCCGGTGCCGGTGAAAGTTTTGCCGGGGARATTCCTGTCTAAAGACGGTCAGTCATCCACGYTCTTGCGCCTCGYTGACAAGTTCCGCCAGGCCAAGGGTATTTTSGTAAATACATGCATGGAGATTGATCGGGACYTAATCCAGTCCATGTYGCAGCAAGACATCGAAMTCCCACCGATCTACCCGGTGGGGCCCATCCTCAACCTGCCGACYGAAGATGATCATGGTCATGATGATGACGAGTCCTCCRGGAAAGATCCAATCACGAGGTGGCTGGACGACCAGCCGCCTCGATCCGTGGTGTTCCTCTGCTTCRGGAGCAGGGGAACCTTCAACGACACGCASATCAAAGAGATCGCCGTCGGGCTCGAGCGAAGTGGCCAACGCTTTYTGTGGTCCCTCCGGCAACGACCCGGTGAGACCGGGGTTCCATTGSAWTTAGATGAKCCTAGTAAGGTGTTGCCKGAGGGGTTTTTTGAGCGGACAGCCGAGAAAGGGAGGGTCATA | 862 | 94.77 |
| **6** | Unigene6757_All | glucosyltransferase | Nicotiana tabacum | gi|14349253|dbj|BAB60721.1| | CCATCTCCTCGCCGAAGTTG/  CCGTTACTCCGTCGTTAGCCT | GCCGAACTCATCCTCTTCGCCGGCCCGGAGGTGGGCCATCTCCTCGCCGAAGTTGAGGTTAGCCGGCGAGTCACATCCTTAGACGCCCAAATCTCCATAGCCATCCTCATCCCCAAGCTCCCCGTCCCCAACGAAAACCTCGACGTCTTCATCAAATCCCTCGACGCCGACCTCCGCCGCGACCCCGACCCGCGAATCTCCGTCGTCGAGCTTCCGCCGCTGGACGACGTCCCGCCGGAGCTGACCCAACCCGGGAACCACGTCCTCCTAGTCGACAGCCTCGCGGGTCTCTACAAGCCGATCGTGAAGCGCACGGTCGAGGAGCGCCGGCTCCGGCGCAAAACCGCCGGAATCGTCTTCGACATGTTCTGCGCGCCGATGGTCGACGTGGCGGAGGAGCTCGGCGTGCCGTCCTACTTGTTCTACACCTCCGGCGCTAACATGCTCAGCTTAACTCTCAAGCTGGAGTCGCTCGCGGCGGACGACCTGCCGGCGCTCTTCGAAGGAATGTCGCCGGAGTCAACGCCGGTCGAATTCCCGGGATTCAAGAATCCCGTGCCGTTGAAGGTCTGGCCCGAGCTCTATCTAAACAAAGGGGCAGCGCTCTGGGCCCACTTCATGCGGTTCGCTTCTCAGTATCGCAAAACCAAGGGCATTTTGGTAAATACATTCACCGAGTTGGAGTCGGAACTCCTTAAGTCCTTGAACGAAAACGAACGTTTTCCTCCCGTTTATTCCGTGGGCCCCATCCTCAACCTCGGTCGTAAGGCTAACGACGGAGTAACGGAATCGCCTCGGGATCCGACGCTGAGTTGGTTAGACGAGCAACCGGCTGGGTCGGTGGTTTTCTTCTGCTTCGGGAGCAAG | GCGAAGTTGAGGTTAGCCGGCGAGTCACATCCTTAGMYKSMSMMMTYYYCMKAGCCWTCCTCATCCCCAAGCTCCCCGTCCCCAACGAAAACCTCGRCGTCTTCATCAAATCCCTCGACGCCGACCTCCGCCGCGACCCCGACCCGCGAATCTCCGTCGTCGAGCTTCCGCCGCTGGACGACGTCCCGCCGGAGCTGACCCAACCCGGGAACCACGTCCTCCTAGTCGACAGCCTCGCGGGTCTCTACAAGCCGATCGTGAAGCGCACGGTCGAGGAGCGCCGGCTCCGGCGCAAAACCGCCGGAATCGTCTTCGACATGTTCTGCGCGCCGATGGTCGACGTGGCGGAGGAGCTCGGCGTGCCGTCCTACTTGTTCTACACCTCCGGCGCTAACATGCTCAGCTTAACTCTCAAGCTGGAGTCGCTCGCGGCGGACGACCTGCCGGCGCTCTTCGAAGGAATGTCGCCGGAGTCAACGCCGGTCGAATTCCCGGGATTCAAGAATCCCGTGCCGTTGAAGGTCTGGCCCGAGCTCTATCTAAACAAAGGGGCAGCGCTCTGGGCCCACTTCATGCGGTTCGCTTCTCAGTATCGCAAAACCAAGGGCATTTTGGTAAATACATTCACCGAGYWGGAGTCGGAACTCCWTAAGTCCTTGAACGAAAACGAACGTTTTCCYCCCGTKWWTTCCGTGGGCCCCATCCTCAACCTCGGTCGTAAGGCTAACGCG | 731 | 96.44 |
| **7** | Unigene15430_All | UDP-glucosyltransferase | Ricinus communis | gi|223537474|gb|EEF39100.1| | ACCCTTCCCTCATCACTTTCC/  GGCATCCTTCACAGCCTTACT | TCCAAGTTCCACATCCTCATGTTCCCGTGGCTCGCCATCGGCCACATAACTCCCTACCTCCACCTCTCCAACAAACTCGCCGAAAAAGGCCACAAAATCACATTCCTCCTCCCAAACAAGGCTCAAGTCCTCCTCCAACACCTCAACCTCCACCCTTCCCTCATCACTTTCCACCCACTCTCGATCCCCCAAGTGGAAGGCCTTCCTCTTGGCACCGAGACTGCCTCGGACGTCCCCATCTCCCTCACACGCTTTCTCGCCATCGCATTCGACCTGACGCGCCCCCAAGTCTCACAAATCATACTCGACCTCAAACCGGATTTCGTCTTCTACGACTTCGCTCACTGGGTCCCCGAGATCACGTCCAAGCTCGGGATCAAGTCGGTATGTTATAACGTAGTATGCGCCGCCGCCCTGGCCCATAACATGGTCCCCGTGCGTTACACTTGTCACCCTCCACTTACGGCGGAGCAGGTGGAGGAGCTGGTTGCGCCGCCCGCAGGATACCCCATGGCGCTTTTGCACAAGCACGAGGCGCGTAAGCTCGTGTTCATGGCCCAAGAATATGGCAGCGGAATCACCTTCTTCGAGAGGCTCAACTCGGCACTGAAGTTGGCCGACGCCATCGCTATCAGGACTTGCAAGGAGATCGAGGGGAAGTTCTGTGATTACCTCAGCAGCCAGTTTGAGACATCAGTGTTACTCACGGGCCCCGTGTTGCCGGACTTGACCACCACCGAAGGGACGGCGTTGGATGAGAAATGGGCGAGCTGGCTCGGAGCCCACGAACCGGGTTCAGTGATTTACTGCGCGTTCGGGACCCAGTGTTTCCTTGACAAGCACCAGTATCAGGAGATTCTTTTAGGGTTCGAGTTAACTGGTTATCCGTTCTTTGTGGCCCTAAAGCCTCCTACGGGATGCTCGACGATAGAGGAGGCTTTACCAGAGGGGTTTGAAGAGCGGGTCAGGGGGAAGGGGGTGGTTTACGGAGGGTGGGACATCCAGCAACCGCTCATACTGGAGCACCCGTCGGTTGGGTGCTTCGTGAGCCATTGTGGGTTCGGGTCCATGTGGGAGTCGCTTATGGGGAAGCCTCAAATAGTGTTGGTGCCACAGCAGGGTGACCAGGTAATGAATACGAGGATCATGGCGCGAGAGATGAAGGTGGCAGTGGAGGTGGAGAAGGACGAGAGTGGGTGGGTTTCAAGGGAGAGCCTGAGTAAGGCTGTGAAGGATGCCATGGATAAGGAGAGCGTGGTGGGGTGTTTGATTAGAGAGAACCATGCTAAGTGGAGGGAGGTGTTGGGGAGCCCTGGCTTCATGAGTGGTTATGTTGACAGCTTTGTGCATCAATTGGAGAGGCTTTTGGAT | GGAAGGCTTCCTCTTGGCACCGAGACTGCCTCGGACGTCCCCATCTCCCTCACACGCTTTCTCGCCATCGCATTCGACCTGACGCSCCCMMRWSTYYMMMAAATCATAACTCGACCTYMAAACCCGGATTTCGTCTTCTACGACTTCGCTCACTGGGTCCCCGAGGATCACGTCCAAGCTCGGGATCAAGTCGGTATGKTATWACGTAGTAWGCGCCGSCGCCCTGGCCCATAACATGGYCCCCGTGCGTTACACTTGTCACCCTCCACTTACGGCGGCGCAGGTGGAGGAGCTGGTTGCGCCGCCCGCAGRATACCCCATGGSGCTTTTGCACAAGCACGAGGSGCGTAAGCTCGKGTTCATGGCCCAAGAATATGGCAGCGGAATCACCTTCTTCGAGAGGYTCAACTCGGCACTGAAGTTGGCCGACGCCATCGCTATCAGGACTTGCAAGGAGATCGAGGGGAAGTTCTGTGATTACCTCAGCAGCCAGTTTGAGACATCAGKGTTACTCACGGGCCCCGCGTTGCCGGACTTGACCACCACCGAAGGRACGGCGTTGGATGAGAAATGGGCGAGCTGGCTCGRAGCCCACGAACCGGGTTCAGTGATTTACTGCGCGTTCGGRACCCAGTGTTTCCTTGACAAGCACCAGTTTCAGGAGATTCTTTTAGGGTTCGAGTTAACTGGTTATCCGTTCTTTGTGGCCCTAAAGCCTCCTACGGGATGCTCGACGATAGAGGAGGCTTTACCAGAGGGGTTTGARGAGCGGGTCAGGGGRAAGGGGGKKGTTTACGGAGGGTGGGACATCCAGCAACCGCTCATACTGGAGCACCCGTCGKKKKGSTKCKTSRKSAKYSWKKGTKSGKKYSRKKYSRWGTCGCTATGGGAGYYSMWWWWGKGKRWGCCTCAAATAGTGTTGGTGCCACAGCAGGGTGACCAGGTAATGAATACGAGGATCATGGCGCGAGAGATGAAGGTGGCAGTGGAGGTGGAGAAGGACGAGAGTGGGTG | 1014 | 92.13 |
| **8** | CL554.Contig1_All | glucosyltransferase | Nicotiana tabacum | gi|62241063|dbj|BAD93688.1| | AGCCGTTGAGCTTACAGGTTAC/ TGGTATCAGTGGTGTGGAGAGT | ATGGCTTCAGAACCCAAAAAAGCCCATTTTGTAGTGTTTCCAATGATGCAACAAGGCCACATGATCCCAATGGTAGACATCGCAAGGCTCATAGCAAGGCACGGCACAACCGTCACCATAGTCACCACACCTCTCAACGCTCTCCGTTTCAAAGCCACCCTCGATCACGACGTCGAAACTGAAGCATTGGACATTCGAGTTGCCGAAATCGAATTCCCTTCCAAGGAAGCGGGATTTCCCGAAGGAATCGAAAATGCCGACATGTTGCCTTCCCTCGGTTTGGTCGAATCATTCGTCGTTTCCACCAGCTTGCTTCATGATCAATCTGAGAAGCTTTTCTCAGAGCTTAAGCCCAAACCAACTTGCATTTTGTCCGACGTCATATTTCATTGGACAATTGATATTGCTAAGAGGTATGGTGTTCCAAGAATTGTGTTTCATGGTCTTGGTTGCTTTGCTTTACTCTGCTCTCACAATTTGATCAAGTCTAAGGTTCTTGATAGTATAACCTCTGACACAGAGCAAGTTGTAGTGCCTGATCTGCCAGATGAGATTGTTCTTACCAAAGATCAGCTTCCCAATAGCCTTAGGCCAGGGTCTTCGGCCCTAAAAGATCGAACTACGCGTATTAGAGCAGCTGAAGAAGAATCGTTTGGGATTGTAGTGAATTCTTTCGAGGAATTGGAGGTCGAATATATGAAGAGATACAAAGAGGCGAAGTCGCCAAAGAAGGTTTGGTTTGTTGGTCCTGTTTGCTTGAGCAACAAAGATAATGCAAGCAAGGGTGACAGAGGCAACAAATCTTCTGTTGATGGAAATTTATGTTTGAAATGGCTTGAGAATCGTGAACCGGGTTCTGTAATCTATGCTTGCCATGGAACCCTTTGCTCGTTCAACACGCGACAAATGATGGAGCTCGGTTTAGGCTTGGAGGAATCGAAAAAATCTTTCGTATGGGTTATAAGAAGACGAGACGGGTTGGAGGAACTCGAGAAGTGGATGAAGGAAAGCGGGTTCAAAGAAAGGATTAAAGATAGAGCATTAGTGATTTGGGGATGGGCACCACAAGTGTTGATATTGTCACACCCTTCAATAGGTGGATTCTTGACACACTGTGGATGGAACTCGACTCTAGAAGGGGTGTGCGCGGGTTTGCCAATGGTGACATGGCCAATGTTTTCGGACCAATTCTATAATGAGAAGTTGATTACACAAATATGGAAGATTGGTGTGACAGTCGGAGTGAAGAGTCCTGTGAAGTTTGAGGAAGAGAAAATGTTGTCTATTTTGGTGAAGAAAGAAGACGTTGCGAAGGCGGTGGAGGCGATAATGGAGGACGGAGAAGAAGGGAGGAAGAGAAAGGAAAGGGCAAGAGAGTTGGGAAATATGGCTAGAGAAGCGTTTGGAGAGGGTGGCTCTGCTTATTTGAGTATGAAATCTTTAATCAACGATATTTTACAACAA | CAGGCACGGCACAACCGTCACCATAGTCACCACACCTCTCAACGCTCTCCGTTTCAAAGCCACCCTCGATCACGACGTCGAAACTGAAGCATTGGACATTCGAGTTGCCGAAATCGAATTCCCTTCCAAGGAAGCGGGATTTCCCGAAGGAATCGAAAATGCCGACATGTTGCCTTCCCTCGGTTTGGTCGAATCATTCGTCGTTTCCACCAGCTTGCTTCATGATCAATCTGAGAAGCTTTTCTCAGAGCTTAAGCCCAAACCAACTTGCATTTTGTCCGACGTCATATTTCATTGGACAATTGATATTGCTAAGAGGTATGGTGTTCCAAGAATTGTGTTTCATGGTCTTGGTTGCTTTGCTTTACTCTGCTCTCACAATTTGATCAAGTCTAAGGTTCTTGATAGTATAACCTCTGACACAGAGCAAGTTGTAGTGCCTGATCTGCCAGATGAGATTGTTCTTACCAAAGATCAGCTTCCCAATAGCCTTAGGCCAGGGTCTTCGGCCCTAAAAGATCGAACTAYGCGTATTAGAGCAGCTGAAGAAGAATCGTTTGGGATTGTAGTGAATTCTTTCGAGGAATTGGAGGTCGAATATATGAAGAGATACAAAGAGGCGAAGTCGCCAAAGAAGGTTTGGTTTGTTGGTCCTGTTTGCTTGAGCAACAAAGATAATGCAAGCAAGGGTGACAGAGGCAACAAATCTTCTGTTGATGGAAATTTATGTTTGAAATGGCTTGAGAATCGTGAACCGGGTTCTGTAATCTATGCTTGCCATGGAACCCTTTGCTCGTTCAACACGCGACAAATGATGGAGCTCGGTTTAGGCTTGGAGGAATCGAAAAAATCTTTCGTATGGGTTATARGAAGACGAGACGGGTTGGAGGAACTCGAGAAGTGGATGARGRAARSSGGKTYSRAAGAAAGGATTAAAGATAGAGCATTAGTGATTTGGGGATGGGCACCACAAGTGTTGATATTGTCACACCCTTCAATAGGTGGATTCTTGACACACTGTGGATGGAACTCGACTCTAGAAGGGGTGTGCGCGGGTTTGCCAATGGTGACATGGCCAATGTTTTCGGACCAATTCTATAATGAGAAGTTGATTACACAAATATGGAAGATTGGTGTGACAGTCGGAGTGAAGAGTCCTGTGAAGTTTGAGGAAGAGAAAATGTTGTCTATTTTGGTGAAGAAAGAAGACGTTGCGAAGGCGGTGGAGGCGATAATGGAGGACGGAGAAGAAGGGAGGAAGAGA | 1264 | 99.05 |
| **9** | Unigene15235_All | Cytochrome P450 | Glycine max | gi|225458057|ref|XP_002278387.1| | CGGTCATCGGTAACCTCCAC/  TGTTTCCTTCACCACTGCCTTC | CTCTTCTTGCAGCTTCTTCTTGCACTCTTTGTTGCTTCCTTTATCTTCCTCATACAAAAGAATAAGAAAAAAGCTAGTCAACCTCCGGGGCCACCTGGGCTTCCGGTCATCGGTAACCTCCACCAGTGTGCTTCCACTACCCCTCACCTCTACTTTCATGAACTATGCAAAACCTACGGGCCCTTAATGTCTTTGAAACTCGGGTTTGTACCAACCCTTGTGGTTTCCTCGGCAAAAATGGCCAAGGAAGTACTGAAAACCCATGACAACGTCTTCGCTAGCAGGCCAAAGTTGTTAAATCAACAAAAACTATTTTACAATGGGTTAAGCATAACCCTATTACCCTATGGCGATCACTGGAGAGAAATTAGGAAAGCCTGTATAGTTCATCTCTTTAGTCCTAAGAGAGTCCAGTCCTTCTCTTCAATTCGAGAAGATGAGGTTTATCAGATGACGCGGGAGATATCGTGTTTGGGCAATAAAAAGAAGCCGGTAAATTTAAGTGAAATGATGATATGCGTTATCAACACAATAGTATGTAGAGTTGCTTTTGGGAGGAGGGAAGAGGTTGATAATGCCAAGTATAGAAGTAATCTCCAAGTCATGCTTAGGGTTATACAAGAATTGCTTGTTAGTTTTTCTTTTTCCGATTATTTTCCTCTTATGGGTTGGGTGGATAAGATCAGGAGGCTAATGCATCCCCAATTCAATAAGGTTTCTATGGAGTTGGATGGCCTTCTTGAGGAAATCATTGACGAGCATGTGAAGACAAAATCTAGCCGAGAAGAAGAGGAAGACATTGTTGATGTGCTACTCAGGCTCCAACGACAGAATTCATTTGGATTCGACATTAACCGTGATCACATCAAAGCAACTCTTATGGATTTATTCGTGGCAGGGACAGATACAGCTGCAGGAACAATAGTTTGGGCAATGACGGAGCTTATGAGGAATCCAAAGGCAATGAAGCAACTGCAAGAAGAAGCAAGAACAACATTGATGGGTAAAGACACTATAAAAGAAGCAGATCTTGGAAAGCTAGTGTATTTGAAGGCAGTGGTGAAGGAAACATTGAGATTGCACCCGGCAGCTCCATTGCTAGTCCCCCGAGAAGCCAATCAAAGTTGCATAATTGACGGGTACGAAATCAAAAAGAAAACCTTAGTCTATGTAAATGCATATGCAATTGGAAGGGACCCTGAAGCCTGGGAAAACCCGGAT | TCCGTCACACGGGGGTCTCACCAGTGTGCTCACTACCCTCACTCTAYKWWCWWKRCWAWRCMWACKRSSSSYYWAWKKYYTTGAAACTCGGGTTTGTACMAMCYSTKGTGGTTTCCTCGGCAAAAATGGCCAAGGAAGTACTGAAAACCCATGACAACGTCTTCGCTAGCAGGCCAAAGTTGTTAAATCAACAAAAACTATTTTACAATGGGTTAAGCATAACCCTATTACCCTATGGCGATCACTGGAGAGAAATTAGGAAAGCCTGTATAGTTCATCTCTTTAGTCCTAAGAGAGTCCAGTCCTTCTCTTCAATTCGAGAAGATGAGGTTTATCAGATGACGCGGGAGATATCGTGTTTGGGCAATAAAAAGAAGCCGGTAAATTTAAGTGAAATGATGATATGCGTTATCAACACAATAGTATGTAGAGTTGCTTTTGGGAGGAGGGAAGAGGTTGATAATGCCAAGTATAGAAGTAATCTCCAAGTCATGCTTAGGGTTATACAAGAATTGCTTGTTAGTTTTTCTTTTTCCGATTATTTTCCTCTTATGGGTTGGGTGGATAAGATCAGGAGGCTAATGCATCCCCAATTCAATAAGGTTTCTATGGAGTTGGATGGCCTTCTTGAGGAAATCATTGACGAGCATGTGAAGACAAAATCTAGCCGAGAAGAAGAGGAAGACATTGTTGATGTGCTACTCAGGCTCCAACGACAGAATTCATTTGGATTCGACATTAACCGTGATCACATCAAAGCAACTCTTATGGATTTATTCGTGGCAGGGACAGATACAGCTGCAGGAACAATAGTTTGGGCAATGACGGAGCTTATGAGGAATCCAAAGGCAATGAAGCAACTGCAAGAAGAAGCAAGAACAACATTGATGGGTAAAGACACTATAAAAGAAGCAGATCTTGAAAGCTAGTGTATTTGAAGGATGTGGG | 948 | 93.54 |
| **10** | CL1442.Contig1_All | Cytochrome P450 | Populus trichocarpa | gi|224089905|ref|XP_002308860.1| | GAACGACGAGGGCGTAATC/  GCAACCTTGGAAGAGGGACT | CTCCTCCTCATCACCGTCCTTCTCCTCACCGTCGTCCTCTCCTACCTCCTCTACAACCGCCTCCGGTTCAAGCTCCCGCCGGGCCCAAGGCCCTGGCCCGTCGTCGGGAACCTCTACGACATCAAGCCCGTTCGCTTCCGTTGCTTCCACGAATGGGCCCAACGCTACGGGCCCATCATATCGGTGTGGTTCGGGTCCAACCTCAACGTGATCGTGTCGAACACCGAGCTGGCTCGGGAGGTGCTCAAGGAGAAGGACCAGCAGCTGGCGGACCGCCACCGGAGCCGGTCAGCGGTCCGGTTCAGCCGCAACGGGGCGGATCTCATATGGGCCGACTACGGGCCTCACTATGTGAAGGTGAGGAAGGTGTGTACGTTGGAGCTTTTTACGCCTAAGAGGCTTGAATCTCTTAGGCCCGTTAGGGAGGATGAGGTTACCGCCATGATTGAATCCATCTACAAAGATTGCACCTCTCATGAAACCCATGGGAAGAGCCTGACGCTGAAGAAATACTTAGGAGCCGTGGCCTTCAACAACATAACAAGGCTCGCCTTTGGGAAGCGGTTCATGAACGACGAGGGCGTAATCAACAAGCAAGGGCTCGAGTTCAAGGCCATTGTAGCCAACGGCTTGAAGCTTGGGGCGTCTTTGGCCATGTCGGAGCACATTAACTGGCTCCGGTGGATGTACCCGGTTGAGGAGGAGGCCTTTGCCAAGCACAACGCCCAAAGGGACCGTCTCACAAAGGCCATCATGGATGAGCACACCGCCGCTCGCCAAAAGAGTGGTGACACAAAACAACACTTTGTTGATGCTTTGTTGACACTCAAGGACAAGTATGATCTTAGTGAAGACACCATTATTGGACTTCTTTGGGACATGATCACGGCCGGTATGGATACAACAGCAATCTCAGTAGAATGGGCCATGGCGGAGCTAATCCGAAACCCAAGGACCCAACAAAAGGCCCAAGAAGAGCTTGACCGGGTCATCGGGCATGAACGAATCATGATCGAATCCGACATCTCCAACCTCCCTTACTTGCAAGCTGTGGCCAAGGAGGCGCTCAGGCTACACCCACCAACGCCACTAATGCTCCCCCACCGAGCCAACGCCAACGTAAAAATCGGGGGCTATGACATCCCGAAAGGCTCCATTGTCCAAGTCAACGTGTGGGCTGTGGCCCGTGACCCGGCTGTGTGGAAGGACCCGCTCGAGTTCAGGCCCGAAAGGTACTTTGAGGAGGATGTTGACATGAAAGGGCACGACTTTAGGTTATTGCCTTTCGGGGCGGGTCGACGGGTCTGCCCAGGTGCCCAGCTCGGTATAAACTTGGTCACCTCCATGTTGGGTCATTTGCTGCACCATTTTAGCTGGAGCCCACCCAACGGGCTTAAGCCCGAAGAGATTGACATGTCGGAGAATCCCGGGTTGGTTACATATATGACTTCTCCGTTGCAGACAGTCCCTCTTCCAAGGTTGCCTTCTCAGTTGTACAAAAGGGTACCTGTGGATATG | GTATCAACAAGCAAGGGCTCGAGTTCAAGGCCATTGTAGCCAACGGCTTGAAGCTTGGGGCGTCTTTGGCCATGTCGGAGCACATTAACTGGCTCCGGTGGATGTACCCGGTTGAGGAGGAGGCCTTTGCCAAGCACAACGCCCAAAGGGACCGTCTCACAAAGGCCATCATGGATGAGCACACCGCCGCTCGCCAAAAGAGTGGTGACACAAAACAACACTTTGTTGATGCTTTGTTGACACTCAAGGACAAGTATGATCTTAGTGAAGACACCATTATTGGACTTCTTTGGGACATGATCACGGCCGGYATGGACACAACAGCAATCTCAGTAGAATGGGCCATGGCGGAGCTAATCCGAAACCCAAGGACCCAACAAAAGGCCCAAGAAGAGCTTGACCGGGTCATCGGGCATGAACGAATCATGATTGAATCCGACATCTCCAACCTCCCTTACTTGCAAGCTGTGGCCAAGGAGGCGCTCAGGCTACACCCACCAACGCCACTAATGCTCCCCCACCGAGCCAATGCCAACGTAA  AAATCGCGGGCTATGACATCCCGAAAGGCTCCATTGTCCAAGTCAACGTGTGGGCTGTGGCCCGTGACCCGGCTGTGTGGAAGGACCCGCTCGAGTTCAGGCCCGAAAGGTACTTTGAGGAGGATGTTGACATGAAAGGGCACGACTTTAGGTTATTGCCTTTCGGGGCGGGTCGACGGGTCTGCCCAGGTGCCCAGCTCGGTATAAACTTGGTCACCTCCATGTTRGGTCATTTGCTGCACCATTTTAGCTGGAGCCCGCCCGACGGGCTTAAGCCCGAAGAGATCGACATGTCGGAGAATCCCGGGTTGGTTACATATATGACTTCTCCGTTGCAGACAGTCCTCTCTCACAAGGTGTGTCCAA | 906 | 97.35 |
| **11** | Unigene19863_All | stilbene synthase | Fallopia multiflora | gi|401710284|gb|AFP97667.1| | AGATGAGGAAGAAGTCGCTTGA/  TTCGTAGCACCACAGTTTCA | GATGAGATGAGGAAGAAGTCGCTTGAAAACGGTCAAGCAACCACCGGAGAAGGACTTGAGTGGGGCGTTCTGTTTGGATTTGGGCCTGGTATTACTGTTGAAACTGTGGTGCTACGAAGTGTGCCCATCTTT | AGGACTTGAGTGGGGCGTTCTGTTTGGATTTGGGCCTGGTATTACTGTTGAAACTGTGGTGCTACGAAA | 69 | 98.55 |
| **12** | CL4799.Contig3_All | type III polyketide synthase | Polygonum cuspidatum | gi|163867088|gb|ABY47640.1| | GAGTGAACACCTCACCCACCTC/  TTCCCAAGCTCTGCAACTCCTT | ATGGAGGCTTCAATTGAGGAGATTAGGAAGGCACAAACACCCGCTACCGTCCTGGCCATCGGCACCGCCAACCCTCCCAACTGCATGTACCAAGCCGACTTTCCCGATTATTACTTCCGCATCACCAAGAGTGAACACCTCACCCACCTCAAGCAAAAATTCAAGCGCATTTGTGATAATTCAATGATCGAGAAGCGTTACTTTCAATTGACCGAAGAGATTATCAAGGAAAACCCAAATATTGGTGCCTATGAGGCACCGTCATTGAATGCAAGACACAAAATTCAAGTGAAAGGAGTTGCAGAGCTTGGGAAAGAG | TAAGAGTGAACACCTCACCCACCTCAAGCACAAATTCAAGCGCATTTGTGAGAAGTCAATGATCGAGAAGCGTTACCTTCAATTGACGGAAGAAATTCTCAAAGAAAACCCGAATATCGG  TGCATACGAGGCACCATCATTGGATGTAAGACACAAAATTCAGGTGAAAGGAGTTGCAGAGCTTGGGAAA | 190 | 91.05 |
| **13** | Unigene17201_All | stilbene synthase | Polygonum cuspidatum | gi|91984065|gb|ABE68892.1| | GGGAAGAAGACGACCGGAGAT/GAATCGGAACACTTCGCATGAC | GGGAAGAAGACGACCGGAGATGGGTTCGAGTGGGGCGTCGCGATTGGATTGGGGCCTGGGCTTACCGTTGAGACCGTTGTCATGCGAAGTGTTCCGATTCCG | TGGGGCCTGGGCTTACCGTTGAGACCGTTGTCATGCGAAGTGTTCCGATTCA | 52 | 98.08 |
