# Supplementary material for: Deep sequencing reveals transcriptome re-programming of Polygonum multiflorum thunb. roots to the elicitation with methyl jasmonate
Source: Mol Genet Genomics. 2015 Sep 5;291:337–48. doi: 10.1007/s00438-015-1112-9 (PMC4729805; doi:10.1007/s00438-015-1112-9)
Supplement: Supplementary file 15 — Supplementary material 15 (DOC 209 kb) [file 438_2015_1112_MOESM15_ESM.doc]

**Table S8 Unigenes coding for hydroxylase and UDP-glucosyltransferase in *Polygonum multiflorum* Thunb. root RNA-seq data.**

| Putative unigenes coding for hydroxylase | | | | | | |
| --- | --- | --- | --- | --- | --- | --- |
| ♯ | Gene ID | Gene Length | Nr-ID | Nr-annotation | Swissprot-ID | Swissprot-annotation |
| 1 | CL194.Contig1_All | 1784 | gi|255538130|ref|XP_002510130.1| | cytochrome P450, putative [Ricinus communis] >gi|223550831|gb|EEF52317.1| cytochrome P450, putative [Ricinus communis] | sp|Q9FG65|C81D1_ARATH | Cytochrome P450 81D1 OS=Arabidopsis thaliana GN=CYP81D1 PE=2 SV=1 |
| 2 | CL194.Contig2_All | 1773 | gi|225458768|ref|XP_002285105.1| | PREDICTED: isoflavone 2&apos;-hydroxylase-like [Vitis vinifera] | sp|Q9FG65|C81D1_ARATH | Cytochrome P450 81D1 OS=Arabidopsis thaliana GN=CYP81D1 PE=2 SV=1 |
| 3 | CL422.Contig1_All | 1898 | gi|225427085|ref|XP_002276561.1| | PREDICTED: cytochrome P450 76C4 [Vitis vinifera] | sp|Q8VWZ7|C76B6_CATRO | Geraniol 8-hydroxylase OS=Catharanthus roseus GN=CYP76B6 PE=1 SV=1 |
| 4 | CL1442.Contig1_All | 1793 | gi|224089905|ref|XP_002308860.1| | cytochrome P450 [Populus trichocarpa] >gi|183585157|gb|ACC63870.1| coumaroyl 3-hydroxylase [Populus trichocarpa] >gi|222854836|gb|EEE92383.1| cytochrome P450 [Populus trichocarpa] | sp|O48922|C98A2_SOYBN | Cytochrome P450 98A2 OS=Glycine max GN=CYP98A2 PE=2 SV=1 |
| 5 | CL1442.Contig3_All | 1019 | gi|166209291|gb|ABY85195.1| | p-coumaryl-CoA 3&apos;-hydroxylase [Populus alba x Populus grandidentata] | sp|O48922|C98A2_SOYBN | Cytochrome P450 98A2 OS=Glycine max GN=CYP98A2 PE=2 SV=1 |
| 6 | CL1491.Contig2_All | 565 | gi|332071121|gb|AED99879.1| | cytochrome P450, partial [Panax notoginseng] | sp|O48927|C78A3_SOYBN | Cytochrome P450 78A3 OS=Glycine max GN=CYP78A3 PE=2 SV=1 |
| 7 | CL1491.Contig3_All | 1833 | gi|255555751|ref|XP_002518911.1| | cytochrome P450, putative [Ricinus communis] >gi|223541898|gb|EEF43444.1| cytochrome P450, putative [Ricinus communis] | sp|O48927|C78A3_SOYBN | Cytochrome P450 78A3 OS=Glycine max GN=CYP78A3 PE=2 SV=1 |
| 8 | CL1491.Contig4_All | 1785 | gi|255555751|ref|XP_002518911.1| | cytochrome P450, putative [Ricinus communis] >gi|223541898|gb|EEF43444.1| cytochrome P450, putative [Ricinus communis] | sp|O48927|C78A3_SOYBN | Cytochrome P450 78A3 OS=Glycine max GN=CYP78A3 PE=2 SV=1 |
| 9 | CL1912.Contig1_All | 1748 | gi|3059131|emb|CAA04117.1| | cytochrome P450 [Helianthus tuberosus] | sp|P93147|C81E1_GLYEC | Isoflavone 2'-hydroxylase OS=Glycyrrhiza echinata GN=CYP81E1 PE=1 SV=2 |
| 10 | CL2377.Contig1_All | 1587 | gi|224058639|ref|XP_002299579.1| | cytochrome P450 [Populus trichocarpa] >gi|222846837|gb|EEE84384.1| cytochrome P450 [Populus trichocarpa] | sp|D1MI46|C76BA_SWEMU | Geraniol 8-hydroxylase OS=Swertia mussotii GN=CYP76B10 PE=1 SV=1 |
| 11 | CL2377.Contig3_All | 1024 | gi|224058639|ref|XP_002299579.1| | cytochrome P450 [Populus trichocarpa] >gi|222846837|gb|EEE84384.1| cytochrome P450 [Populus trichocarpa] | sp|D1MI46|C76BA_SWEMU | Geraniol 8-hydroxylase OS=Swertia mussotii GN=CYP76B10 PE=1 SV=1 |
| 12 | CL4001.Contig3_All | 1793 | gi|297735522|emb|CBI17962.3| | unnamed protein product [Vitis vinifera] | sp|O81117|C94A1_VICSA | Cytochrome P450 94A1 OS=Vicia sativa GN=CYP94A1 PE=2 SV=2 |
| 13 | CL4008.Contig1_All | 861 | gi|359491194|ref|XP_002276812.2| | PREDICTED: cytochrome P450 71A4 [Vitis vinifera] | sp|Q9STK9|C71AO_ARATH | Cytochrome P450 71A24 OS=Arabidopsis thaliana GN=CYP71A24 PE=2 SV=3 |
| 14 | CL4287.Contig2_All | 1915 | gi|359491192|ref|XP_003634238.1| | PREDICTED: LOW QUALITY PROTEIN: cytochrome P450 71A4-like [Vitis vinifera] | sp|O04164|C71A6_NEPRA | Cytochrome P450 71A6 (Fragment) OS=Nepeta racemosa GN=CYP71A6 PE=2 SV=1 |
| 15 | CL5341.Contig1_All | 1798 | gi|255538130|ref|XP_002510130.1| | cytochrome P450, putative [Ricinus communis] >gi|223550831|gb|EEF52317.1| cytochrome P450, putative [Ricinus communis] | sp|Q9FG65|C81D1_ARATH | Cytochrome P450 81D1 OS=Arabidopsis thaliana GN=CYP81D1 PE=2 SV=1 |
| 16 | CL5341.Contig4_All | 770 | gi|225458768|ref|XP_002285105.1| | PREDICTED: isoflavone 2&apos;-hydroxylase-like [Vitis vinifera] | sp|O65790|C81F1_ARATH | Cytochrome P450 81F1 OS=Arabidopsis thaliana GN=CYP81F1 PE=2 SV=2 |
| 17 | CL5341.Contig5_All | 1653 | gi|255538130|ref|XP_002510130.1| | cytochrome P450, putative [Ricinus communis] >gi|223550831|gb|EEF52317.1| cytochrome P450, putative [Ricinus communis] | sp|Q9FG65|C81D1_ARATH | Cytochrome P450 81D1 OS=Arabidopsis thaliana GN=CYP81D1 PE=2 SV=1 |
| 18 | CL5399.Contig4_All | 860 | gi|297733678|emb|CBI14925.3| | unnamed protein product [Vitis vinifera] | sp|O04164|C71A6_NEPRA | Cytochrome P450 71A6 (Fragment) OS=Nepeta racemosa GN=CYP71A6 PE=2 SV=1 |
| 19 | CL6329.Contig2_All | 1245 | gi|147767047|emb|CAN67678.1| | hypothetical protein VITISV_035274 [Vitis vinifera] | sp|O04164|C71A6_NEPRA | Cytochrome P450 71A6 (Fragment) OS=Nepeta racemosa GN=CYP71A6 PE=2 SV=1 |
| 20 | CL7434.Contig1_All | 1908 | gi|255572197|ref|XP_002527038.1| | cytochrome P450, putative [Ricinus communis] >gi|223533600|gb|EEF35338.1| cytochrome P450, putative [Ricinus communis] | sp|O81117|C94A1_VICSA | Cytochrome P450 94A1 OS=Vicia sativa GN=CYP94A1 PE=2 SV=2 |
| 21 | CL8857.Contig2_All | 1742 | gi|359491192|ref|XP_003634238.1| | PREDICTED: LOW QUALITY PROTEIN: cytochrome P450 71A4-like [Vitis vinifera] | sp|O04164|C71A6_NEPRA | Cytochrome P450 71A6 (Fragment) OS=Nepeta racemosa GN=CYP71A6 PE=2 SV=1 |
| 22 | CL10212.Contig1_All | 1997 | gi|225449669|ref|XP_002264292.1| | PREDICTED: cytochrome P450 94A1-like [Vitis vinifera] | sp|P98188|C94A2_VICSA | Cytochrome P450 94A2 OS=Vicia sativa GN=CYP94A2 PE=2 SV=1 |
| 23 | Unigene3242_All | 1652 | gi|224127430|ref|XP_002320072.1| | cytochrome P450 [Populus trichocarpa] >gi|222860845|gb|EEE98387.1| cytochrome P450 [Populus trichocarpa] | sp|Q50EK3|C04C1_PINTA | Cytochrome P450 704C1 OS=Pinus taeda GN=CYP704C1 PE=2 SV=1 |
| 24 | Unigene6328_All | 433 | gi|356567371|ref|XP_003551894.1| | PREDICTED: cytochrome P450 83B1-like [Glycine max] | sp|E3W9C4|C71A1_ZINZE | Alpha-humulene 10-hydroxylase OS=Zingiber zerumbet GN=CYP71BA1 PE=1 SV=1 |
| 25 | Unigene7711_All | 1945 | gi|255576331|ref|XP_002529058.1| | cytochrome P450, putative [Ricinus communis] >gi|223531470|gb|EEF33302.1| cytochrome P450, putative [Ricinus communis] | sp|O81117|C94A1_VICSA | Cytochrome P450 94A1 OS=Vicia sativa GN=CYP94A1 PE=2 SV=2 |
| 26 | Unigene12763_All | 910 | gi|255641226|gb|ACU20890.1| | unknown [Glycine max] | sp|D1MI46|C76BA_SWEMU | Geraniol 8-hydroxylase OS=Swertia mussotii GN=CYP76B10 PE=1 SV=1 |
| 27 | Unigene12765_All | 910 | gi|356968416|gb|AET43289.1| | CYP76AD1 [Beta vulgaris] >gi|356968418|gb|AET43290.1| CYP76AD1 [Beta vulgaris] | sp|D1MI46|C76BA_SWEMU | Geraniol 8-hydroxylase OS=Swertia mussotii GN=CYP76B10 PE=1 SV=1 |
| 28 | Unigene14525_All | 1676 | gi|3059131|emb|CAA04117.1| | cytochrome P450 [Helianthus tuberosus] | sp|P93147|C81E1_GLYEC | Isoflavone 2'-hydroxylase OS=Glycyrrhiza echinata GN=CYP81E1 PE=1 SV=2 |
| 29 | Unigene15235_All | 1246 | gi|225458057|ref|XP_002278387.1| | PREDICTED: cytochrome P450 71A1 [Vitis vinifera] | sp|O81970|C71A9_SOYBN | Cytochrome P450 71A9 OS=Glycine max GN=CYP71A9 PE=2 SV=1 |
| 30 | Unigene15442_All | 1741 | gi|27529728|dbj|BAC53893.1| | cytochrome P450 [Petunia x hybrida] | sp|Q8VWZ7|C76B6_CATRO | Geraniol 8-hydroxylase OS=Catharanthus roseus GN=CYP76B6 PE=1 SV=1 |
| 31 | Unigene19630_All | 1792 | gi|225453812|ref|XP_002276576.1| | PREDICTED: cytochrome P450 76C4 [Vitis vinifera] | sp|D1MI46|C76BA_SWEMU | Geraniol 8-hydroxylase OS=Swertia mussotii GN=CYP76B10 PE=1 SV=1 |
| 32 | Unigene20299_All | 849 | gi|255547149|ref|XP_002514632.1| | cytochrome P450, putative [Ricinus communis] >gi|223546236|gb|EEF47738.1| cytochrome P450, putative [Ricinus communis] | sp|O65012|C78A4_PINRA | Cytochrome P450 78A4 OS=Pinus radiata GN=CYP78A4 PE=2 SV=1 |
| 33 | Unigene20586_All | 1216 | gi|359489507|ref|XP_003633930.1| | PREDICTED: LOW QUALITY PROTEIN: cytochrome P450 76C4-like [Vitis vinifera] | sp|O64635|C76C4_ARATH | Cytochrome P450 76C4 OS=Arabidopsis thaliana GN=CYP76C4 PE=2 SV=1 |
| 34 | Unigene20977_All | 420 | gi|357125480|ref|XP_003564422.1| | PREDICTED: cytochrome P450 94A1-like [Brachypodium distachyon] | sp|O81117|C94A1_VICSA | Cytochrome P450 94A1 OS=Vicia sativa GN=CYP94A1 PE=2 SV=2 |
| 35 | Unigene23516_All | 1965 | gi|359491185|ref|XP_002276558.2| | PREDICTED: cytochrome P450 71A1-like [Vitis vinifera] | sp|P24465|C71A1_PERAE | Cytochrome P450 71A1 OS=Persea americana GN=CYP71A1 PE=1 SV=2 |
| 36 | Unigene24860_All | 478 | gi|3059129|emb|CAA04116.1| | cytochrome P450 [Helianthus tuberosus] | sp|Q9FG65|C81D1_ARATH | Cytochrome P450 81D1 OS=Arabidopsis thaliana GN=CYP81D1 PE=2 SV=1 |
| 37 | Unigene25824_All | 1764 | gi|255538130|ref|XP_002510130.1| | cytochrome P450, putative [Ricinus communis] >gi|223550831|gb|EEF52317.1| cytochrome P450, putative [Ricinus communis] | sp|Q9FG65|C81D1_ARATH | Cytochrome P450 81D1 OS=Arabidopsis thaliana GN=CYP81D1 PE=2 SV=1 |
| 38 | Unigene26381_All | 780 | gi|356968416|gb|AET43289.1| | CYP76AD1 [Beta vulgaris] >gi|356968418|gb|AET43290.1| CYP76AD1 [Beta vulgaris] | sp|D1MI46|C76BA_SWEMU | Geraniol 8-hydroxylase OS=Swertia mussotii GN=CYP76B10 PE=1 SV=1 |
| 39 | Unigene28654_All | 515 | gi|224067242|ref|XP_002302426.1| | cytochrome P450 [Populus trichocarpa] >gi|222844152|gb|EEE81699.1| cytochrome P450 [Populus trichocarpa] | sp|O65790|C81F1_ARATH | Cytochrome P450 81F1 OS=Arabidopsis thaliana GN=CYP81F1 PE=2 SV=2 |
| 40 | Unigene33958_All | 490 | gi|225453799|ref|XP_002276053.1| | PREDICTED: cytochrome P450 76C4-like [Vitis vinifera] | sp|O64635|C76C4_ARATH | Cytochrome P450 76C4 OS=Arabidopsis thaliana GN=CYP76C4 PE=2 SV=1 |
| 41 | Unigene39885_All | 1724 | gi|255538130|ref|XP_002510130.1| | cytochrome P450, putative [Ricinus communis] >gi|223550831|gb|EEF52317.1| cytochrome P450, putative [Ricinus communis] | sp|Q9FG65|C81D1_ARATH | Cytochrome P450 81D1 OS=Arabidopsis thaliana GN=CYP81D1 PE=2 SV=1 |
| 42 | Unigene40223_All | 882 | gi|255641226|gb|ACU20890.1| | unknown [Glycine max] | sp|Q8VWZ7|C76B6_CATRO | Geraniol 8-hydroxylase OS=Catharanthus roseus GN=CYP76B6 PE=1 SV=1 |
| 43 | Unigene43261_All | 697 | gi|296089103|emb|CBI38806.3| | unnamed protein product [Vitis vinifera] | sp|O64635|C76C4_ARATH | Cytochrome P450 76C4 OS=Arabidopsis thaliana GN=CYP76C4 PE=2 SV=1 |
| 44 | Unigene43262_All | 420 | gi|356566842|ref|XP_003551635.1| | PREDICTED: cytochrome P450 76C4-like [Glycine max] | sp|Q8VWZ7|C76B6_CATRO | Geraniol 8-hydroxylase OS=Catharanthus roseus GN=CYP76B6 PE=1 SV=1 |
| Putative unigenes coding for UDP-glycosyltransferase | | | | | | |
| ♯ | Gene ID | Gene Length | Nr-ID | Nr-annotation | Swissprot-ID | Swissprot-annotation |
| 1 | CL554.Contig1_All | 1834 | gi|62241063|dbj|BAD93688.1| | glucosyltransferase [Nicotiana tabacum] | sp|Q9ZQ95|U73C6_ARATH | UDP-glycosyltransferase 73C6 OS=Arabidopsis thaliana GN=UGT73C6 PE=2 SV=1 |
| 2 | CL554.Contig3_All | 1773 | gi|62241063|dbj|BAD93688.1| | glucosyltransferase [Nicotiana tabacum] | sp|Q9ZQ95|U73C6_ARATH | UDP-glycosyltransferase 73C6 OS=Arabidopsis thaliana GN=UGT73C6 PE=2 SV=1 |
| 3 | CL2466.Contig2_All | 983 | gi|209954731|dbj|BAG80556.1| | UDP-glucose:glucosyltransferase [Lycium barbarum] | sp|Q9AR73|HQGT_RAUSE | Hydroquinone glucosyltransferase OS=Rauvolfia serpentina GN=AS PE=1 SV=1 |
| 4 | CL2476.Contig1_All | 2113 | gi|359488135|ref|XP_002268383.2| | PREDICTED: UDP-glycosyltransferase 89A2-like [Vitis vinifera] | sp|Q9LZD8|U89A2_ARATH | UDP-glycosyltransferase 89A2 OS=Arabidopsis thaliana GN=UGT89A2 PE=2 SV=1 |
| 5 | CL2476.Contig2_All | 425 | gi|255547035|ref|XP_002514575.1| | UDP-glucosyltransferase, putative [Ricinus communis] >gi|223546179|gb|EEF47681.1| UDP-glucosyltransferase, putative [Ricinus communis] | sp|Q9LZD8|U89A2_ARATH | UDP-glycosyltransferase 89A2 OS=Arabidopsis thaliana GN=UGT89A2 PE=2 SV=1 |
| 6 | CL2671.Contig3_All | 1839 | gi|209954731|dbj|BAG80556.1| | UDP-glucose:glucosyltransferase [Lycium barbarum] | sp|Q9AR73|HQGT_RAUSE | Hydroquinone glucosyltransferase OS=Rauvolfia serpentina GN=AS PE=1 SV=1 |
| 7 | CL4044.Contig3_All | 1001 | gi|110932098|gb|ABH03018.1| | resveratrol/hydroxycinnamic acid O-glucosyltransferase [Vitis labrusca] | sp|Q66PF4|CGT_FRAAN | Cinnamate beta-D-glucosyltransferase OS=Fragaria ananassa GN=GT2 PE=1 SV=1 |
| 8 | CL4508.Contig1_All | 1554 | gi|259563723|gb|ACW83060.1| | glycosyltransferase family GT8 protein [Populus deltoides] | sp|Q9LN68|GATL1_ARATH | Probable galacturonosyltransferase-like 1 OS=Arabidopsis thaliana GN=GATL1 PE=2 SV=1 |
| 9 | CL5221.Contig2_All | 2061 | gi|356528218|ref|XP_003532702.1| | PREDICTED: probable dolichyl pyrophosphate Glc1Man9GlcNAc2 alpha-1,3-glucosyltransferase-like [Glycine max] | sp|O80505|ALG8_ARATH | Probable dolichyl pyrophosphate Glc1Man9GlcNAc2 alpha-1,3-glucosyltransferase OS=Arabidopsis thaliana GN=At2g44660 PE=2 SV=3 |
| 10 | CL5403.Contig3_All | 689 | gi|224121446|ref|XP_002318584.1| | predicted protein [Populus trichocarpa] >gi|222859257|gb|EEE96804.1| predicted protein [Populus trichocarpa] | sp|Q9C9B0|U89B1_ARATH | UDP-glycosyltransferase 89B1 OS=Arabidopsis thaliana GN=UGT89B1 PE=2 SV=2 |
| 11 | CL5630.Contig4_All | 1760 | gi|14192682|gb|AAK54465.1| | cold-induced glucosyl transferase [Solanum sogarandinum] | sp|O23406|U75D1_ARATH | UDP-glycosyltransferase 75D1 OS=Arabidopsis thaliana GN=UGT75D1 PE=2 SV=2 |
| 12 | CL5892.Contig1_All | 1868 | gi|255547075|ref|XP_002514595.1| | UDP-glucosyltransferase, putative [Ricinus communis] >gi|223546199|gb|EEF47701.1| UDP-glucosyltransferase, putative [Ricinus communis] | sp|Q9C9B0|U89B1_ARATH | UDP-glycosyltransferase 89B1 OS=Arabidopsis thaliana GN=UGT89B1 PE=2 SV=2 |
| 13 | CL5892.Contig2_All | 1887 | gi|255547075|ref|XP_002514595.1| | UDP-glucosyltransferase, putative [Ricinus communis] >gi|223546199|gb|EEF47701.1| UDP-glucosyltransferase, putative [Ricinus communis] | sp|Q9C9B0|U89B1_ARATH | UDP-glycosyltransferase 89B1 OS=Arabidopsis thaliana GN=UGT89B1 PE=2 SV=2 |
| 14 | CL7754.Contig1_All | 1813 | gi|297735333|emb|CBI17773.3| | unnamed protein product [Vitis vinifera] | sp|P51094|UFOG_VITVI | Anthocyanidin 3-O-glucosyltransferase 2 OS=Vitis vinifera GN=UFGT PE=1 SV=2 |
| 15 | CL8155.Contig1_All | 1505 | gi|332071136|gb|AED99886.1| | glycosyltransferase [Panax notoginseng] | -- | -- |
| 16 | CL8199.Contig1_All | 1590 | gi|51705413|gb|AAU09444.1| | UDP-glucose glucosyltransferase [Fragaria x ananassa] | sp|Q2V6K0|UFOG6_FRAAN | UDP-glucose flavonoid 3-O-glucosyltransferase 6 OS=Fragaria ananassa GN=GT6 PE=1 SV=1 |
| 17 | CL11757.Contig1_All | 1879 | gi|110932098|gb|ABH03018.1| | resveratrol/hydroxycinnamic acid O-glucosyltransferase [Vitis labrusca] | sp|Q66PF4|CGT_FRAAN | Cinnamate beta-D-glucosyltransferase OS=Fragaria ananassa GN=GT2 PE=1 SV=1 |
| 18 | Unigene1793_All | 630 | gi|204022236|dbj|BAG71126.1| | glucosyltransferase [Phytolacca americana] >gi|219566994|dbj|BAH05015.1| glucosyltransferase [Phytolacca americana] | sp|Q2V6K0|UFOG6_FRAAN | UDP-glucose flavonoid 3-O-glucosyltransferase 6 OS=Fragaria ananassa GN=GT6 PE=1 SV=1 |
| 19 | Unigene2635_All | 2733 | gi|225458916|ref|XP_002285486.1| | PREDICTED: probable xyloglucan glycosyltransferase 12 [Vitis vinifera] | sp|Q9ZQB9|CSLCC_ARATH | Probable xyloglucan glycosyltransferase 12 OS=Arabidopsis thaliana GN=CSLC12 PE=1 SV=1 |
| 20 | Unigene4450_All | 1436 | gi|224098485|ref|XP_002311191.1| | predicted protein [Populus trichocarpa] >gi|222851011|gb|EEE88558.1| predicted protein [Populus trichocarpa] | sp|Q9LFP3|GLYT4_ARATH | Probable glycosyltransferase At5g11130 OS=Arabidopsis thaliana GN=At5g11120/At5g11130 PE=3 SV=2 |
| 21 | Unigene6757_All | 969 | gi|14349253|dbj|BAB60721.1| | glucosyltransferase [Nicotiana tabacum] | sp|Q2V6K0|UFOG6_FRAAN | UDP-glucose flavonoid 3-O-glucosyltransferase 6 OS=Fragaria ananassa GN=GT6 PE=1 SV=1 |
| 22 | Unigene7583_All | 1125 | gi|255546311|ref|XP_002514215.1| | ceramide glucosyltransferase, putative [Ricinus communis] >gi|223546671|gb|EEF48169.1| ceramide glucosyltransferase, putative [Ricinus communis] | -- | -- |
| 23 | Unigene8987_All | 201 | gi|359488135|ref|XP_002268383.2| | PREDICTED: UDP-glycosyltransferase 89A2-like [Vitis vinifera] | sp|Q9C9B0|U89B1_ARATH | UDP-glycosyltransferase 89B1 OS=Arabidopsis thaliana GN=UGT89B1 PE=2 SV=2 |
| 24 | Unigene14565_All | 1619 | gi|359478218|ref|XP_003632087.1| | PREDICTED: UDP-glycosyltransferase 74E2-like [Vitis vinifera] | sp|P0C7P7|U74E1_ARATH | UDP-glycosyltransferase 74E1 OS=Arabidopsis thaliana GN=UGT74E1 PE=3 SV=1 |
| 25 | Unigene14931_All | 710 | gi|357474983|ref|XP_003607777.1| | Anthocyanidin 5 3-O-glucosyltransferase [Medicago truncatula] >gi|355508832|gb|AES89974.1| Anthocyanidin 5 3-O-glucosyltransferase [Medicago truncatula] | sp|Q9C660|PEK10_ARATH | Proline-rich receptor-like protein kinase PERK10 OS=Arabidopsis thaliana GN=PERK10 PE=1 SV=2 |
| 26 | Unigene15430_All | 1753 | gi|255564531|ref|XP_002523261.1| | UDP-glucosyltransferase, putative [Ricinus communis] >gi|223537474|gb|EEF39100.1| UDP-glucosyltransferase, putative [Ricinus communis] | sp|Q9LVW3|U79B1_ARATH | UDP-glycosyltransferase 79B1 OS=Arabidopsis thaliana GN=UGT79B1 PE=2 SV=1 |
| 27 | Unigene18031_All | 1475 | gi|387135224|gb|AFJ52993.1| | UDP-glycosyltransferase 1 [Linum usitatissimum] | sp|Q9LMF1|U85A3_ARATH | UDP-glycosyltransferase 85A3 OS=Arabidopsis thaliana GN=UGT85A3 PE=2 SV=2 |
| 28 | Unigene18687_All | 393 | gi|46430997|gb|AAS94330.1| | UDP-glucose:flavonoid-O-glucosyltransferase [Beta vulgaris] | sp|Q2V6K0|UFOG6_FRAAN | UDP-glucose flavonoid 3-O-glucosyltransferase 6 OS=Fragaria ananassa GN=GT6 PE=1 SV=1 |
| 29 | Unigene19181_All | 320 | gi|339715876|gb|AEJ88222.1| | UDP-glucose:flavonoid 3-O-glucosyltransferase [Prunus persica] | sp|Q5NTH0|UGAT_BELPE | Cyanidin-3-O-glucoside 2-O-glucuronosyltransferase OS=Bellis perennis GN=UGAT PE=1 SV=1 |
